# Breastfeeding and Neurodevelopmental Outcomes in Children of Women Taking Antiseizure Medications

**DOI:** 10.1001/jamanetworkopen.2026.37040

**Published:** 2026-09-30

**Authors:** Yi Li, David W. Loring, Morris J. Cohen, Elizabeth E. Gerard, Angela K. Birnbaum, Abigail G. Matthews, Page B. Pennell, Kimford J. Meador

**Affiliations:** 1 Stanford University, Palo Alto, California; 2 Emory University School of Medicine, Atlanta, Georgia; 3 Pediatric Neuropsychology International, Augusta, Georgia; 4 Feinberg School of Medicine, Northwestern University, Chicago, Illinois; 5 University of Minnesota, Minneapolis; 6 The Emmes Company, Rockville, Maryland; 7 University of Pittsburgh School of Medicine, Pittsburgh, Pennsylvania

## Abstract

**Importance:**

Concerns regarding infant exposure to antiseizure medications (ASMs) via breast milk frequently discourage breastfeeding in women with epilepsy (WWE). Longitudinal neurodevelopmental data for breastfeeding while taking contemporary ASM regimens are essential for evidence-based management.

**Objective:**

To evaluate the association between breastfeeding duration and neurodevelopmental outcomes at age 6 years in children exposed to contemporary ASM regimens.

**Design, Setting, and Participants:**

The Maternal Outcomes and Neurodevelopmental Effects of Antiepileptic Drugs study is a prospective, multicenter, cohort study. Pregnant WWE and healthy pregnant women were enrolled from December 2012 to October 2016, with children followed up to age 6 years. Children’s outcomes were analyzed between January 2023 and December 2024.

**Exposures:**

Breastfeeding duration (none, <6 months, ≥6 months) and children’s exposure to contemporary ASMs (primarily levetiracetam and lamotrigine).

**Main Outcomes and Measures:**

The primary outcomes at child age 6 years included verbal language (Verbal Index Score) and adaptive behavior (General Adaptive Composite score in Adaptive Behavior Assessment System Scale).

**Results:**

A total of 273 children of WWE (median [range] maternal age, 31.0 [19.0-46.0] years; 127 [46.5%] male) and 84 children of healthy women (median maternal age, 31.0 [15.0-38.0] years; 47 [56.0%] male) were included, with 262 children of WWE and 80 children of healthy women in the language outcome sample and 261 children of WWE and 83 children of healthy women in the behavioral outcome set. Adjusted mean (SD) verbal language (107.93 [0.71] vs 107.18 [1.40]; difference, 0.75; 95% CI, −2.40 to 3.89; *P* = .64) and adaptive behavior (102.91 [0.75] vs 102.14 [1.46]; difference, 0.78; 95% CI, −2.52 to 4.07; *P* = .64) outcomes did not significantly differ between groups. Within the WWE cohort, breastfeeding for 6 months or longer was significantly associated with higher adaptive behavioral scores compared with no breastfeeding (estimate, 4.68; 95% CI, 1.34 to 8.03; *P* = .01), driven by practical and conceptual subdomains; whereas shorter durations (<6 months) did not differ. Neurodevelopmental outcomes were comparable within each specific ASM regimen—including levetiracetam, lamotrigine, other monotherapy, and polytherapy—regardless of breastfeeding duration. Notably, the benefit associated with breastfeeding for 6 months or longer remained significant in the pooled monotherapy group (difference, 5.88; 95% CI, 2.21 to 9.55; *P* = .001) but not in the polytherapy group.

**Conclusions and Relevance:**

In this cohort study of children born to WWE, breastfeeding while taking contemporary ASM regimens was not associated with adverse neurodevelopmental outcomes at age 6 years. The observed improved adaptive behavioral outcome supports clinical recommendations for extended breastfeeding (≥6 months) in WWE.

## Key Points

**Question:**

Is breastfeeding while taking contemporary antiseizure medications (ASMs) associated with children’s neurodevelopmental outcomes, and does duration of breastfeeding matter?

**Findings:**

In this cohort study of 262 children of women with epilepsy and 80 children of healthy women, 6-year neurodevelopmental outcomes were comparable. Breastfeeding for 6 months or longer was associated with significantly improved adaptive behavior compared with no breastfeeding, while shorter durations did not differ.

**Meaning:**

In this study, breastfeeding while taking ASMs was not associated with adverse neurodevelopmental outcomes; findings suggest that breastfeeding while taking ASMs should be encouraged, as durations of 6 months or longer were associated with better adaptive behavioral outcomes.

## Teaser

This cohort study examines whether children who are exposed to antiseizure medications including levetiracetam and lamotrigine via breast milk have different neurodevelopmental outcomes at age 6 years than children without such exposures.

## Introduction

Epilepsy is among the most prevalent chronic neurological conditions, affecting approximately 50 million individuals worldwide, with a higher incidence reported in low- and middle-income regions.^1^ Of these, an estimated 12.5 million are women aged to 15 to 49 years,^1^ a demographic that has been steadily increasing globally.^2^ Current estimates suggest that 3 to 5 per 1000 births are to women with epilepsy (WWE), representing a population requiring specialized clinical attention. This is due to the complexity of balancing maternal seizure control with antiseizure medications (ASMs) and potential neurodevelopmental risks to their children, as postnatal ASM exposure could potentially cause apoptotic neurodegeneration,^3^ disrupt synaptic development,^4^ and suppress postnatal neurogenesis^5^ in the rapidly developing brain.

The American Academy of Pediatrics (AAP),^6^ the American College of Obstetricians and Gynecologists (ACOG),^7^ and World Health Organization (WHO)^8^ recommend exclusive breastfeeding for at least the first 6 months of life. Despite the well-documented developmental benefits of breastfeeding in the general population,^9^ WWE are significantly less likely to initiate breastfeeding compared with healthy women (HW).^10,11,12,13,14^ Studies indicate that only 12.1% to 33.1% of WWE achieved this milestone—a rate significantly lower than that of the general population.^11,12^ A primary barrier is the theoretical concern regarding the risk of transfer of ASMs to the infant via breast milk.^15,16,17^ Indeed, ASM exposure is one of the primary factors influencing the decision to initiate and maintain breastfeeding in WWE.^10,13^

Our multicenter prospective Maternal Outcomes and Neurodevelopmental Effects of Antiepileptic Drugs (MONEAD) study found that WWE treated at specialty centers achieved substantially higher breastfeeding initiation rates (74.8% vs 88.8% in HW).^14^ This provided a unique opportunity to evaluate the association between ASM exposure via breast milk and long-term neurodevelopmental outcomes.

Our previous Neurodevelopmental Effects of Antiepileptic Drugs (NEAD) study found that children of WWE breastfed while exposed to ASMs did not exhibit adverse cognitive outcomes compared with nonbreastfed children.^18^ Since then, ASM prescription patterns have shifted significantly toward different medication regimens.^19,20^ High-quality data regarding the long-term cognitive safety of contemporary ASMs during breastfeeding and the impact of breastfeeding duration remain sparce.^17,21^ The current study aimed to compare neurodevelopmental outcomes at age 6 years between children of WWE and HW following breastfeeding exposure, and to evaluate the association between breastfeeding duration and children’s neurodevelopmental outcomes across specific contemporary ASM regimens.

## Methods

### Study Design

The MONEAD study is a prospective, observational cohort study conducted at 20 epilepsy centers across the United States, tracking the neurodevelopmental progress of children born to WWE and healthy women (HW) from pregnancy through 6 years of age.^22,23,24,25,26,27^ The protocol and statistical analysis plan appear in the eAppendix in Supplement 1. Site institutional review boards approved the study, and all adult participants provided written informed consent. This study adheres to the Strengthening the Reporting of Observational Studies in Epidemiology (STROBE) reporting guideline and is registered at ClinicalTrials.gov (NCT01730170).

We hypothesized that breastfeeding would not adversely affect neurodevelopment and that extended breastfeeding would be associated with improved neurodevelopmental outcomes. This study aims to provide evidence on the safety of commonly used contemporary ASM regimens during breastfeeding to support evidence-based, clinical decision-making.

### Participants

Pregnant participants were recruited from December 2012 to October 2016, with child follow-up until age 6 years. HW were recruited via referrals from obstetricians affiliated with the participating centers. Child outcome data were collected and analyzed between January 2023 and December 2024. Eligibility criteria included age 14 to 45 years and gestational age of 20 weeks or less. Key exclusion criteria included a history of psychogenic nonepileptic seizures, maternal IQ less than 70, or medical condition that could independently impact pregnancy or child development (eMethods in Supplement 1). All ASM management was at the discretion of the participants’ personal physicians.

### Exposures

The primary exposure was cumulative breastfeeding duration, categorized as: none, shorter duration (>0 to <6 months), or extended duration (≥6 months), in alignment with the current guideline recommendations.^6,7,8,28,29^ Child ASM exposure was determined by maternal ASM use during breastfeeding, with specific subgroups identified for levetiracetam monotherapy, lamotrigine monotherapy, other monotherapy, and polytherapy.

### Procedures and Outcome Measures

All neuropsychological assessments were administered by trained personnel blinded to maternal group status and child exposure history. Primary objectives were to (1) compare the neurodevelopmental outcomes between breastfed children of WWE receiving contemporary ASMs and breastfed children of HW at age 6 years, assessed using the Verbal Index Score (VIS) for language ability^27^ (eMethods in Supplement 1) and the General Adaptive Composite (GAC) score from the Adaptive Behavior Assessment System, Third Edition (ABAS-3), for adaptive behavioral functioning; and (2) evaluate whether breastfeeding duration was associated with neurodevelopmental outcomes within the WWE cohort. No adjustment of type I error rate for multiple comparisons was applied given that these primary outcomes address different questions.

Secondary analyses evaluated (1) differences between WWE and HW cohorts across additional neurodevelopmental end points, including specific behavioral subdomain outcomes (conceptual, social, and practical) from the ABAS-3 scale; nonverbal ability outcomes; general conceptual ability; and risk of autism spectrum disorder (eMethods in Supplement 1); and (2) the association between the aforementioned secondary neurodevelopmental outcomes and breastfeeding duration (no breastfeeding, >0 to <6 months, and ≥6 months) within the WWE cohort. Given the small sample, exploratory analyses evaluated the association between breastfeeding duration and neurodevelopmental outcomes within the HW cohort.

### Statistical Analysis

Baseline characteristics were compared using χ^2^ or Fisher exact tests for categorical variables and *t* tests or Wilcoxon rank-sum tests for continuous variables. Normality was assessed via visual inspection of histograms and the Shapiro-Wilk test. Nonparametric comparisons across the 3 breastfeeding duration tiers utilized the Kruskal-Wallis test with post hoc analysis.

A multivariable linear regression evaluated the independent association between breastfeeding and continuous outcomes, while logistic regression evaluated binary end points. Covariates were selected based on clinical relevance and established literature regarding child neurodevelopment. These included maternal factors (ie, age, IQ, education, income, race and ethnicity, periconceptional folate dose, and prenatal or postpartum anxiety, depression, sleep quality, and stress scores), perinatal and child factors (gestational age, birth weight, sex, and race and ethnicity), and epilepsy-specific variables (ASM type, fetal ASM concentration, epilepsy type, and seizure frequency during pregnancy). Participant race and ethnicity were identified by maternal self-report and classified into the following categories prespecified in MONEAD: Black, White, and other or unknown. Race and ethnicity data were collected because both are associated with breastfeeding practices and childhood neurodevelopment in the United States and were therefore evaluated as potential confounders. Only completers were included in the primary and secondary analysis. Covariates were identified using a stepwise regression algorithm (entry, *P* < .05; removal, *P* ≥ .10) (eAppendix in Supplement 1).

Sensitivity analyses were conducted to confirm the robustness of the primary findings, including (1) modeling breastfeeding duration as a binary variable (≥6 months vs <6 months); (2) testing for statistical interactions between maternal group (WWE vs HW) and children’s developmental outcomes; (3) repeating primary analyses with one randomly selected child per twin set to account for nonindependence; (4) optimizing stepwise regression via Akaike information criterion (AIC) to ensure retention of the primary exposure; and (5) a propensity score analysis for primary outcomes. Analyses were conducted using Python version 3.11 (Python Software Foundation), R version 4.6 (R Project for Statistical Computing) and SPSS version 28 (IBM Corp), with statistical significance defined by a 2-sided α = .05 (eAppendix in Supplement 1).

## Results

### Cohort Characteristics and Breastfeeding Behaviors

Of 1123 pregnant women screened, 456 were enrolled, including 351 WWE and 105 HW. Of these, 331 WWE and 102 HW successfully completed their pregnancies. After excluding 15 WWE not taking ASMs and applying all exclusion criteria (eFigure 1 in Supplement 1), 273 children of WWE (median [range] maternal age, 31.0 [19.0-46.0] years; 127 [46.5%] male) and 84 children of HW (median maternal age, 31.0 [15.0-38.0] years; 47 [56.0%] male) had at least 1 primary outcome assessment available (Table 1). The final 6-year analysis consisted of 262 children of WWE and 80 children of HW for the language outcome, and 261 children of WWE and 83 children of HW for the behavioral outcome (eTables 1 and 2 in Supplement 1).

**Table 1.** Demographic and Clinical Characteristics of Children With Neurodevelopmental Assessment Available at Age 6 Years

Characteristic	Participants, No./total No. (%)		*P* value
	Women with epilepsy taking ASMs (n = 273)	Healthy women (n = 84)	
Child characteristics			
Child sex			
Female	146/273(53.5)	37/84(44.0)	.14
Male	127/273 (46.5)	47/84 (56.0)	
Unknown/intersex	0/273	0/84	
Race			
Black	11/273 (4.0)	8/84 (9.5)	.01
White	225/273 (82.4)	57/84 (67.9)	
Other^a^	37/273 (13.6)	19/84 (22.6)	
Hispanic ethnicity	56/273 (20.5)	21/84 (25.0)	.45
Gestational age at birth, median (range), wk	39.0 (24.0-42.0)	39.0 (26.0-43.0)	.95
Birth weight, median (range), kg	3.2 (0.6-4.9)	3.3 (1.1-5.0)	.68
Twins	22/273 (8.1)	6/84 (7.1)	>.99
Breastfeeding duration, mo			
0	71/273 (26.0)	11/84 (13.1)	<.001
>0 to <6	77/273 (28.2)	15/84 (17.9)	
≥6	125/273 (45.8)	58/84 (69.0)	
Maternal characteristics			
Maternal age, median (range), y	31.0 (19.0-46.0)	31.0 (15.0-38.0)	.10
Maternal IQ, median (range)	100.0 (65.0-123.0)	105.0 (72.0-131.0)	<.001
Maternal education			
No college degree	75/273 (27.5)	23/84 (27.4)	.41
College degree	126/273 (46.2)	33/84 (39.3)	
Advanced degree	72/273 (26.4)	28/84 (33.3)	
Maternal employment status			
Full-time	151/273 (55.3)	47/84 (56.0)	.56
Part-time	41/273 (15.0)	16/84 (19.0)	
Unemployed	81/273 (29.7)	21/84 (25.0)	
Periconceptional folate dose, mg/d^b^			
0	33/272 (12.1)	27/82 (32.9)	<.001
>0 to 0.4	21/272 (7.7)	11/82 (13.4)	
>0.4 to 1.0	49/272 (18.0)	32/82 (39.0)	
>1.0 to 4.0	147/272 (54.0)	12/82 (14.6)	
>4.0	22/272 (8.1)	0/82	
Pregnancy planning status			
Planned	198/273 (72.5)	57/84 (67.9)	.71
Welcome	67/273 (24.5)	24/84 (28.6)	
Unwelcome	8/273 (2.9)	3/84 (3.6)	

Abbreviation: ASM, antiseizure medication.

^a^ Other race includes any race not otherwise specified.

^b^ Folate data were missing for 1 woman in the women with epilepsy group and 2 women in the healthy control group; percentages are based on available data.

In unadjusted analyses, breastfeeding initiation (202 of 273 [77.1%] vs 73 of 84 [92.5%]) and 6-month duration (125 [45.8%] vs 58 [69.0%]) rates were lower in WWE than HW. However, after adjusting for confounders, maternal epilepsy status was not significantly associated with breastfeeding ≥6 months (odds ratio [OR], 0.63; 95% CI, 0.34-1.16; *P* = .14) (eTable 3 in Supplement 1). Factors associated with breastfeeding for 6 months or longer included higher maternal IQ (OR, 1.04; 95% CI, 1.01-1.06; *P* = .002), birth weight (OR, 1.68; 95% CI, 1.09-2.59; *P* = .02), and perceived stress (OR, 1.07; 95% CI, 1.02-1.12; *P* = .01). Conversely, maternal smoking history (OR, 0.16; 95% CI, 0.04-0.75; *P* = .02) and prenatal anxiety (OR, 0.87; 95% CI, 0.82-0.94; *P* < .001) were associated with lower odds. Within the WWE subgroup, women with generalized epilepsy (OR, 0.53; 95% CI, 0.31-0.90; *P* = .02) or mixed or unclassified epilepsy (OR, 0.29; 95% CI, 0.10-0.85; *P* = .02) were less likely to reach the 6-month milestone than those with focal epilepsy (eTable 4 in Supplement 1).

### Comparison of Neurodevelopmental Outcomes Between Children of WWE vs HW at Age 6 Years

Among breastfed children, no significant differences were observed between children of WWE and HW across primary outcomes. The adjusted mean (SD) VIS (WWE, 107.93 [0.71]; HW, 107.18 [1.40]; difference, 0.75; 95% CI, −2.40 to 3.89; *P* = .64) and ABAS-3 GAC (WWE, 102.91 [0.75]; HW, 102.14 [1.46]; difference, 0.78; 95% CI, −2.52 to 4.07; *P* = .64) scores were comparable (Figure 1; eTable 5 in Supplement 1). Full multivariable regression models detailing all covariate adjustments are provided in eTable 6 in Supplement 1. Secondary outcomes, including adaptive behavior subdomains (ABAS-3 practical, social, and conceptual subdomains), nonverbal ability, general conceptual ability, and autism risk, also revealed no statistically significant differences in adjusted mean scores between WWE and HW groups (eTables 7-10 in Supplement 1).

**Figure 1.**
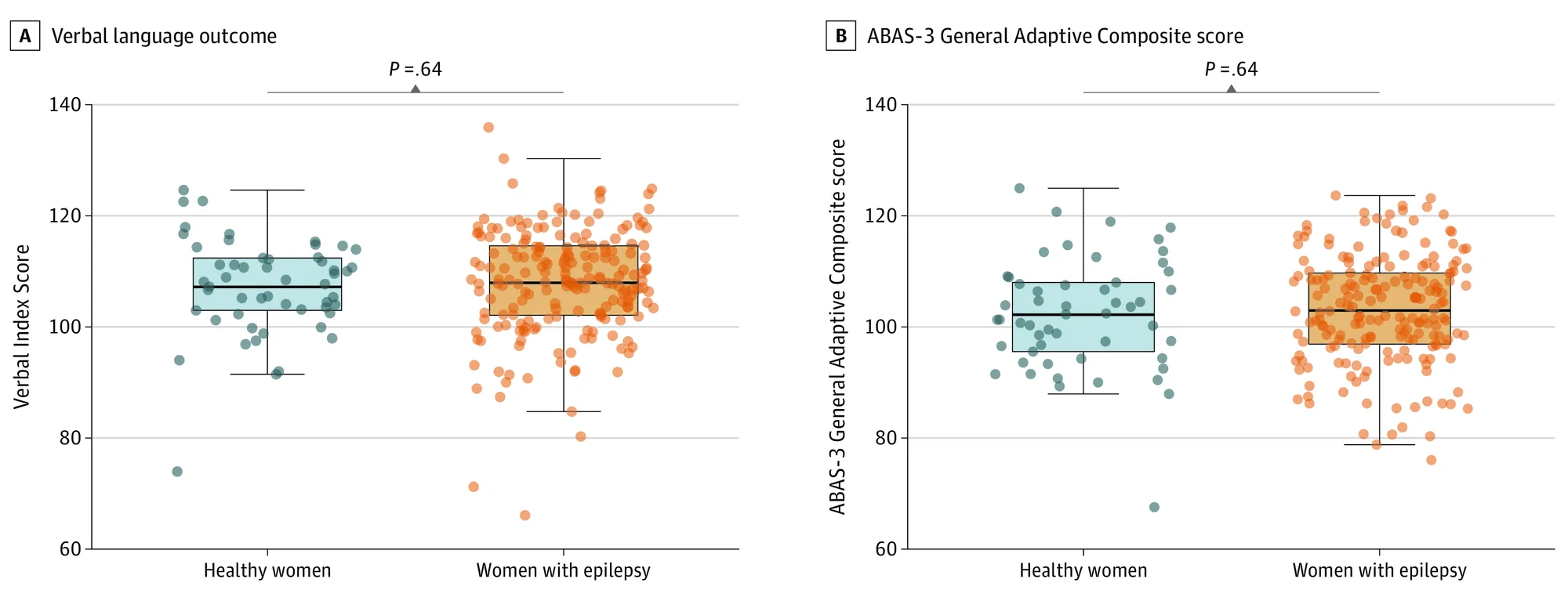
Box-and-Whisker Plots of Verbal Language and Adaptive Behavioral Outcomes at Age 6 Years in Children of Women With Epilepsy vs Healthy Women Following Breastfeeding Exposure A, Adjusted Verbal Index Score individual scores (dots) and box plots for 53 children of healthy women and 192 children of women with epilepsy. Scores were adjusted for breastfeeding duration, mother’s age at enrollment, maternal IQ, prenatal anxiety (Beck Anxiety Inventory score), child sex, and child ethnicity. B, Adjusted Adaptive Behavior Assessment System, Third Edition (ABAS-3) General Adaptive Composite score. Individual scores (dots) and box plots for 53 children of healthy women and 189 children of women with epilepsy. Scores were adjusted for breastfeeding duration, mother’s age at enrollment, birthweight, postpartum stress (Perceived Stress Scale score), child sex, and children’s race and ethnicity. In both panels, the horizontal line within the box represents the mean value, the box boundaries represent the IQR, and the whiskers extend to the minimum and maximum values (excluding outliers). Individual data points are overlaid as a jitter plot to show the distribution of the cohort.

### Association of Breastfeeding Duration With Neurodevelopmental Outcomes in WWE and HW Cohorts

Within the WWE cohort, breastfeeding duration was not significantly associated with verbal language outcomes at age 6 years. However, breastfeeding for 6 months or longer was associated with significantly higher adaptive behavior scores compared with no breastfeeding (estimate, 4.68; 95% CI, 1.34 to 8.03; *P* = .01), whereas shorter breastfeeding duration (0-6 months) did not significantly differ (Table 2; eFigure 2 in Supplement 1). Further analysis of adaptive behavior subdomains revealed that this benefit was driven by improvements in practical (estimate, 5.27; 95% CI, 1.84 to 8.70; *P* = .003) and conceptual(estimate, 3.50; 95% CI, 0.28 - 6.71; *P* = .03) subdomains, with no significant association for the social subdomain (Figure 2; eFigure 3 in Supplement 1). Across all 3 adaptive behavioral subdomains, higher postnatal maternal stress was associated with lower scores (estimates ranging from −0.55 to −0.42) (eTable 11 in Supplement 1).

**Table 2.** Association of Breastfeeding Status With Language and Behavior Outcomes at Age 6 Years Among Children of Women With Epilepsy^a^

Variables	Estimate (95% CI)	*P* value
**Language outcome (Verbal Index Score)**		
Breastfeeding duration, mo		
0	0 [Reference]	NA
0 to <6	0.16 (−3.34 to 3.65)	.93
≥6	0.07 (−3.30 to 3.43)	.97
Maternal IQ	0.32 (0.20 to 0.45)	<.001
Maternal education		
No college degree	0 [Reference]	NA
College degree (not advanced)	3.79 (0.37 to 7.20)	.03
College degree (advanced)	6.62 (2.50 to 10.74)	.002
Child ethnicity		
Hispanic or Latino	0 [Reference]	NA
Non-Hispanic or Non-Latino	3.47 (0.07 to 6.87)	.05
Mean Beck Anxiety Inventory during pregnancy	−0.25 (−0.48 to −0.03)	.03
**Adaptive behavioral outcome (ABAS-3 General Adaptive Composite Score)**		
Breastfeeding duration, mo		
0	0 [Reference]	NA
>0 to <6	2.11 (−1.49 to 5.71)	.25
≥6	4.68 (1.34 to 8.03)	.01
Maternal age	−0.34 (−0.62 to −0.06)	.02
History of depression (yes vs no)	−4.36 (−7.95 to −0.77)	.02
Mean Perceived Stress Scale score post birth^b^	−0.61 (−0.83 to −0.39)	<.001
Children’s race		
White	0 [Reference]	NA
Black	11.20 (4.48 to 17.91)	.001
Others or unknown	0.78 (−2.99 to 4.54)	.68

Abbreviations: ABAS-3, Adaptive Behavior Assessment System, Third Edition; NA, not applicable.

^a^ The final model was derived from a stepwise linear regression algorithm to identify factors significantly associated with the child language or behavior outcome at age 6 years among the cohort of women with epilepsy. Breastfeeding duration—categorized as no breastfeeding, less than 6 months, and 6 months or longer—was included as an a priori covariate and retained in all steps of the modeling process regardless of statistical significance.

^b^ Mean Perceived Stress Scale–14 (PSS) post birth reflects the mean score on all assessments between delivery and the visit at child age 6 years.

**Figure 2.**
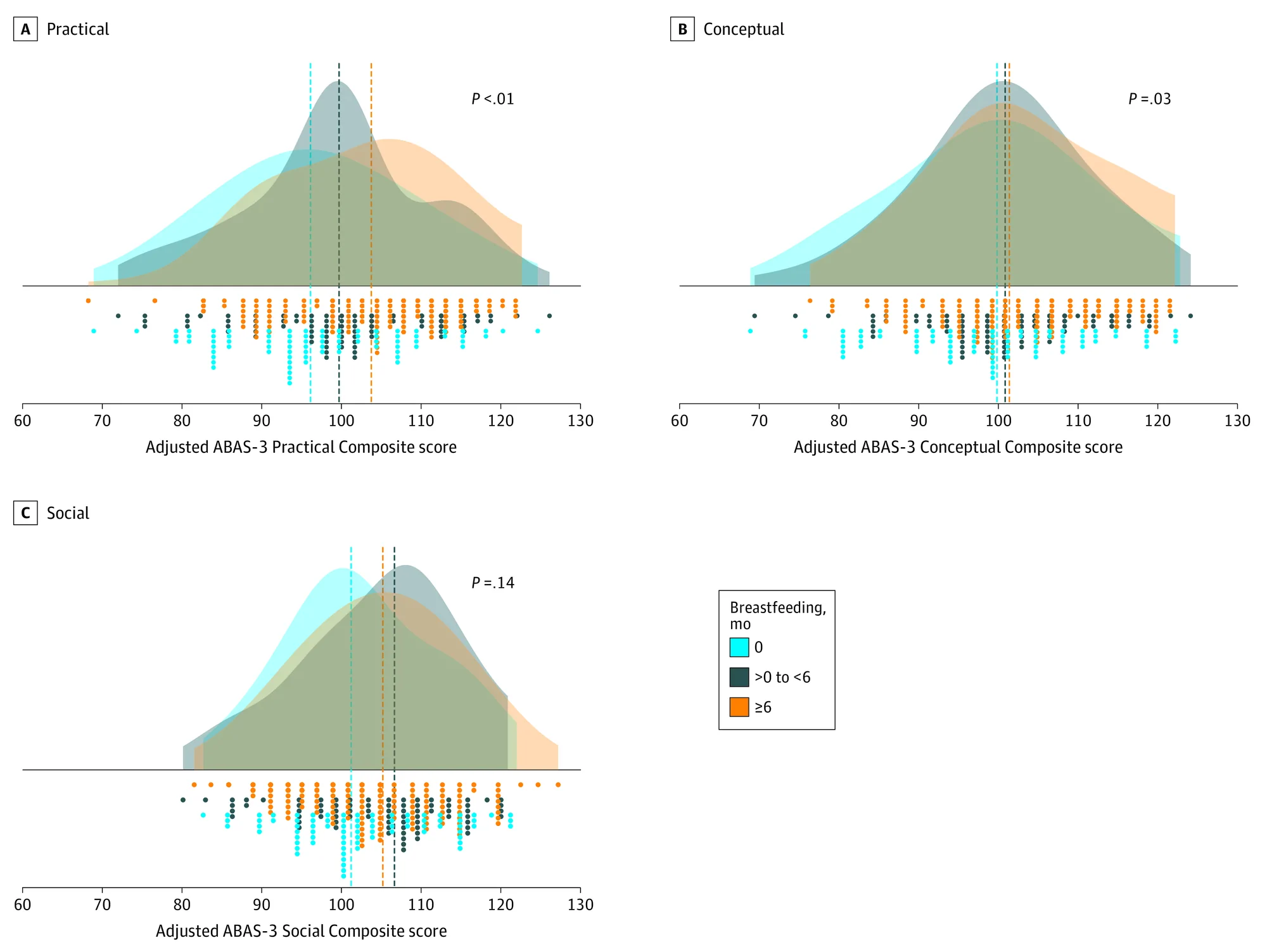
Density Plots of Distribution of Adjusted Adaptive Behavioral Subdomain Scores by Breastfeeding Duration in Children of Women With Epilepsy Raincloud plots display the distribution of adjusted scores for the (A) practical, (B) conceptual, and (C) social subdomains of the Adaptive Behavior Assessment System, Third Edition (ABAS-3) in children of women with epilepsy. Data are stratified by breastfeeding duration: none (light blue), more than 0 to less than 6 months (dark blue), and 6 months or longer (orange). Each panel visualizes the kernel-smoothed probability density (cloud), the exact scatter of individual data points (rain), and the adjusted group median (vertical dashed line). The inclusion of individual data points directly below the density curves allows for the visual assessment of the cohort’s true distribution independent of the selected smoothing parameters. Adjusted scores were derived from multivariable linear regression models controlling for maternal and child covariates (eTable 11 in the Supplement 1). *P* values indicate the significance of the comparison between the group with 6 months or longer breastfeeding durations and the reference group (no breastfeeding). Comprehensive box plots appear in eFigure 3 in Supplement 1.

Regarding other cognitive outcomes, no significant associations were found between breastfeeding duration and nonverbal ability, general conceptual ability, or risk of autism traits (eTables 12-14 in Supplement 1). Tests for interaction between maternal group (WWE vs HW) and extended breastfeeding duration (≥6 months) did not reveal statistically significant evidence that the association between breastfeeding for 6 months or longer and child outcomes differ by maternal epilepsy status (eTable 15 in Supplement 1).

In exploratory analyses among 83 children of HW, the association between extended breastfeeding (≥6 months) and higher VIS scores at age 6 years did not reach statistical significance (estimate, 6.42; 95% CI, −0.02 to 12.86; *P* = .05). Furthermore, there were no statistically significant associations with other neurodevelopmental outcomes, including adaptive behavior (eTables 16-18 and eResults in Supplement 1).

### Neurodevelopmental Outcomes Across ASM Regimens

Analysis of adjusted neurodevelopmental outcomes within specific ASM subgroups (lamotrigine monotherapy, levetiracetam monotherapy, other monotherapy, and polytherapy) revealed no significant differences in language and adaptive behavioral outcomes by breastfeeding duration (Figure 3; eTable 19 in Supplement 1). In the lamotrigine monotherapy subgroup (n = 96), breastfeeding for 6 months or longer was associated with significantly higher adjusted scores across all behavioral subdomains compared with no breastfeeding: practical (estimate, 8.07; 95% CI, 2.29-13.85; *P* = .02), conceptual (estimate, 9.65; 95% CI, 3.55-15.75; *P* = .001), and social (estimate, 5.14; 95% CI, 0.19-10.09; *P* = .04). Similarly, the other monotherapy subgroup showed higher Practical scores with breastfeeding for 6 months or longer (estimate, 12.09; 95% CI, 4.29-19.88; *P* = .01). Nonverbal and general conceptual abilities remained comparable across breastfeeding categories within all medication subgroups (eFigures 4-6 in Supplement 1).

**Figure 3.**
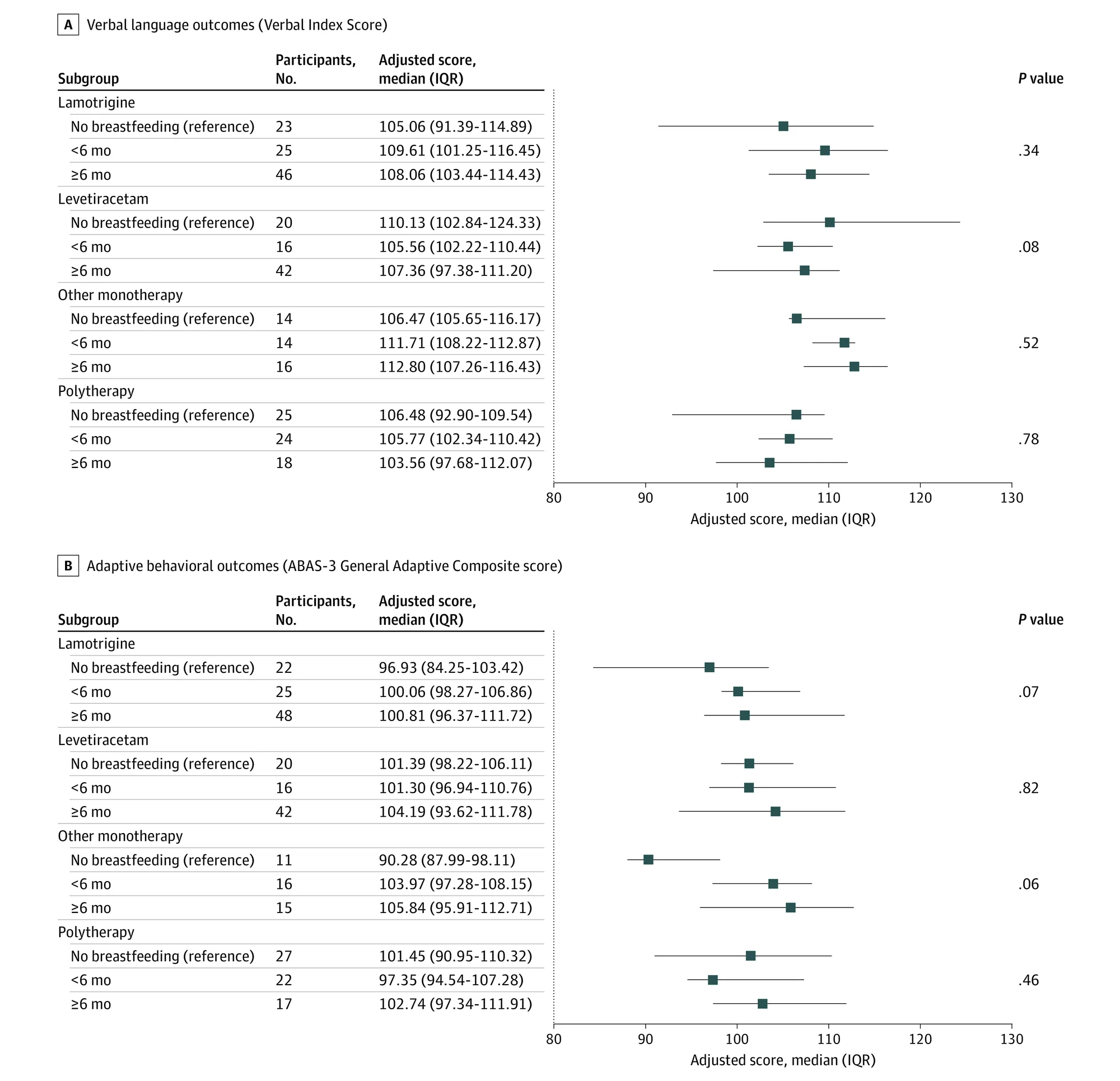
Dot Plots of Association of Breastfeeding Duration Status With Neurodevelopmental Outcomes in Children of Women With Epilepsy at Age 6 Years, Stratified by Antiseizure Medication Regimen Outcomes are stratified by breastfeeding duration: no breastfeeding (reference group), less than 6 months, and 6 months or longer. Data are presented as medians (markers) and IQRs (horizontal lines). A, Verbal language outcomes: adjusted Verbal Index Scores are shown for each medication subgroup. Scores were adjusted for maternal IQ, prenatal anxiety, maternal education, child race and ethnicity, and folate supplementation. B, Adaptive behavioral outcomes: adjusted Adaptive Behavior Assessment System, Third Edition (ABAS-3) General Adaptive Composite scores are shown for each medication subgroup. Scores were adjusted for maternal age, history of depression, postpartum stress, and birth weight. *P* values represent significance across the 3 breastfeeding groups, calculated using the Kruskal-Wallis test.

Among aggregated monotherapy-exposed children (n = 212), those breastfed for 6 months or longer had significantly higher adaptive behavioral scores than those not breastfed (mean [SD], 103.34 [10.37] vs 97.46 [11.48]; adjusted difference, 5.88; 95% CI, 2.21 to 9.55; *P* = .001), while breastfeeding for less than 6 months yielded no significant difference. No significant associations were observed for polytherapy-exposed children (for ≥6 months vs no breastfeeding: adjusted difference, 3.02; 95% CI, −3.56 to 9.60; *P* = .39; for <6 months vs no breastfeeding: adjusted difference, −0.68; 95% CI, −6.51 to 5.16; *P* = .83) (eTable 20 in Supplement 1). Verbal language, nonverbal ability, and general conceptual ability did not differ by breastfeeding duration in either monotherapy or polytherapy cohorts (eFigures 7-10 and eTable 20 in Supplement 1). Autism risk assessment was limited by low incidence and therefore inconclusive (8 of 245 monotherapy-exposed [3.3%]; 1 of 34 polytherapy-exposed [2.9%]) (eTable 21 in Supplement 1). Furthermore, all sensitivity analyses yielded results consistent with the primary findings (eTables 22-24 in Supplement 1).

## Discussion

This prospective, multicenter study found that neurodevelopmental outcomes at age 6 years are comparable between children of WWE taking contemporary ASMs—primarily levetiracetam and lamotrigine—and children of HW following breastfeeding exposure, regardless of breastfeeding duration. These findings provide evidence that breastfeeding does not pose neurodevelopmental risk for children of WWE using contemporary ASM regimens. Furthermore, breastfeeding for 6 months or longer was associated with improved adaptive behavioral outcomes in children of WWE, whereas shorter durations showed no significant benefit, suggesting a duration-dependent threshold for this association.

Our analysis found that all measured neurodevelopmental trajectories—including verbal language, adaptive behavior, nonverbal ability, general conceptual ability, and autism risk—were comparable between children of WWE and those of HW who breastfed, regardless of duration. These results provide evidence to reassure clinicians and patients that breastfeeding while taking commonly prescribed contemporary ASMs is not associated with adverse neurodevelopmental trajectories of their children. These findings align with and extend historical data. Existing literature supports the safety of breastfeeding while taking older-generation ASMs, such as valproate, carbamazepine, and phenytoin, which showed no adverse effects at age 3 years^30^ and was associated with higher verbal abilities by age 6 years compared with nonbreastfed children.^18^ Research on newer-generation ASMs corroborates these findings at earlier developmental milestones. Infant exposure to levetiracetam or lamotrigine through breast milk was not associated with adverse outcomes at age 2 years in a European cohort,^31^ and our previous investigations into lamotrigine exposure showed similar cognitive stability.^18^ Furthermore, pharmacokinetic data from the present cohort indicate that approximately 49% of ASM concentrations in nursing infants were below the lower limit of quantification, suggesting that breast milk represents a substantially lower level of drug exposure than in utero transfer.^32^ Collectively, these data suggest that the benefits of breastfeeding likely outweigh the risks associated with ASM exposure in early childhood. Translating these findings into practice, our data provide actionable counseling points to further promote the higher breastfeeding rates typically achieved at epilepsy specialty centers. Integrating these evidence-based reassurances into routine preconception and postpartum care has the potential to increase sustained breastfeeding rates across the broader WWE population.

In children of WWE taking ASMs, most neurodevelopmental outcomes were comparable across different breastfeeding status. Notably, breastfeeding for 6 months or longer demonstrated a significant positive association with adaptive behavioral outcomes at age 6 years, with benefits observed across multiple subdomains. A key finding is that these benefits were not observed for shorter breastfeeding durations (0-6 months), indicating a minimum duration threshold may be needed to achieve these adaptive behavioral benefits. It is known that the benefits of breastfeeding extend beyond nutrition, offering numerous advantages.^9^ Multiple international guidelines hence recommend exclusive breastfeeding for at least the first 6 months of life.^6,7,8,28,29^ In the general population, longer breastfeeding duration correlates with superior neurodevelopmental trajectories, and reduced risks of attention-deficit/hyperactivity disorder and autism spectrum disorder.^33,34,35,36,37,38^ Breastfeeding for 6 months or longer has been associated with more favorable behavioral outcomes at age 5 years,^37^ and improved academic performance in adolescence.^38^ Our findings demonstrated that these advantages of behavioral outcomes persist in children of WWE despite ASM exposure, highlighting the 6-month breastfeeding milestone as an important target for optimizing long-term adaptive functioning in this population.

Analysis of ASM subgroups revealed no significant differences in primary neurodevelopmental outcomes between levetiracetam, lamotrigine, other monotherapy, and polytherapy stratified by breastfeeding duration. Secondary analysis demonstrated that breastfeeding for 6 months or longer in the lamotrigine monotherapy subgroup was associated with significantly higher scores across all adaptive behavioral subdomains (conceptual, social, and practical functioning). These findings are particularly relevant to modern clinical practice. Over the last 2 decades, lamotrigine and levetiracetam have become the most commonly prescribed ASMs for WWE globally due to their favorable safety profiles.^17,39,40^ Our cohort reflects this shift and aligns with current guidelines recommending these agents as first-line therapies for WWE of childbearing age.^41^ Notably, while prior research suggested lamotrigine was associated with lower breastfeeding rates compared with levetiracetam,^10^ our data indicate that breastfeeding for 6 months or longer was not dependent on ASM type. Instead, extended breastfeeding was influenced by modifiable factors, such as maternal anxiety and smoking history, which should be prioritized during clinical counseling. The low risk of breastfeeding while taking these agents is further supported by their pharmacokinetic profiles. Although levetiracetam partitions extensively into breast milk (75%-155% of maternal plasma),^42,43^ infant serum levels remain relatively low (approximately 5.2% of maternal levels), likely due to high renal clearance in infants.^32^ Conversely, the milk-to-serum ratio for lamotrigine is moderate (18%-74%),^44^ and infant-to-maternal serum ratios are approximately 28.9%, with greater variability than levetiracetam.^32^ Despite the slightly higher infant ASM exposure ratio of lamotrigine, our data demonstrated that breastfeeding—particularly when exceeding the 6-month threshold—is associated with significant improved neurodevelopmental benefits.

When ASMs were stratified into monotherapy and polytherapy cohorts, the monotherapy group showed significant adaptive behavioral improvements, consistent with the overall WWE cohort. Importantly, children exposed to polytherapy exhibited no significant differences in cognitive or behavioral outcomes regardless of breastfeeding duration. Therefore, polytherapy should not be considered a contraindication to breastfeeding. Ultimately, the broad advantages of breastfeeding established for the children—including reduced risks of respiratory infections, sudden infant death syndrome, type 2 diabetes, obesity, and atopic dermatitis^9,33,45^—combined with the maternal benefits of reduced postpartum depression, cardiovascular disease, and breast and ovarian cancers,^9,28,33,46^ should outweigh the concerns of ASM exposure. Our findings reinforce the recommendation that WWE, including those taking contemporary ASMs or polytherapy, should be supported in their decision to breastfeed given the multiple benefits and lack of risk. Furthermore, breastfeeding for at least 6 months may maximize long-term adaptive behavioral outcomes.

### Limitations

Our study has several limitations. First, despite the prospective design and adjustment for numerous confounders, the possibility of residual confounding from unmeasured variables cannot be excluded. For example, breastfeeding duration might reflect unmeasured caregiving or environmental factors influencing neurodevelopment. Second, this investigation serves as a secondary analysis of the longitudinal MONEAD cohort, it was not a priori powered for further fine-grained stratifications. While the primary comparisons regarding breastfeeding duration retain adequate statistical power in the whole cohort, further stratification into specific ASM regimens results in small sample sizes. Consequently, subgroup analyses evaluating specific ASM types, especially less common monotherapies or polytherapy are likely underpowered to draw definitive conclusions and should be interpreted with caution. Future research is required to further evaluate these specific ASM medications. Third, while our cohort reflects real-world feeding practices among WWE, the lack of data on breastfeeding exclusivity limits our ability to further assess a potential dose-response association between ASM exposure and neurodevelopmental outcomes. Finally, because recruitment occurred primarily at specialized tertiary epilepsy centers, the cohort may reflect a population with more complex epilepsy or different demographic characteristics than those managed in general or rural community practices. This distinction, particularly regarding the use of older-generation ASMs, should be considered when generalizing these findings.

## Conclusions

In summary, this study provides comprehensive evidence supporting the long-term neurodevelopmental safety of breastfeeding for WWE taking contemporary ASMs. We found that infant ASM exposure through breastfeeding was not associated with adverse cognitive or behavioral outcomes at age 6 years. Importantly, breastfeeding for 6 months or longer was associated with a significant benefit to behavioral outcome. These results reinforce the recommendation that WWE should be supported in their decision to breastfeed for at least 6 months to maximize long-term outcomes for their children.

## Notes

Supplement 1.
eMethods.

eResults.
**eFigure 1.** Flow Chart of Patient Recruitment **eFigure 2.** Adjusted Language and Adaptive Behavioral Outcomes at Age 6 Years in Children of Women With Epilepsy Stratified by Breastfeeding Duration **eFigure 3.** Unadjusted Distributions of Adaptive Behavioral Subdomain Outcomes at Age 6 Years in Children of Women With Epilepsy Stratified by Breastfeeding Duration **eFigure 4.** Adaptive Behavioral Subdomain Outcomes at Age 6 Years by Breastfeeding Duration and Antiseizure Medication Regimens **eFigure 5.** Nonverbal Cognitive Outcomes at Age 6 Years by Breastfeeding Duration and Antiseizure Medication Regimens **eFigure 6.** General Conceptual Ability Outcomes at Age 6 Years by Breastfeeding Duration and Antiseizure Medication Regimens **eFigure 7.** Neurodevelopmental Outcomes at Age 6 Years by Breastfeeding Duration and Antiseizure Medication Regimen: Monotherapy vs Polytherapy **eFigure 8.** Adaptive Behavioral Subdomain Outcomes at Age 6 Years by Breastfeeding Duration and Antiseizure Medication Type: Monotherapy vs Polytherapy **eFigure 9.** Nonverbal Cognitive Outcomes at Age 6 Years by Breastfeeding Duration and Antiseizure and Antiseizure Medication Type: Monotherapy vs Polytherapy **eFigure 10.** General Conceptual Ability Outcomes at Age 6 Years by Breastfeeding Duration and Antiseizure and Antiseizure Medication Type: Monotherapy vs Polytherapy **eTable 1.** Baseline Demographic and Clinical Characteristics of Language Outcome Analysis: Women With Epilepsy vs Healthy Women **eTable 2.** Baseline Demographic and Clinical Characteristics of Adaptive Behavior Analysis: Women With Epilepsy vs Healthy Women **eTable 3.** Multivariable Logistic Regression Model of Factors Associated With Breastfeeding (≥6 Months) in the Whole Cohort (Women With Epilepsy and Healthy Women) **eTable 4.** Multivariable Logistic Regression Model of Factors Associated With Breastfeeding (≥6 Months) Among Women With Epilepsy **eTable 5.** Language and Behavior Outcome in Children of Women With Epilepsy vs Healthy Controls Following Breastfeeding Exposure **eTable 6.** Multivariable Regression Models Comparing Verbal Index Scores and Adaptive Behavioral Outcomes at Age 6 Years Between Children of Women With Epilepsy and Healthy Women **eTable 7.** Multivariable Regression Models Comparing Adaptive Behavioral Subdomain Outcomes at Age 6 Years Between Children of Women With Epilepsy and Healthy Women **eTable 8.** Multivariable Regression Models Comparing Nonverbal Ability at Age 6 Years Between Children of Women With Epilepsy and Healthy Women **eTable 9.** Multivariable Regression Models Comparing General Conceptual Ability at Age 6 Years Between Children of Women With Epilepsy and Healthy Women **eTable 10.** Multivariable Regression Models Comparing Autism Risk Outcomes at Age 6 Years Between Children of Women With Epilepsy and Healthy Women **eTable 11.** Association Between Breastfeeding Duration and Adaptive Behavior Subdomain Outcomes at Age 6 Years in Children Born to Women With Epilepsy **eTable 12.** Association Between Breastfeeding Duration and Nonverbal Ability Outcomes at Age 6 Years in Children Born to Women With Epilepsy **eTable 13.** Association Between Breastfeeding Duration and General Cognitive Ability at Age 6 Years in Children Born to Women With Epilepsy at Age 6 Years **eTable 14.** Association Between Breastfeeding Duration and Risk of Autism Traits at Age 6 Years in Children Born to Women With Epilepsy **eTable 15.** Analysis of the Interaction Between Maternal Epilepsy Status and Breastfeeding Duration on Neurodevelopmental Outcomes at Age 6 Years in Children Born to Women With Epilepsy **eTable 16.** Association of Breastfeeding Duration With Child Language and Behavioral Outcomes at Age 6 Years in Children of Healthy Women **eTable 17.** Association of Breastfeeding Duration With Child Nonverbal Ability, General Cognitive Ability, and Behavioral Subdomains at Age 6 Years in Children of Healthy Women **eTable 18.** Rate of Autism Spectrum at Risk at Age 6 Years by Breastfeeding Duration in Children of Healthy Women **eTable 19.** Distribution of Antiseizure Medication Use Regimens in Women With Epilepsy **eTable 20.** Neurodevelopmental Outcomes at Age 6 Years in Children of WWE Stratified by Antiseizure Medication (Monotherapy vs Polytherapy) and Breastfeeding Duration **eTable 21.** Association Between Breastfeeding Duration and Autism Risk at Age 6 Years in Children of Women With Epilepsy, Stratified by Antiseizure Medication Regimen or Types **eTable 22.** Sensitivity Analysis of the Association Between Breastfeeding Duration and Verbal Index Score at Age 6 Years Using AIC-Optimized Stepwise Regression **eTable 23.** Sensitivity Analysis of the Association Between Breastfeeding Duration and Adaptive Behavior at Age 6 Years Using AIC-Optimized Stepwise Regression **eTable 24.** Sensitivity Analysis of Primary Outcomes Using Propensity Score Approach **eAppendix.** Trial Protocol and Statistical Analysis Plan

Supplement 2.
Nonauthor Collaborators

Supplement 3.
Data Sharing Statement
