# Supplementary material for: Breastfeeding and Neurodevelopmental Outcomes in Children of Women Taking Antiseizure Medications
Source: JAMA Netw Open. 2026 Sep 30;9(9):e2637040. doi: 10.1001/jamanetworkopen.2026.37040 (PMC13628311; doi:10.1001/jamanetworkopen.2026.37040)
Supplement: Supplementary file 1 [file jamanetwopen-e2637040-s001.pdf]

Supplemental Online Content

Li Y, Loring DW, Cohen MJ, et al; MONEAD Investigator Group. Breastfeeding and neurodevelopmental outcomes in children of women taking antiseizure medications. *JAMA Netw Open.* 2026;9(9):e2637040. doi:10.1001/jamanetworkopen.2026.37040

eMethods.

eResults.

eFigure 1. Flow Chart of Patient Recruitment

eFigure 2. Adjusted Language and Adaptive Behavioral Outcomes at Age 6 Years in Children of Women With Epilepsy Stratified by Breastfeeding Duration

eFigure 3. Unadjusted Distributions of Adaptive Behavioral Subdomain Outcomes at Age 6 Years in Children of Women With Epilepsy Stratified by Breastfeeding Duration

eFigure 4. Adaptive Behavioral Subdomain Outcomes at Age 6 Years by Breastfeeding Duration and Antiseizure Medication Regimens

eFigure 5. Nonverbal Cognitive Outcomes at Age 6 Years by Breastfeeding Duration and Antiseizure Medication Regimens

eFigure 6. General Conceptual Ability Outcomes at Age 6 Years by Breastfeeding Duration and Antiseizure Medication Regimens

eFigure 7. Neurodevelopmental Outcomes at Age 6 Years by Breastfeeding Duration and Antiseizure Medication Regimen: Monotherapy vs Polytherapy

eFigure 8. Adaptive Behavioral Subdomain Outcomes at Age 6 Years by Breastfeeding Duration and Antiseizure Medication Type: Monotherapy vs Polytherapy

eFigure 9. Nonverbal Cognitive Outcomes at Age 6 Years by Breastfeeding Duration and Antiseizure and Antiseizure Medication Type: Monotherapy vs Polytherapy

eFigure 10. General Conceptual Ability Outcomes at Age 6 Years by Breastfeeding Duration and Antiseizure and Antiseizure Medication Type: Monotherapy vs Polytherapy

eTable 1. Baseline Demographic and Clinical Characteristics of Language Outcome Analysis: Women With Epilepsy vs Healthy Women

eTable 2. Baseline Demographic and Clinical Characteristics of Adaptive Behavior Analysis: Women With Epilepsy vs Healthy Women

eTable 3. Multivariable Logistic Regression Model of Factors Associated With Breastfeeding (≥6 Months) in the Whole Cohort (Women With Epilepsy and Healthy Women)

eTable 4. Multivariable Logistic Regression Model of Factors Associated With Breastfeeding (≥6 Months) Among Women With Epilepsy

eTable 5. Language and Behavior Outcome in Children of Women With Epilepsy vs Healthy Controls Following Breastfeeding Exposure

eTable 6. Multivariable Regression Models Comparing Verbal Index Scores and Adaptive Behavioral Outcomes at Age 6 Years Between Children of Women With Epilepsy and Healthy

© 2026 Li Y et al. *JAMA Network Open*.

Women

eTable 7. Multivariable Regression Models Comparing Adaptive Behavioral Subdomain Outcomes at Age 6 Years Between Children of Women With Epilepsy and Healthy Women

eTable 8. Multivariable Regression Models Comparing Nonverbal Ability at Age 6 Years Between Children of Women With Epilepsy and Healthy Women

eTable 9. Multivariable Regression Models Comparing General Conceptual Ability at Age 6 Years Between Children of Women With Epilepsy and Healthy Women

eTable 10. Multivariable Regression Models Comparing Autism Risk Outcomes at Age 6 Years Between Children of Women With Epilepsy and Healthy Women

eTable 11. Association Between Breastfeeding Duration and Adaptive Behavior Subdomain Outcomes at Age 6 Years in Children Born to Women With Epilepsy

eTable 12. Association Between Breastfeeding Duration and Nonverbal Ability Outcomes at Age 6 Years in Children Born to Women With Epilepsy

eTable 13. Association Between Breastfeeding Duration and General Cognitive Ability at Age 6 Years in Children Born to Women With Epilepsy at Age 6 Years

eTable 14. Association Between Breastfeeding Duration and Risk of Autism Traits at Age 6 Years in Children Born to Women With Epilepsy

eTable 15. Analysis of the Interaction Between Maternal Epilepsy Status and Breastfeeding Duration on Neurodevelopmental Outcomes at Age 6 Years in Children Born to Women With Epilepsy

eTable 16. Association of Breastfeeding Duration With Child Language and Behavioral Outcomes at Age 6 Years in Children of Healthy Women

eTable 17. Association of Breastfeeding Duration With Child Nonverbal Ability, General Cognitive Ability, and Behavioral Subdomains at Age 6 Years in Children of Healthy Women

eTable 18. Rate of Autism Spectrum at Risk at Age 6 Years by Breastfeeding Duration in Children of Healthy Women

eTable 19. Distribution of Antiseizure Medication Use Regimens in Women With Epilepsy

eTable 20. Neurodevelopmental Outcomes at Age 6 Years in Children of WWE Stratified by Antiseizure Medication (Monotherapy vs Polytherapy) and Breastfeeding Duration

eTable 21. Association Between Breastfeeding Duration and Autism Risk at Age 6 Years in Children of Women With Epilepsy, Stratified by Antiseizure Medication Regimen or Types

eTable 22. Sensitivity Analysis of the Association Between Breastfeeding Duration and Verbal Index Score at Age 6 Years Using AIC-Optimized Stepwise Regression

eTable 23. Sensitivity Analysis of the Association Between Breastfeeding Duration and Adaptive Behavior at Age 6 Years Using AIC-Optimized Stepwise Regression

eTable 24. Sensitivity Analysis of Primary Outcomes Using Propensity Score Approach

eAppendix. Trial Protocol and Statistical Analysis Plan

This supplemental material has been provided by the authors to give readers additional information about their work.

© 2026 Li Y et al. *JAMA Network Open*.

eMethods.

This document provides additional details regarding the materials and methods, sensitivity results, as well as eTables and eFigures results in the main manuscript.

1. Detailed Participant Inclusion and Exclusion Criteria

1) Inclusion Criteria for Women

- **Applicable to All Women:**
  - Age 14-45, inclusive.
  - Diagnosis of epilepsy (for WWE groups) or healthy status (for HW group).
  - Ability to maintain a daily medical diary.
  - Language skills in English or Spanish adequate to perform study tasks.
  - Access to a telephone for necessary contacts.
  - Gestational age of 20 weeks or less.
  - Ability to commit to follow-up for 6 years post-delivery.

2) Exclusion Criteria for Women

- **Applicable to All Women:**
  - Expected IQ < 70.
  - History of psychogenic non-epileptic spells (PNES).
  - Progressive cerebral disease (e.g., multiple sclerosis, brain tumor).
  - Presence of other major medical illness (e.g., diabetes, cancer).
  - IV drug use in the past year; or alcohol abuse, cocaine, or methamphetamine use since the beginning of pregnancy.
  - Positive test history for Syphilis or HIV.
  - Concurrent participation in an experimental drug trial.
  - Any condition that, in the investigator's judgment, is a contraindication to participation or impairs the ability to give informed consent.
  - Exposure to known teratogens during the current pregnancy (excluding ASMs).
  - Detection of a fetal major congenital malformation prior to enrollment.
  - Known genetic disorder in herself or a primary relative (exceptions considered on a case-by-case basis).
  - Primary use of a non-licensed midwife or planning a home delivery independent from a hospital.
- **Applicable to All Women with Epilepsy:**
  - Planned surgical intervention for epilepsy during the study period.
  - History of switching ASMs during the current pregnancy *prior* to enrollment. "Switching" includes initiating, discontinuing, or changing ASM therapy.

2. Sensitivity Analyses Methods:

To evaluate the robustness of the primary findings and address potential sources of bias, 3 prespecified sensitivity analyses were conducted:

1) Alternative Exposure Definition: Breastfeeding duration was re-evaluated as a binary exposure, defined as extended duration (≥6 months) versus shorter duration or no breastfeeding (<6 months). This threshold aligns with WHO public health recommendations.

© 2026 Li Y et al. *JAMA Network Open*.

2) Maternal Group Interaction Analysis: To formally test whether the association between breastfeeding and child neurodevelopment was modified by maternal epilepsy or antiseizure medication (ASM) exposure, an interaction term (maternal group × breastfeeding status) was incorporated into the multivariable models using the entire study sample.

3) Independence of Data (Multiple Births): To account for the nonindependence of data from twin or multiple-birth sets, primary analyses were repeated after randomly selecting 1 child from each set.

4) To mitigate the risk of overfitting and validate the variable selection process, and maximize overall model fit, a sensitivity analysis was conducted for the primary outcomes (VIS and ABAS-GAC score at 6 years old). Candidate variables were first checked for multicollinearity, with a Variance Inflation Factor < 4 required for inclusion. A stepwise regression algorithm was then applied, optimizing strictly for the lowest Akaike Information Criterion (AIC) rather than relying on *p*-value thresholds. The base null model forced the retention of the primary exposures of interest (breastfeeding duration) to ensure their inclusion in all evaluated models.

5) To rigorously account for baseline covariate differences between exposure groups, we conducted a sensitivity analysis utilizing Inverse Probability of Treatment Weighting (IPTW) combined with standardization (the G-formula). We computed stabilized IPTW weights using logistic regression to balance a comprehensive list of baseline maternal and child characteristics across the cohorts. Subsequently, a generalized linear model was fit using IPTW-weighted maximum likelihood estimation, and standardization was applied to compute the average treatment effect. This doubly robust methodology ensures unbiased estimates provided that either the propensity score weight model or the outcome regression model is correctly specified. We selected this approach over traditional 1:1 propensity score matching or quintile stratification because it allows us to utilize the full analytic cohort and maximize statistical power without truncating the sample size or risking residual confounding within small strata.

© 2026 Li Y et al. *JAMA Network Open*.

eResults.

The results of the sensitivity analyses confirmed the stability of the primary outcomes:

1) Binary Threshold Analysis: When analyzed as a binary variable, breastfeeding for 6 months or longer remained significantly associated with higher adaptive behavior (ABAS-3 GAC) scores compared with the less than 6 months group (P = .01). This benefit was consistently driven by improvements in the Practical (P < .01) and Conceptual (P = .03) subdomains. This is consistent with the conclusion from the main analysis.

2) Maternal Group Interaction: No significant interactions were found between maternal group (women with epilepsy vs healthy women) and extended breastfeeding duration for any primary or secondary neurodevelopmental outcome. All interaction P values exceeded .05, indicating that the benefits of breastfeeding on child development are not significantly altered by maternal epilepsy status or the presence of ASMs (eTable 15 in Supplement 3).

3) Multiple Births Analysis: We identified 14 pairs of twins born to women with epilepsy and 4 pairs born to healthy women. One child was randomly selected from each twin pair and retained in the analyses. The primary outcome analyses revealed: (1) Primary neurodevelopmental outcomes at age 6 years: There were no significant differences between children of WWE and HW. The adjusted mean (SD) Verbal Index Score (VIS) was 106.97 (11.96) for WWE and 108.47 (11.19) for HW (P = .33). Similarly, the adjusted ABAS-3 GAC score showed no significant group differences (WWE: 101.96 [11.61] vs HW: 102.60 [11.35]; P = .66). (2) Association between extended breastfeeding and improved adaptive behavior: Extended breastfeeding remained significantly associated with improved adaptive behavior in this restricted cohort (estimate = 4.12; 95% CI, 0.59-7.64; P = .02). These findings confirm that the primary study results are robust and not driven by the correlation between siblings in multiple births.

4) A sensitivity analysis utilizing regression model optimized for AIC (VIF < 4 for all candidate variables) yielded optimal models that were highly consistent with the primary p-value-based analyses. In the AIC-optimized models, breastfeeding for 6 months or longer remained significantly associated with higher ABAS-3 GAC scores compared to no breastfeeding (Estimate: 4.45, 95% CI: 0.86–8.04; p=0.015), while associations with the VIS outcome remained non-significant, confirming the stability of the primary findings (eTable 22-23)

5) A sensitivity analysis using proposent score approach yielded findings consistent with our primary multivariable regression models. In the primary cohort comparison, after standardizing baseline factors, there remained no significant differences between children of women with epilepsy (WWE) and healthy women (HW) regarding language outcomes (Verbal Index Score [VIS] estimate, 0.12; 95% CI, -2.55 to 2.79; *P* = .93) or adaptive behavior (ABAS-3 GAC estimate, 1.18; 95% CI, -1.76 to 4.12; *P* = .43). Furthermore, within the WWE cohort, the duration-dependent benefit of breastfeeding was fully preserved. Children breastfed for ≥ 6 months maintained a statistically significant advantage in adaptive behavioral outcomes compared to those who received no breast milk (Estimate, 3.60; 95% CI, 0.27 to 6.93; *P* = .03). Language outcomes remained comparable across breastfeeding duration groups (Estimate, 0.19; 95% CI, -3.39 to 3.77; *P* = .92). These results confirm that the associations observed in the primary analyses are highly robust to potential baseline confounding. (eTable 24).

4. Minimum Detectable Effect Size (MDES) Analysis

To confirm the study retained adequate statistical power despite specific cohort stratifications and different adjustment variables, we calculated the Minimum Detectable Effect Size (MDES) based on the final study cohort sample size for both primary outcomes (Verbal Index Score [VIS] and ABAS-3 GAC). Calculations assumed standard parameters (SD = 15; α = .05; power = 80%). For the main cohort comparisons, the study is powered to detect differences well below the 0.5 SD threshold targeted in the original MONEAD protocol. Specifically, the MDES is 0.36 SD (5.39 points) for

© 2026 Li Y et al. *JAMA Network Open*.

the VIS comparison (261 WWE vs. 80 HW) and 0.35 SD (5.31 points) for the ABAS-3 GAC comparison (261 WWE vs. 83 HW).

When evaluating the primary exposure of interest within the WWE cohort, the study retains adequate power to detect clinically meaningful differences across the breastfeeding duration tiers. For the VIS, the MDES is 0.42 SD (6.28 points) for the comparison of ≥ 6 months versus none, and 0.46 SD (6.96 points) for the comparison of < 6 months versus none. Similarly, for the ABAS-3 GAC, the MDES is 0.43 SD (6.39 points) for ≥ 6 months versus none, and 0.48 SD (7.14 points) for < 6 months versus none. All calculated MDES values fall below the originally targeted 0.5 SD threshold, indicating that the study is adequately powered for these specific stratifications and endpoints.

5. Exploratory Analysis Result:

Association of Breastfeeding Duration with Child Neurodevelopmental Outcomes Among Healthy Women
In exploratory analyses among children born to healthy women (n=83), we examined the association between breastfeeding duration and neurodevelopmental outcomes at age 6 years, including verbal language abilities, general cognitive functioning, adaptive behavior, and autism spectrum disorder (ASD) risk. Breastfeeding duration was categorized as no breastfeeding (reference group, n=11), >0 to <6 months (n=15), and ≥6 months (n=57).

For the primary verbal language outcome (Verbal Index Score), we observed no significant association with breastfeeding duration ≥6 months compared with no breastfeeding (adjusted coefficient 6.42; 95% CI, −0.02 to 12.86; P = .05), after adjustment for maternal IQ, maternal age, child sex, and average folate supplementation during the first trimester (eTable 16). However, breastfeeding duration <6 months showed no association (adjusted coefficient 0.65; 95% CI, −7.03 to 8.34; P = .87). This trend toward significance should be interpreted with caution given the exploratory nature of this analysis, small sample size in the no breastfeeding group.

For the primary adaptive behavioral outcome (ABAS-3 General Adaptive Composite Score), no significant associations were observed for either breastfeeding duration category after adjustment for child race. The adjusted coefficients were −3.47 (95% CI, −12.06 to 5.11; P = .42) for >0 to <6 months and −0.15 (95% CI, −7.26 to 6.97; P = .97) for ≥6 months, compared with no breastfeeding (eTable 16).

Secondary analyses examined nonverbal reasoning ability, general conceptual ability, and adaptive behavioral subdomains (eTable 17). No significant associations were found for any of these outcomes. The overall prevalence of ASD risk (SRS-2 T-score ≥70) was low, at 3.6% (3 of 83 children). The distribution of cases across breastfeeding categories was 9.1% (1 of 11) in the no-breastfeeding group, 0% (0 of 16) in the >0 to <6 months group, and 3.5% (2 of 57) in the ≥6 months group (eTable 18). Due to the low event rate, the cohort lacked sufficient statistical power to perform meaningful multivariable logistic regression analysis for this outcome.

These exploratory analyses in a healthy cohort suggest a potential positive trend for verbal abilities associated with breastfeeding duration ≥6 months, though this did not reach conventional statistical significance. Given the primary study's focus on women with epilepsy treated with antiseizure medications, these supplementary findings in healthy women should be considered hypothesis-generating. The limited statistical power, particularly the small size of the no-breastfeeding reference group, necessitates further investigation in larger prospective cohorts for healthy women population

© 2026 Li Y et al. *JAMA Network Open*.

eFigure 1. Flow Chart of Patient Recruitment

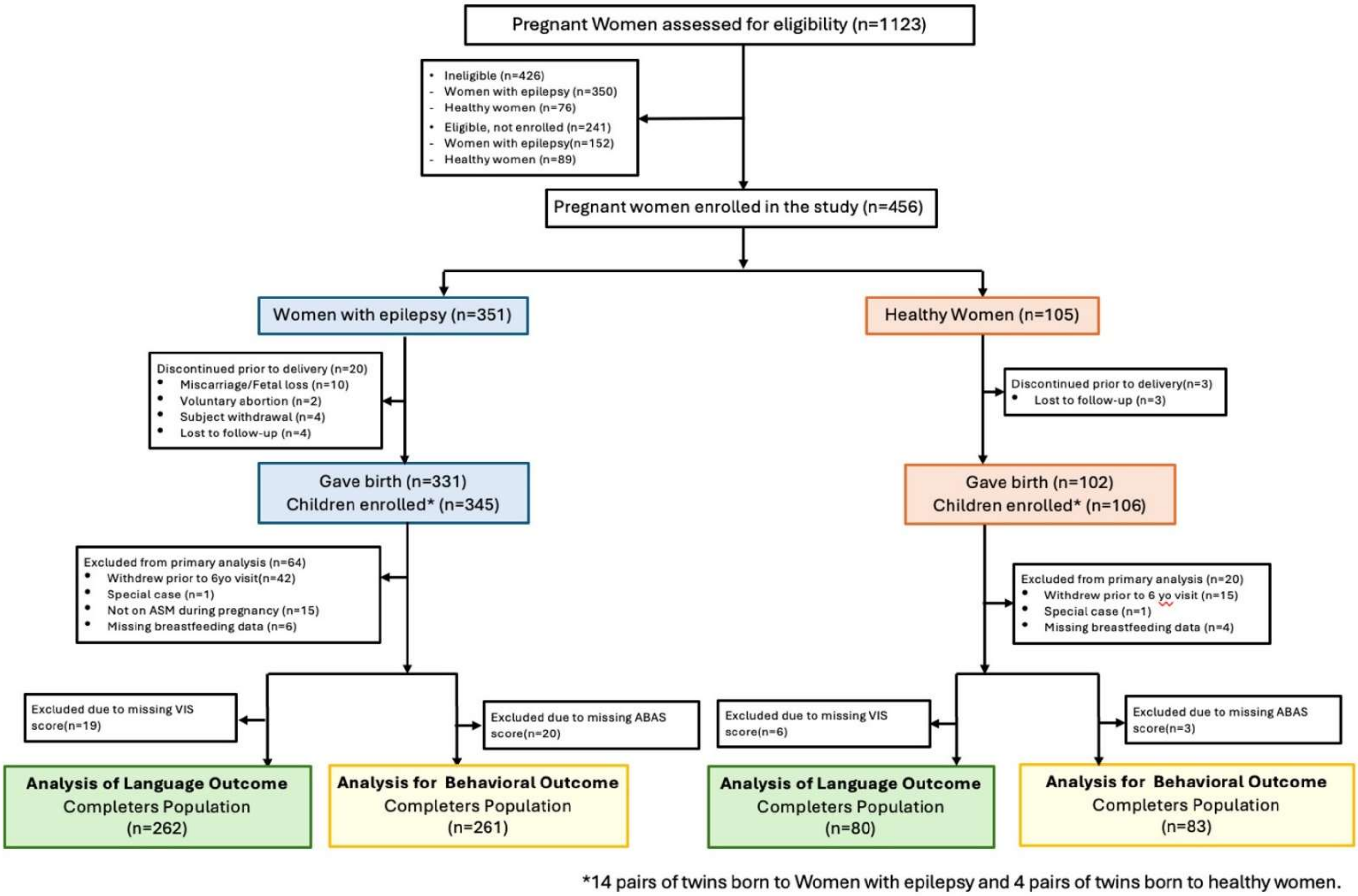

© 2026 Li Y et al. *JAMA Network Open*.

eFigure 2. Adjusted Language and Adaptive Behavioral Outcomes at Age 6 Years in Children of Women With Epilepsy Stratified by Breastfeeding Duration

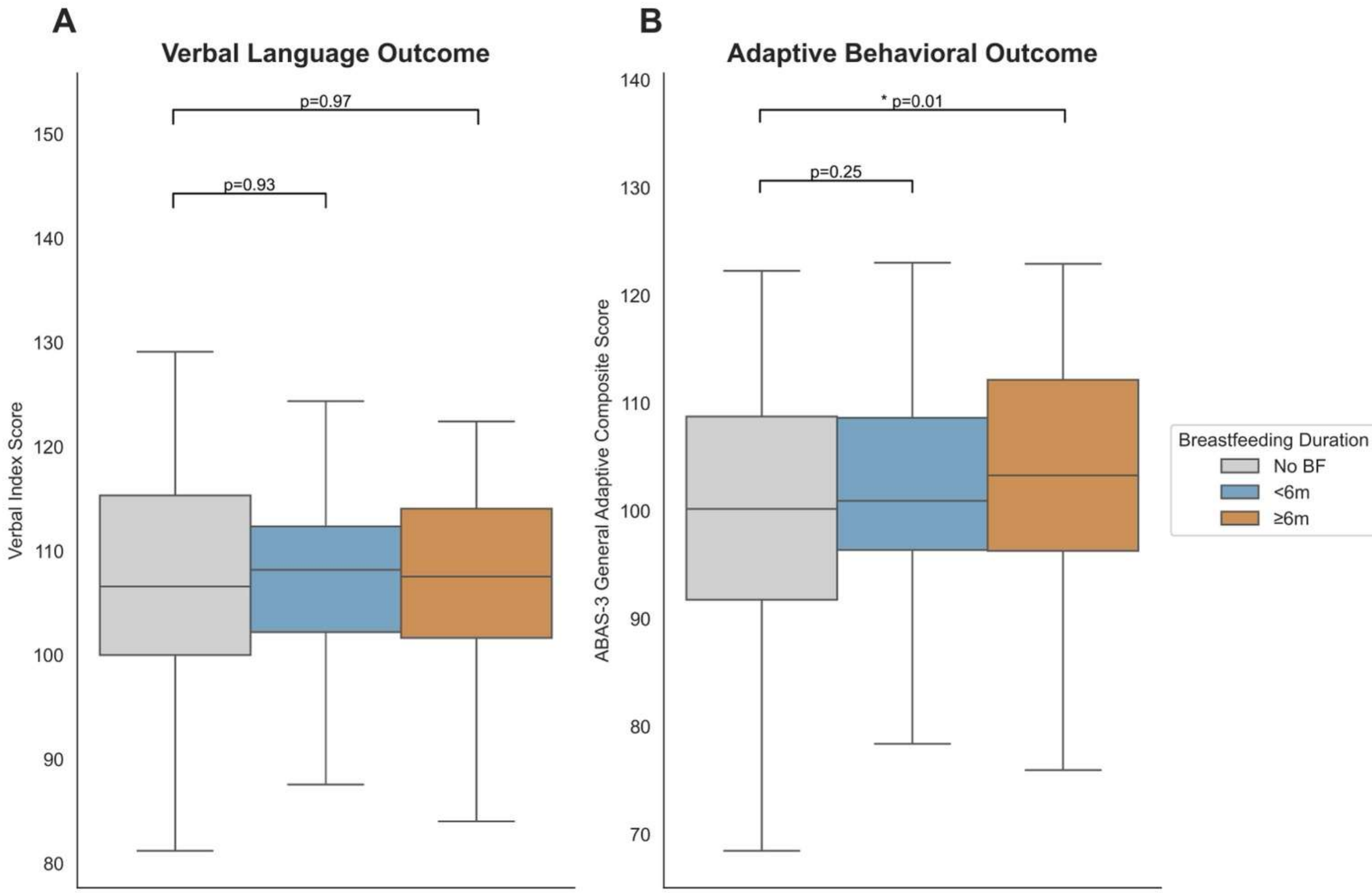

Outcomes are stratified by breastfeeding duration: no breastfeeding (reference group), 0 to 6 months, and ≥6 months. The analysis includes children of WWE taking anti-seizure medications. Data are presented as box-and-whisker plots; the box represents the interquartile range (IQR), the horizontal line inside the box represents the median, and the whiskers represent the range. (A) Language Outcomes measured by Verbal Index Score: scores were adjusted for maternal IQ, prenatal anxiety, maternal education, child sex, ethnicity, and folate supplementation. Adjusted mean scores ± SD were: No breastfeeding (n=67) 106.87 ± 11.59; Breastfeeding 0–6 months (n=75) 107.06 ± 9.71 (P = .93 vs reference); and Breastfeeding ≥6 months (n=120) 106.98 ± 9.24 (P = .97 vs reference). There were no statistically significant differences in language outcomes between groups. (B) Adaptive Behavioral Outcomes measured by ABAS-3 General Adaptive Composite Score: scores were adjusted for maternal age, history of depression, postpartum stress, and child race. Adjusted mean scores ± SD were: No breastfeeding (n=66) 99.84 ± 11.61; Breastfeeding 0–6 months (n=75) 101.32 ± 10.74 (P = .25 vs reference); and Breastfeeding ≥6 months (n=120) 103.58 ± 10.28 (P = .01 vs reference). Breastfeeding for ≥6 months was associated with significantly higher adaptive behavioral scores compared with the no breastfeeding group.

© 2026 Li Y et al. *JAMA Network Open*.

eFigure 3. Unadjusted Distributions of Adaptive Behavioral Subdomain Outcomes at Age 6 Years in Children of Women With Epilepsy Stratified by Breastfeeding Duration

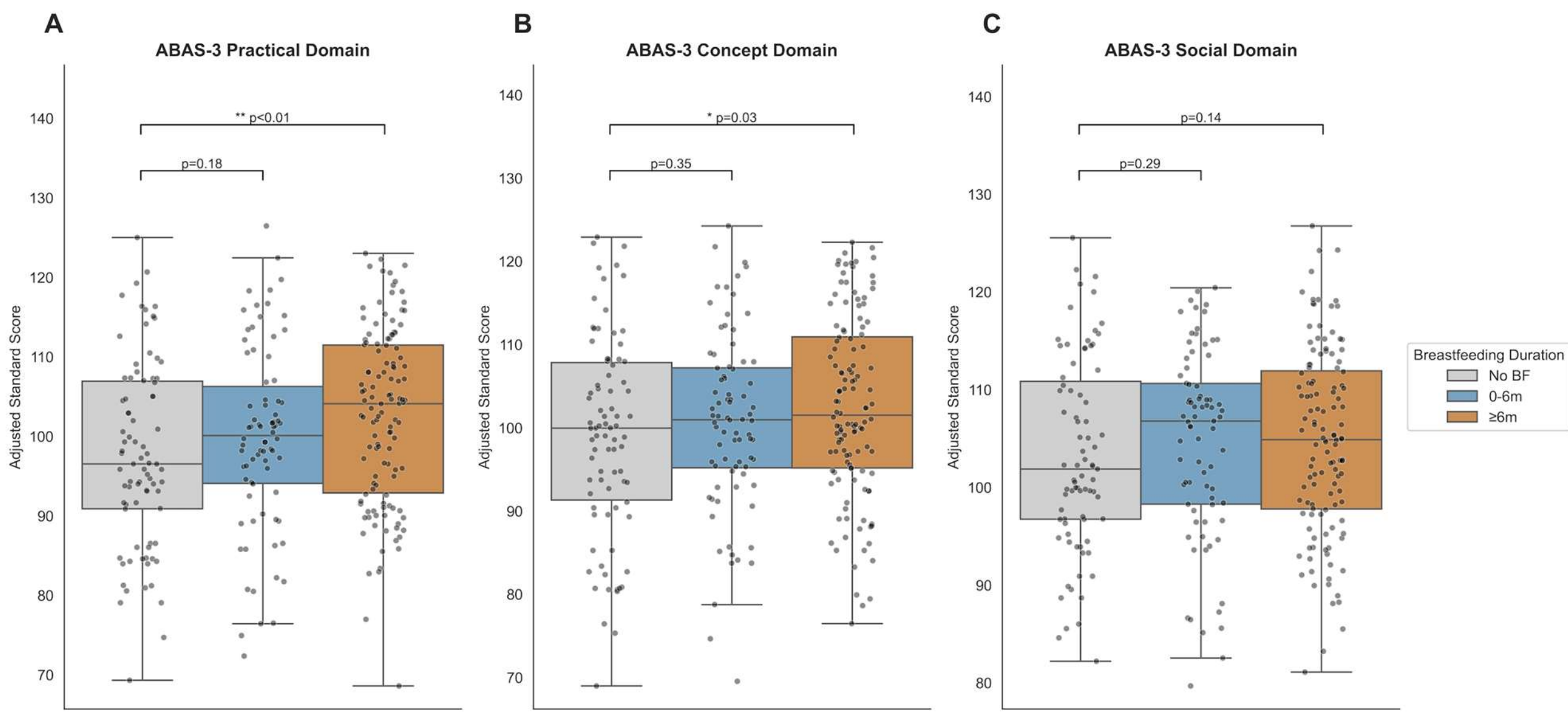

Figure Legend: Boxplots display the median (center line) and interquartile range (IQR; box limits). Superimposed jittered dots represent individual participant scores to demonstrate the raw data distribution. Data are presented for children of women with epilepsy (WWE) stratified by categorical breastfeeding (BF) duration (No BF, >0 to <6 months, and ≥6 months).

© 2026 Li Y et al. *JAMA Network Open*.

eFigure 4. Adaptive Behavioral Subdomain Outcomes at Age 6 Years by Breastfeeding Duration and Antiseizure Medication Regimens

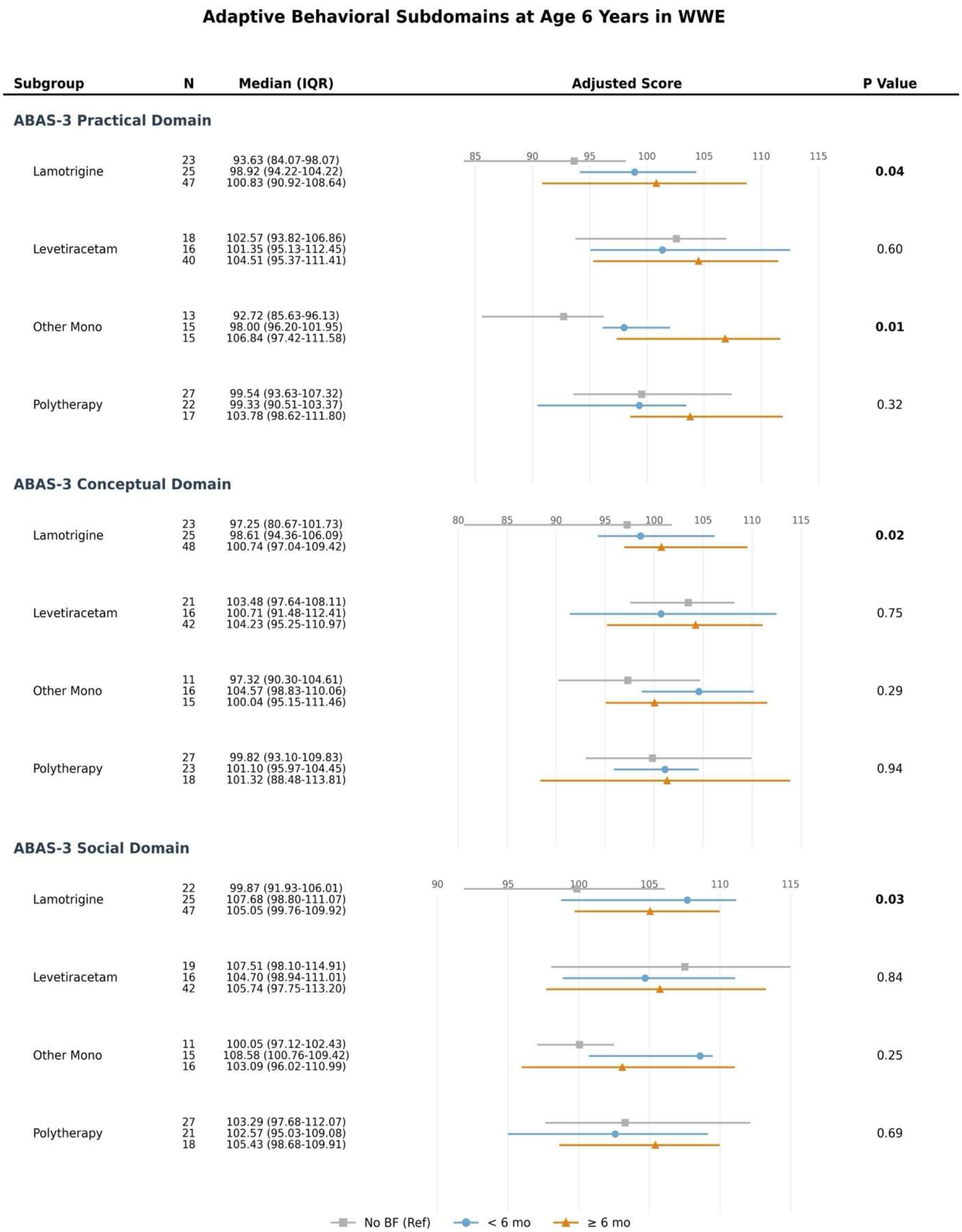

eFigure 5. Nonverbal Cognitive Outcomes at Age 6 Years by Breastfeeding Duration and Antiseizure Medication Regimens

© 2026 Li Y et al. *JAMA Network Open*.

Nonverbal Cognitive Outcomes at Age 6 Years in WWE

Subgroup	N	Median (IQR)	P Value
**DAS-II Nonverbal Cognitive Outcome**			
Lamotrigine	23	100.22 (94.56-111.75)	0.46
	25	105.83 (101.35-113.64)	
	46	106.11 (101.60-109.86)	
Levetiracetam	21	102.64 (99.22-112.07)	0.90
	15	103.71 (95.30-107.73)	
	40	102.99 (96.22-110.72)	
Other Mono	8	105.98 (103.84-110.65)	0.64
	14	103.04 (97.70-106.23)	
	17	105.48 (98.51-109.89)	
Polytherapy	25	109.10 (99.52-112.00)	0.54
	24	107.55 (99.73-112.06)	
	19	103.06 (97.81-109.12)	

Adjusted Score

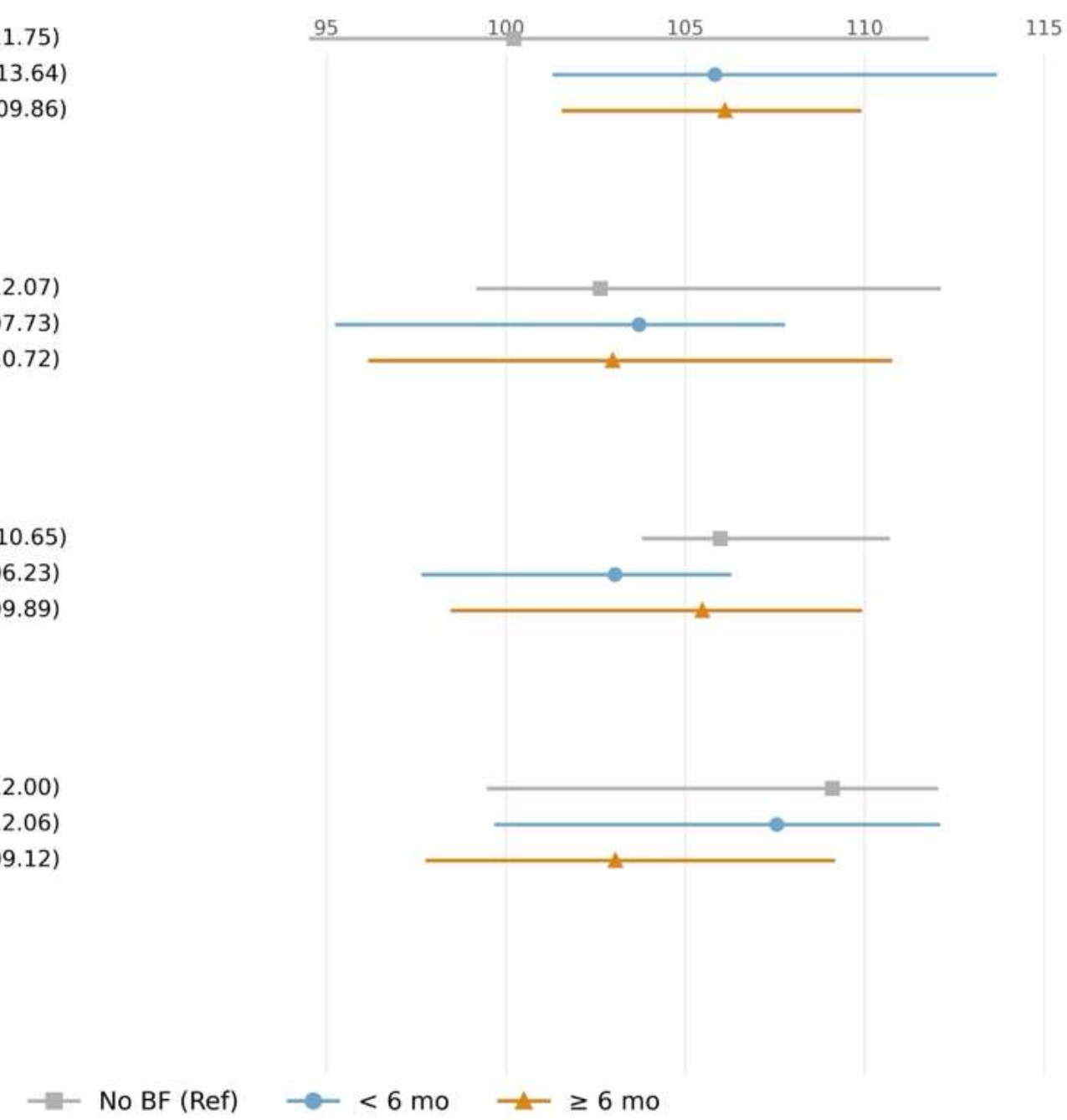

© 2026 Li Y et al. *JAMA Network Open*.

eFigure 6. General Conceptual Ability Outcomes at Age 6 Years by Breastfeeding Duration and Antiseizure Medication Regimens

General Conceptual Ability at Age 6 Years in WWE

Subgroup	N	Median (IQR)	P Value
**DAS-II General Conceptual Ability**			
Lamotrigine	23	105.12 (95.62-109.64)	0.29
	25	108.70 (102.13-113.13)	
	46	105.75 (103.17-113.73)	
Levetiracetam	21	109.83 (98.22-117.14)	0.42
	15	102.50 (97.67-111.17)	
	40	103.35 (95.39-111.40)	
Other Mono	9	107.02 (106.54-113.40)	0.70
	14	107.10 (100.96-109.52)	
	16	108.51 (102.71-113.56)	
Polytherapy	25	106.69 (98.42-114.42)	0.58
	24	108.02 (100.38-112.07)	
	18	104.12 (98.91-107.89)	

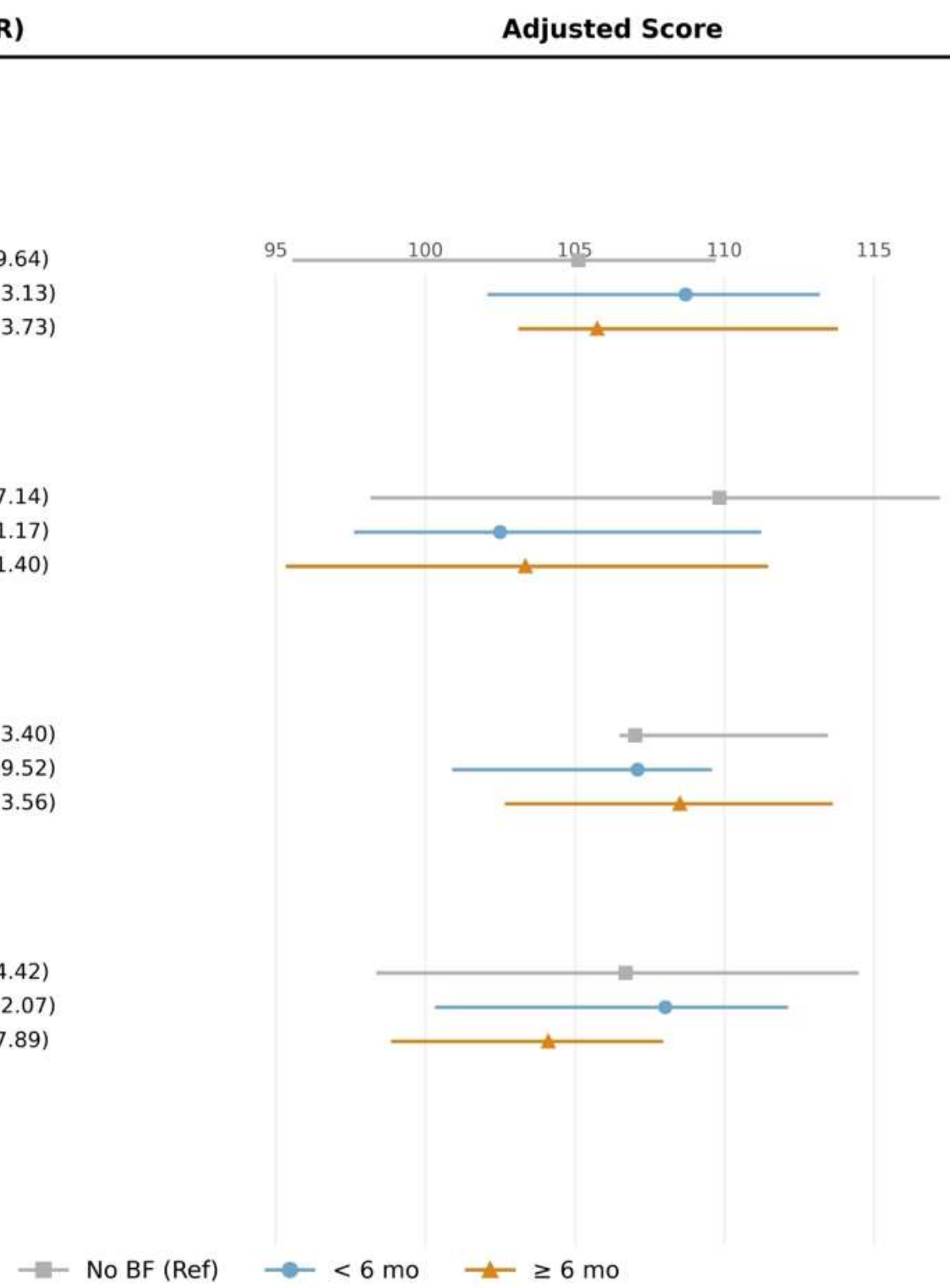

© 2026 Li Y et al. *JAMA Network Open*.

eFigure 7. Neurodevelopmental Outcomes at Age 6 Years by Breastfeeding Duration and Antiseizure Medication Regimen: Monotherapy vs Polytherapy

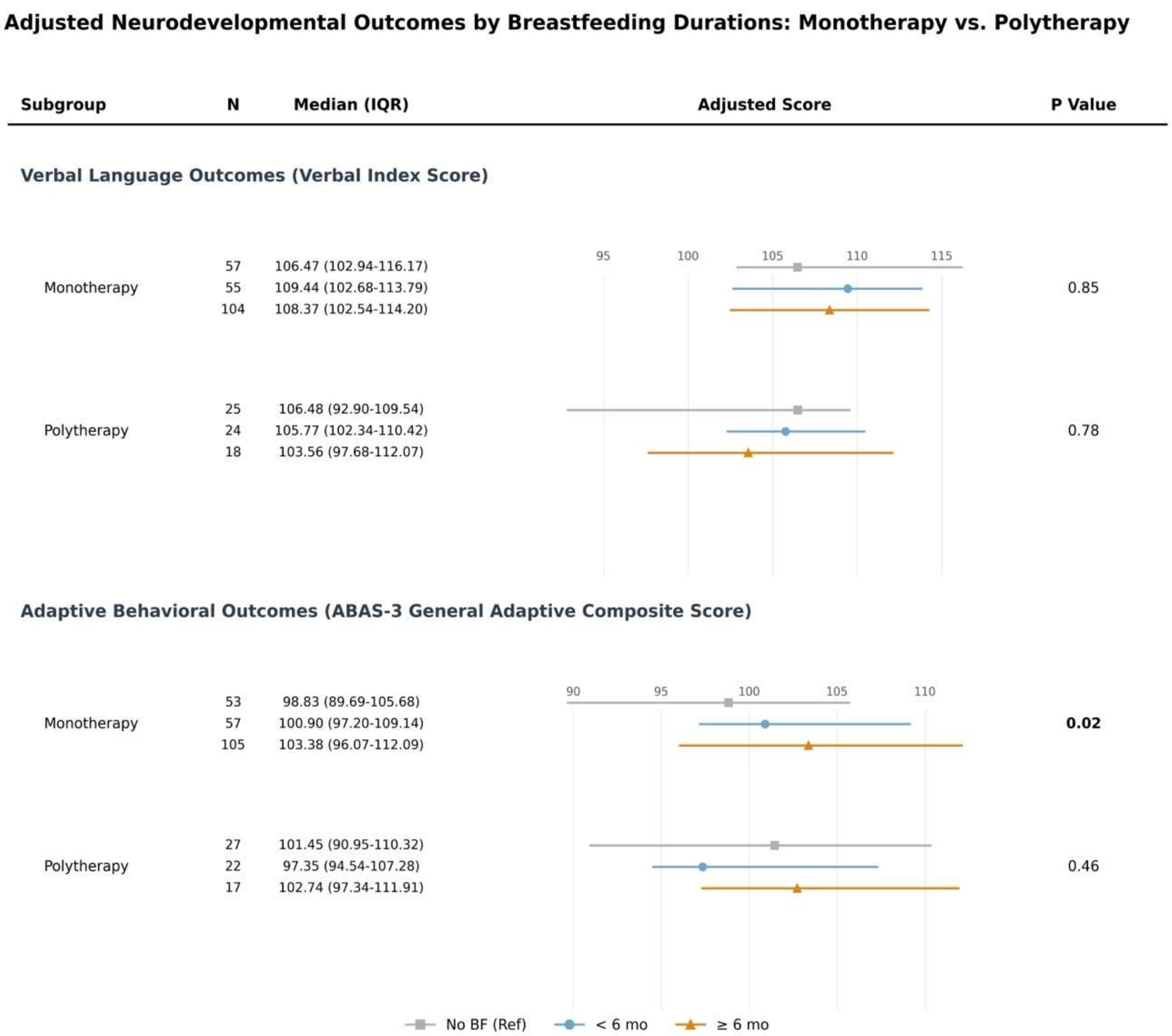

Figure Legend: Data are presented as medians (markers) and interquartile ranges (horizontal lines). (A) Verbal language Outcomes: adjusted Verbal Index Scores (VIS) are shown for each medication subgroup. Scores were adjusted for maternal IQ, prenatal anxiety, maternal education, child ethnicity, and folate supplementation. (B) Adaptive Behavioral Outcomes: adjusted ABAS-3 General Adaptive Composite (GAC) scores are shown for each medication subgroup. Scores were adjusted for maternal age, history of depression, postpartum stress, and birth weight. *P* values represent significance across the 3 breastfeeding groups, calculated using the Kruskal-Wallis test.

Abbreviations: ASM, antiseizure medication; IQ, intelligence quotient; ABAS-3, Adaptive Behavior Assessment System, Third Edition.

eFigure 8. Adaptive Behavioral Subdomain Outcomes at Age 6 Years by Breastfeeding Duration and Antiseizure Medication Type: Monotherapy vs Polytherapy

© 2026 Li Y et al. *JAMA Network Open*.

Adaptive Behavioral Subdomains at Age 6 Years in WWE: Monotherapy vs. Polytherapy

Subgroup	N	Median (IQR)	P Value
**ABAS-3 Practical Domain**			
Monotherapy	54	94.70 (84.58-104.14)	**<.01**
	56	100.77 (95.27-106.49)	
	102	103.55 (91.65-111.10)	
Polytherapy	27	99.54 (93.63-107.32)	0.32
	22	99.33 (90.51-103.37)	
	17	103.78 (98.62-111.80)	
**ABAS-3 Conceptual Domain**			
Monotherapy	55	99.90 (90.30-106.80)	0.14
	57	100.74 (94.36-108.70)	
	105	101.63 (95.80-110.59)	
Polytherapy	27	99.82 (93.10-109.83)	0.94
	23	101.10 (95.97-104.45)	
	18	101.32 (88.48-113.81)	
**ABAS-3 Social Domain**			
Monotherapy	52	100.29 (95.14-109.29)	0.12
	56	107.38 (100.03-110.81)	
	105	105.05 (98.26-112.10)	
Polytherapy	27	103.29 (97.68-112.07)	0.69
	21	102.57 (95.03-109.08)	
	18	105.43 (98.68-109.91)	

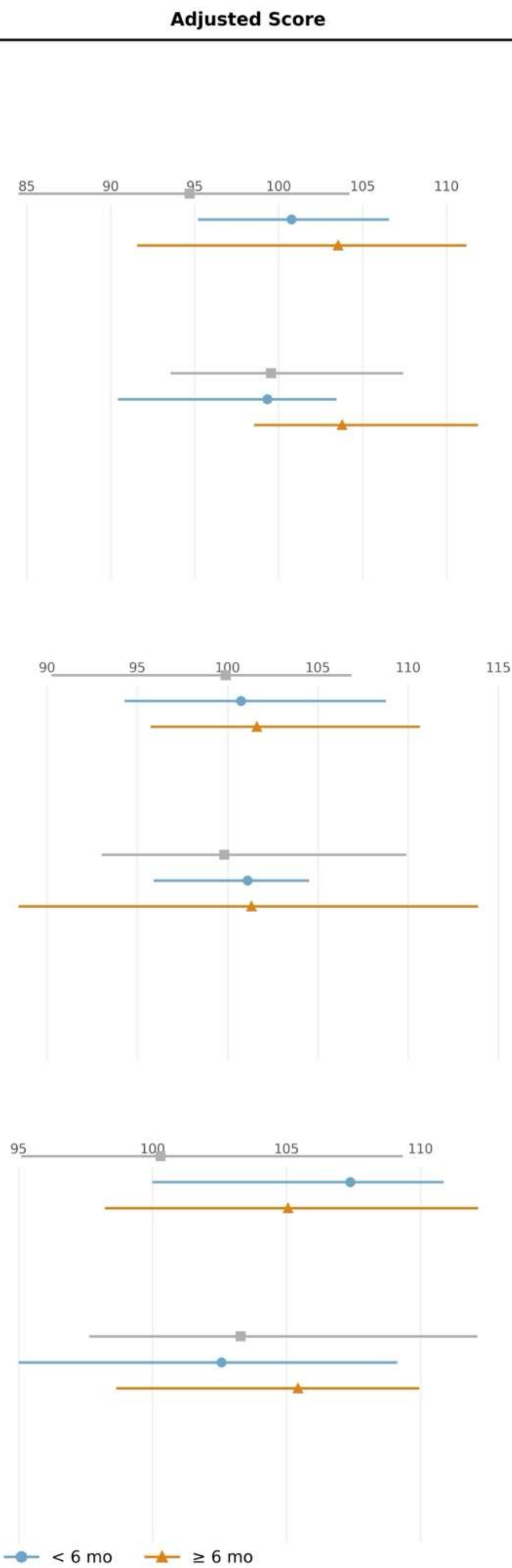

© 2026 Li Y et al. *JAMA Network Open*.

eFigure 9. Nonverbal Cognitive Outcomes at Age 6 Years by Breastfeeding Duration and Antiseizure and Antiseizure Medication Type: Monotherapy vs Polytherapy

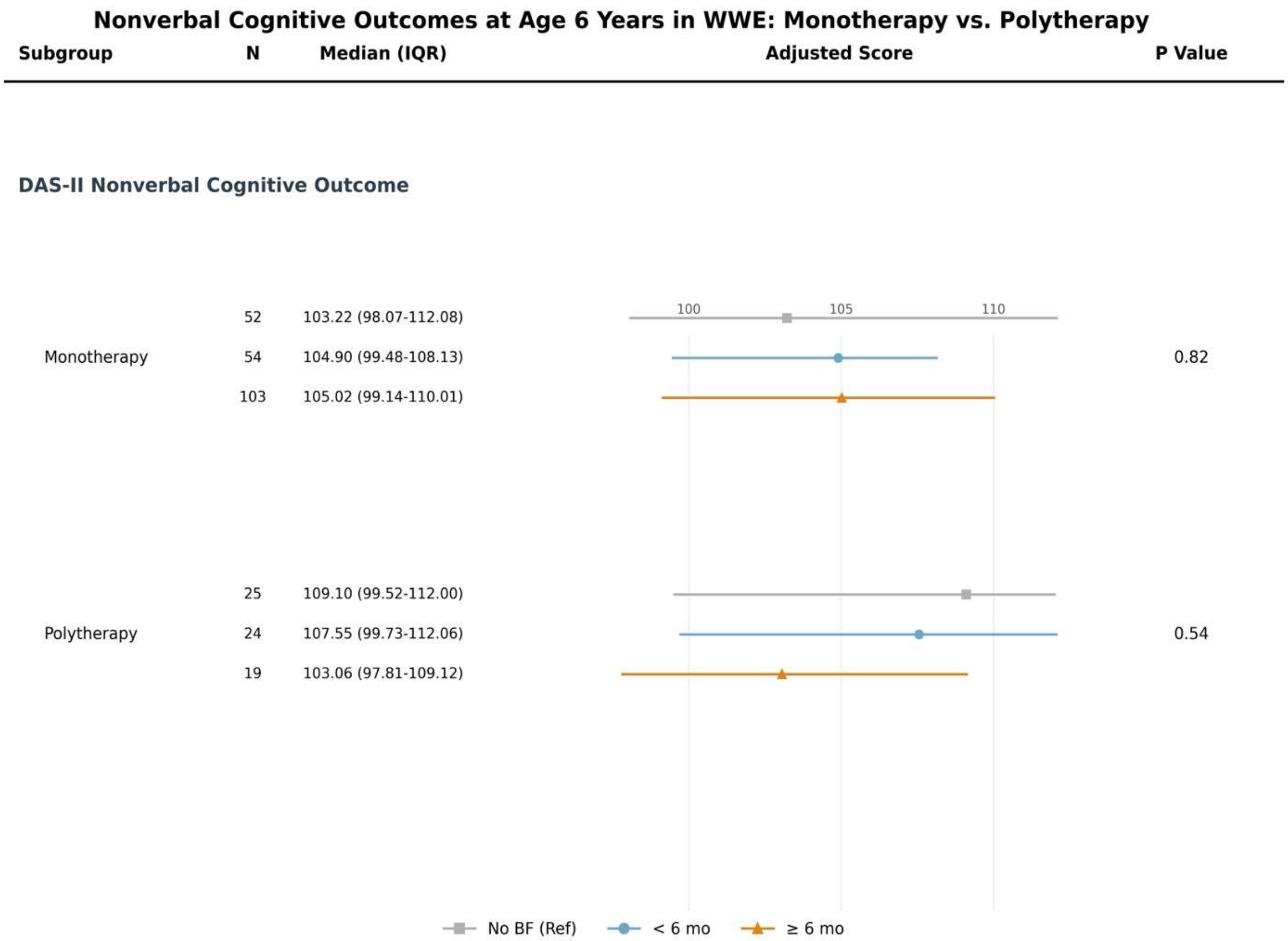

© 2026 Li Y et al. *JAMA Network Open*.

eFigure 10. General Conceptual Ability Outcomes at Age 6 Years by Breastfeeding Duration and Antiseizure and Antiseizure Medication Type: Monotherapy vs Polytherapy

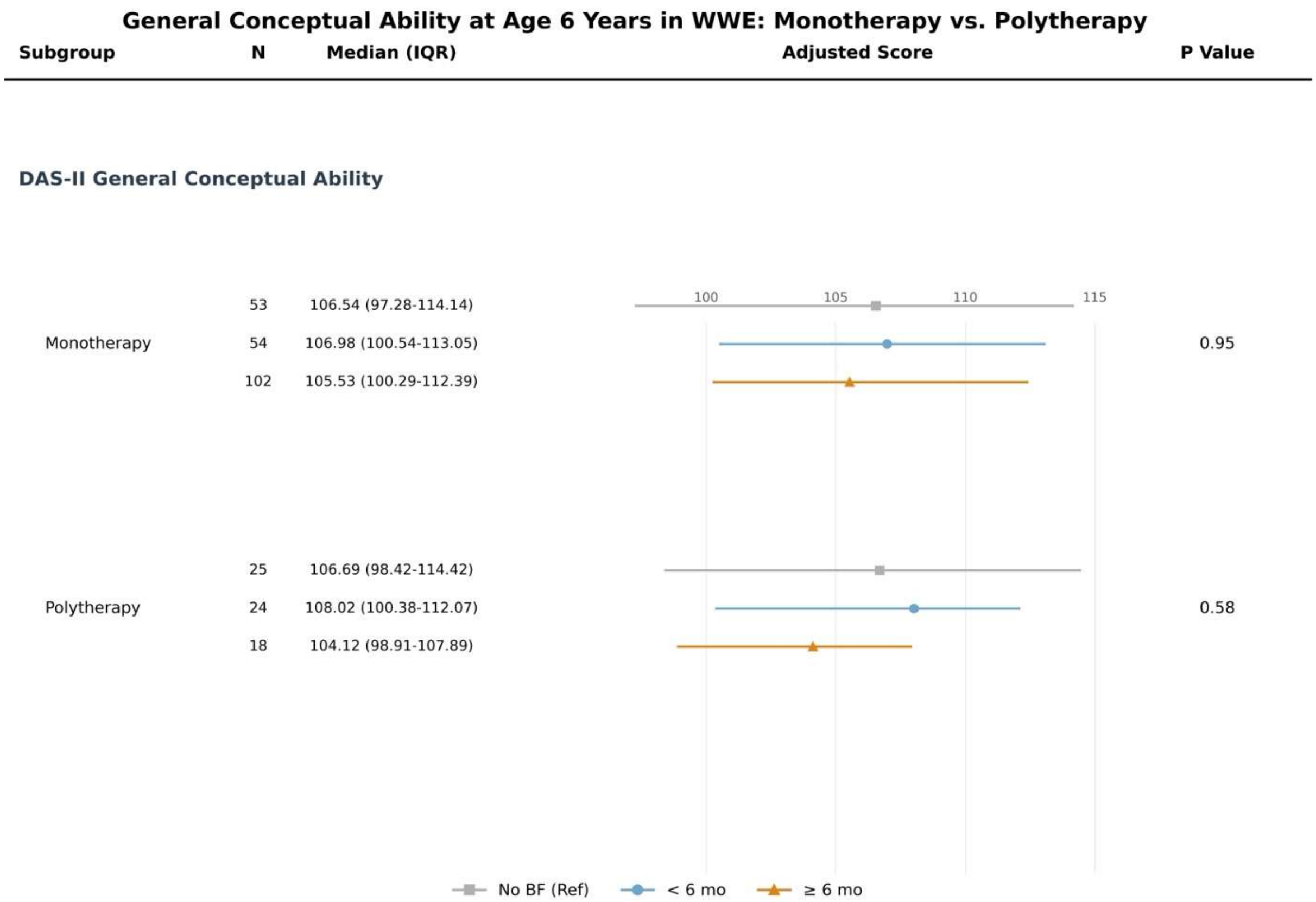

© 2026 Li Y et al. *JAMA Network Open*.

eTable 1. Baseline Demographic and Clinical Characteristics of Language Outcome Analysis: Women With Epilepsy vs Healthy Women

Variable	WWE (N=262)	HW (N=80)	P-Value
Categorical variables			
Child's Sex – Male	120 (45.8%)	43 (53.8%)	0.25
Child's Race			0.01
Black or African American	11 (4.2%)	8 (10.0%)	
Other/Unknown	36 (13.7%)	19 (23.8%)	
White	215 (82.1%)	53 (66.2%)	
Child's Ethnicity – Hispanic or Latino	51 (19.5%)	20 (25.0%)	0.34
Twins	22 (8.4%)	6 (7.5%)	1
Small for Gestational Age (SGA)	15 (5.7%)	5 (6.2%)	0.79
Breastfeeding Status			<0.001
None	67/262 (25.6%)	9/80 (11.2%)	
0-6 months	75/262 (28.6%)	14/80 (17.5%)	
>=6 months	120/262 (45.8%)	57/80 (71.2%)	
Periconceptional Folate Use (Yes/No)	231 (88.2%)	55 (68.8%)	<0.01
Periconceptional Folate Dose			<0.01
0 mg	31 (11.8%)	25 (31.2%)	
>=0 - 0.4 mg	21 (8.0%)	10 (12.5%)	
>=0.4 - 1 mg	44 (16.8%)	31 (38.8%)	
>=1 - 4 mg	143 (54.6%)	12 (15.0%)	
>=4 mg	22 (8.4%)	0 (0.0%)	
Avg Folate Dose in First 12 Weeks			<0.01
0 mg	5 (1.9%)	2 (2.5%)	
>=0 - 0.4 mg	3 (1.1%)	11 (13.8%)	
>=0.4 - 1 mg	21 (8.0%)	52 (65.0%)	
>=1 - 4 mg	132 (50.4%)	12 (15.0%)	
>=4 mg	93 (35.5%)	0 (0.0%)	
Pregnancy Planned/Welcomed			0.85
Planned	192 (73.3%)	56 (70.0%)	
Unplanned/Welcome	64 (24.4%)	22 (27.5%)	
Unplanned/Unwelcome	6 (2.3%)	2 (2.5%)	
Major Depressive Episode During Pregnancy	11 (4.2%)	2 (2.5%)	0.74
Mother's Education			0.34
No College Degree	69 (26.3%)	20 (25.0%)	
College Degree (Not Advanced)	123 (46.9%)	32 (40.0%)	

© 2026 Li Y et al. *JAMA Network Open*.

College Degree (Advanced)	70 (26.7%)	28 (35.0%)	
Mother's Employment			0.45
Employed full-time	147 (56.1%)	47 (58.8%)	
Employed part-time	39 (14.9%)	15 (18.8%)	
Unemployed*	76 (29.0%)	18 (22.5%)	
Household Income			0.9
$24,999 or less	44 (16.8%)	12 (15.0%)	
$25,000-49,999	25 (9.5%)	8 (10.0%)	
$50,000-74,999	40 (15.3%)	9 (11.2%)	
$75,000-99,999	45 (17.2%)	16 (20.0%)	
$100,000 and up	97 (37.0%)	30 (37.5%)	
History of Smoking#	15 (5.7%)	3 (3.8%)	0.78
History of Alcohol Use	62 (23.7%)	29 (36.2%)	0.03
Lifetime History of Anxiety Disorder	38 (14.5%)	11 (13.8%)	1
Lifetime History of Depression Disorder	43 (16.4%)	9 (11.2%)	0.37

	**Children of WWE (N = 262)**					**Children of HW (n=80)**					
	**N**	**Mean**	**Min**	**Max**	**SE**	**N**	**Mean**	**Min**	**Max**	**SE**	**P value**
Continuous Variables											
WGA at Birth	262	38.42	24	42	0.13	80	38.49	26	43	0.23	1
WGA at Enrollment	262	13.37	4	29	0.29	80	15.44	5	23	0.44	<.001
Birthweight (kg)	257	3.22	0.62	4.9	0.04	80	3.26	1.08	5.03	0.07	0.85
Mother's Age at Enrollment	262	30.89	19	46	0.31	80	29.83	15	38	0.55	0.12
Mother's IQ	262	98.76	65	123	0.77	80	104.8	72	131	1.44	<.001
BDI during pregnancy	262	7.02	0	38.7	0.36	80	5.75	0	15.67	0.39	0.21
BDI post-birth	262	5.77	0	33.4	0.34	80	4.7	0	29.88	0.49	0.18
BDI overall	262	6.12	0.2	32.5	0.32	80	5.03	0	26	0.43	0.17
BAI during pregnancy	262	6.16	0	38.3	0.35	80	4.9	0	15.67	0.39	0.24
BAI post-birth	262	4.34	0	31.5	0.28	80	2.87	0	25.5	0.38	0
BAI overall	262	4.86	0	33.4	0.28	80	3.44	0	20.82	0.33	0.02
PSS during pregnancy	262	17.87	2.67	40	0.45	80	16.46	3.33	28.67	0.63	0.2
PSS post-birth	262	18.47	2.43	37.8	0.4	80	18.28	8.5	34.13	0.57	0.79
PSS overall	262	18.3	3.3	38.4	0.38	80	17.76	9.45	29.91	0.52	0.4
PSQI during[&] pregnancy	242	5.85	0.42	15	0.19	72	4.75	1	11.8	0.29	0.01
PSQI postpartum[&]	214	5.67	1	13.6	0.19	62	5.77	0.83	11.2	0.34	0.51
PSQI overall[&]	212	5.76	1.35	13.7	0.18	62	5.31	1.17	11	0.28	0.26

Data are presented as n (%) for categorical variables and mean ± standard error (SE) for continuous variables. The cohort includes all mother-child pairs from the MONEAD study with available language outcome data at age 6 (VIS

score). P-values were derived from Chi-squared or Fisher's exact tests for categorical data. For continuous data, group comparisons were made using Student's t-test or the non-parametric Mann-Whitney U test, depends on the normality assessments with the Shapiro-Wilk test. A p-value < 0.05 was considered statistically significant.
Abbreviations: WWE, women with epilepsy; HW, healthy women; WGA, weeks gestational age; BDI, Beck Depression Inventory; BAI, Beck Anxiety Inventory; PSS, Perceived Stress Scale; PSQI, Pittsburgh Sleep Quality Index.
Notes: Missing not used in calculation of percentages.
WGA = weeks gestational age.
* Unemployed includes stay at home parent without outside job, unemployed due to disability, full-time student and looking for work.
#Self-reported smoking, alcohol use, or illicit substance use (including marijuana) at any time during pregnancy.
[&]Weighted monthly average of PSQI assessments completed during time period of enrollment through the day before delivery. At most one PSQI score per day was used in the calculation.

© 2026 Li Y et al. *JAMA Network Open*.

eTable 2. Baseline Demographic and Clinical Characteristics of Adaptive Behavior Analysis: Women With Epilepsy vs Healthy Women

Variable	WWE (N=261)	HW (N=83)	P-Value
Categorical Variables			
Child's Sex – Male	116 (44.4%)	46 (55.4%)	0.10
Child's Race			0.02
Black or African American	11 (4.2%)	8 (9.6%)	
Other/Unknown	37 (14.2%)	19 (22.9%)	
White	213 (81.6%)	56 (67.5%)	
Child's Ethnicity – Hispanic or Latino	54 (20.7%)	21 (25.3%)	0.37
Twins	21 (8.0%)	6 (7.2%)	1.00
Small for Gestational Age (SGA)	15 (5.7%)	5 (6.0%)	1.00
Major Congenital Malformations	13 (5.0%)	1 (1.2%)	0.20
Breastfeeding Status			0.001
None	66/261 (25.3%)	11/83 (13.3%)	
0-6 months	75/261 (28.7%)	15/83 (18.1%)	
>=6 months	120/261 (46.0%)	57/83 (68.7%)	<.001
Periconceptional Folate Use (Yes/No)	229 (87.7%)	56 (67.5%)	<.001
Periconceptional Folate Dose			<.001
0 mg	32 (12.3%)	27 (32.5%)	
>=0 - 0.4 mg	20 (7.7%)	11 (13.3%)	
>=0.4 - 1 mg	46 (17.6%)	31 (37.3%)	
>=1 - 4 mg	140 (53.6%)	12 (14.5%)	
>=4 mg	22 (8.4%)	0 (0.0%)	
Avg Folate Dose in First 12 Weeks			<.001
0 mg	4 (1.5%)	3 (3.6%)	
>=0 - 0.4 mg	4 (1.5%)	11 (13.3%)	
>=0.4 - 1 mg	21 (8.0%)	54 (65.1%)	
>=1 - 4 mg	133 (51.0%)	12 (14.5%)	
>=4 mg	91 (34.9%)	0 (0.0%)	
Pregnancy Planned/Welcomed			0.73
Planned	188 (72.0%)	56 (67.5%)	
Unplanned/Welcome	65 (24.9%)	24 (28.9%)	
Unplanned/Unwelcome	8 (3.1%)	3 (3.6%)	
Major Depressive Episode During Pregnancy	12 (4.6%)	2 (2.4%)	0.53
Mother's Education			0.50
No College Degree	72 (27.6%)	23 (27.7%)	
College Degree (Not Advanced)	120 (46.0%)	33 (39.8%)	

© 2026 Li Y et al. *JAMA Network Open*.

College Degree (Advanced)	69 (26.4%)	27 (32.5%)	
Mother's Employment			0.60
Employed full-time	147 (56.3%)	46 (55.4%)	
Employed part-time	39 (14.9%)	16 (19.3%)	
Unemployed	75 (28.7%)	21 (25.3%)	
Household Income			0.82
$24,999 or less	46 (17.6%)	14 (16.9%)	
$25,000-49,999	23 (8.8%)	9 (10.8%)	
$50,000-74,999	40 (15.3%)	9 (10.8%)	
$75,000-99,999	48 (18.4%)	16 (19.3%)	
$100,000 and up	95 (36.4%)	30 (36.1%)	
History of smoking	16 (6.1%)	4 (4.8%)	0.79
History of Alcohol Use	65 (24.9%)	30 (36.1%)	0.05
Lifetime History of Anxiety Disorder	40 (15.3%)	12 (14.5%)	1.00
Lifetime History of Depression Disorder	44 (16.9%)	9 (10.8%)	0.29

	Children of WWE (N = 261)					Children of HW (n=83)					
	N	Mean	Min	Max	SE	N	Mean	Min	Max	SE	p value
Continuous Variables											
WGA at Birth	261	38.49	24	42	0.12	83	38.51	26	43	0.22	0.75
WGA at Enrollment	261	13.42	4.00	29.00	0.29	83	15.28	5.00	23.00	0.44	0.00
Birthweight (kg)	256	3.24	0.62	4.90	0.04	83	3.27	1.08	5.03	0.07	0.89
Mother's Age at Enrollment	261	30.85	19.00	45.00	0.31	83	29.70	15.00	38.00	0.53	0.07
Mother's IQ	261	98.68	65.00	123.00	0.76	83	104.31	72.00	131.00	1.36	<.001
BDI during pregnancy	261	7.16	0.00	38.67	0.36	83	5.91	0.00	17.67	0.42	0.18
BDI post-birth	261	5.77	0.00	33.38	0.34	83	4.87	0.00	29.88	0.50	0.24
BDI overall	261	6.15	0.20	32.45	0.32	83	5.19	0.00	26.00	0.44	0.19
BAI during pregnancy	261	6.33	0.00	38.33	0.36	83	5.29	0.00	29.33	0.52	0.18
BAI post-birth	261	4.42	0.00	31.50	0.30	83	3.30	0.00	25.50	0.51	0.01
BAI overall	261	4.97	0.00	33.36	0.29	83	3.87	0.00	26.60	0.48	0.03
PSS during pregnancy	261	18.01	2.67	40.00	0.46	83	16.58	3.33	28.67	0.63	0.21
PSS post-birth	261	18.55	2.43	37.75	0.40	83	18.30	8.50	34.13	0.57	0.69
PSS overall	261	18.39	3.30	38.36	0.39	83	17.80	9.45	29.91	0.52	0.42

© 2026 Li Y et al. *JAMA Network Open*.

PSQI during pregnancy	242	5.80	0.14	14.00	0.19	74	4.77	1.00	11.80	0.30	0.01
PSQI postpartum	211	5.66	0.00	13.58	0.19	63	5.81	0.83	12.50	0.35	0.55
PSQI overall	209	5.74	0.08	13.70	0.18	63	5.37	1.17	12.25	0.30	0.26

Data are presented as n (%) for categorical variables and mean ± standard error (SE) for continuous variables. The cohort includes all mother-child pairs from the MONEAD study with available adaptive behavior outcome data at age 6 (ABAS-3 General Adaptive Composite Score).
Abbreviations: WWE, women with epilepsy; HW, healthy women; WGA, weeks gestational age; BDI, Beck Depression Inventory; BAI, Beck Anxiety Inventory; PSS, Perceived Stress Scale; PSQI, Pittsburgh Sleep Quality Index.

© 2026 Li Y et al. *JAMA Network Open*.

eTable 3. Multivariable Logistic Regression Model of Factors Associated With Breastfeeding (≥6 Months) in the Whole Cohort (Women With Epilepsy and Healthy Women)

Variables	Odds Ratio (95% CI)	P-Value
WWE vs. HW[#]	0.63 (0.34, 1.16)	0.14
History of smoking	0.16 (0.04, 0.75)	0.02
Mean Beck Anxiety Inventory during pregnancy	0.87 (0.82, 0.94)	<.001
Mother's IQ	1.04 (1.01, 1.06)	0.002
Mean Perceived Stress Scale-14 post-birth (delivery through 6yo visit)	1.07 (1.02, 1.12)	0.01
Birthweight (kg)	1.68 (1.09, 2.59)	0.02

WWE vs. HW was a prior.
Abbreviations: WWE, women with epilepsy; HW, healthy women.

© 2026 Li Y et al. *JAMA Network Open*.

eTable 4. Multivariable Logistic Regression Model of Factors Associated With Breastfeeding (≥6 Months) Among Women With Epilepsy

Variables	Odds Ratio (95% CI)	P-Value
Mother's IQ	1.04 (1.02 - 1.07)	<0.001
BAI score during pregnancy	0.94 (0.90 - 0.99)	0.02
History of smoking (yes/no)	0.21 (0.05 - 0.95)	0.04
Seizure Types		
-Focal epilepsy	reference	-
-Generalized epilepsy	0.53 (0.31 - 0.90)	0.02
-Mixed/Unclassified Epilepsy	0.29 (0.10 - 0.85)	0.02

The model was derived from a stepwise logistic regression algorithm to identify significant predictors of breastfeeding for six months or longer. Results are presented as odds ratios with 95% confidence intervals (CI).
Abbreviations: BAI, Beck Anxiety Inventory.

© 2026 Li Y et al. *JAMA Network Open*.

eTable 5. Language and Behavior Outcome in Children of Women With Epilepsy vs Healthy Controls Following Breastfeeding Exposure

Group	Unadjusted Analysis	No	P value	Difference (95% CI)	Adjusted Analysis	No	P value	Difference (95% CI)
Language Outcome (Verbal Index Score) [#]								
HW	108.39 (11.31)	77	-	-	107.18 (1.40)	53	-	-
WWE	107.01 (12.01)	251	0.37	-1.38 (-4.41, 1.66)	107.93 (0.71)	192	0.64	0.75 (-2.40, 3.89)
Adaptive Behavior Outcome (ABAS-3 GAC) [†]								
HW	102.59 (11.36)	80	-	-	102.14 (1.46)	53	-	-
WWE	101.98 (11.55)	250	0.68	-0.61 (-3.52, 2.30)	102.91 (0.75)	189	0.64	0.78 (-2.52, 4.07)

Data are presented as mean (standard deviation) for unadjusted descriptive statistics and adjusted least-squares (LS) mean (standard error) for adjusted analyses.
#Adjusted LS means for the Verbal Index Score were controlled for breastfeeding duration, mother's age at enrollment, maternal IQ, mean Beck Anxiety Inventory score during pregnancy, child's sex, and child ethnicity.
†Adjusted LS means for the ABAS-3 General Adaptive Composite Score were controlled for breastfeeding duration, mother's age at enrollment, birthweight, mean Perceived Stress Scale score post-birth, child's sex, and children's race.
Abbreviations: ABAS-3 GAC, Adaptive Behavior Assessment System–Third Edition General Adaptive Composite; CI, confidence interval; HW, healthy women; WWE, women with epilepsy.

© 2026 Li Y et al. *JAMA Network Open*.

eTable 6. Multivariable Regression Models Comparing Verbal Index Scores and Adaptive Behavioral Outcomes at Age 6 Years Between Children of Women With Epilepsy and Healthy Women

Variable	Coefficient (95% CI)	p-value
**Language Outcome (Verbal Index Score)**		
**Cohort Group**		
- Healthy Women	Reference	—
**- Women with Epilepsy**	**0.75 (-2.40, 3.89)**	**0.64**
**Breastfeeding Duration**		
- No Breastfeeding	Reference	—
- 0 < Breastfeeding < 6m	-0.71 (-4.51, 3.09)	0.71
- Breastfeeding >= 6m	-0.26 (-3.88, 3.36)	0.89
Mother's Age at Enrollment	0.30 (0.04, 0.56)	0.03
Maternal IQ	0.33 (0.22, 0.43)	< .001
Mean Beck Anxiety Inventory during pregnancy	-0.46 (-0.71, -0.21)	< .001
Child's sex (Male vs. Female)	-3.55 (-6.06, -1.03)	0.01
Child Ethnicity		
- Hispanic or Latino	Reference	—
- Non-Hispanic or Non-Latino	5.00 (1.38, 8.62)	0.01
**Adaptive Behavioral Outcome (ABAS-3 General Adaptive Composite Score)**		
**Cohort Group**		
- Healthy Women	Reference	—
**- Women with Epilepsy**	**0.78 (-2.52, 4.07)**	**0.64**
**Breastfeeding Duration**		
- No Breastfeeding	Reference	—
- 0 < Breastfeeding < 6m	0.76 (-3.25, 4.77)	0.71
- Breastfeeding >= 6m	2.99 (-0.62, 6.60)	0.11
Mother's Age at Enrollment	-0.36 (-0.65, -0.08)	0.01
Birthweight (kg)	4.12 (1.76, 6.49)	< .001
Mean Perceived Stress Scale post-birth	-0.46 (-0.68, -0.25)	< .001
Child's sex (Male vs. Female)	-2.78 (-5.39, -0.17)	0.04
Children's race		
- White	Reference	—
- Black	13.97 (8.30, 19.65)	< .001
- Others/Unknown	1.72 (-1.89, 5.33)	0.35

Cohort group and breastfeeding duration were forced into all steps of the modeling process.
Abbreviations: ABAS-3 GAC, Adaptive Behavior Assessment System–Third Edition General Adaptive Composite; CI, confidence interval; IQ, intelligence quotient.

© 2026 Li Y et al. *JAMA Network Open*.

eTable 7. Multivariable Regression Models Comparing Adaptive Behavioral Subdomain Outcomes at Age 6 Years Between Children of Women With Epilepsy and Healthy Women

Variable	Coefficient (95% CI)	p-value
--- Behavioral Subdomain Outcomes ---		
**ABAS-3 Practical**		
**Cohort Group**		
- Healthy Women	Reference	—
**- Women with Epilepsy**	**1.48 (-2.15, 5.11)**	**0.42**
Breastfeeding Duration		
- No Breastfeeding	Reference	—
- 0 < Breastfeeding < 6m	2.86 (-1.50, 7.22)	0.20
- Breastfeeding >= 6m	4.34 (0.41, 8.28)	0.03
Mother's Age at Enrollment	-0.39 (-0.70, -0.09)	0.01
Birthweight (kg)	4.01 (1.47, 6.54)	0.002
Mean Perceived Stress Scale postpartum	-0.50 (-0.73, -0.27)	< .001
Children's race		
- White	Reference	—
- Black	14.13 (7.87, 20.38)	< .001
- Others/Unknown	0.56 (-3.43, 4.54)	0.78
**ABAS-3 Social**		
Cohort Group		
- Healthy Women	Reference	—
**- Women with Epilepsy**	**1.04 (-2.14, 4.21)**	**0.52**
Breastfeeding Duration		
- No Breastfeeding	Reference	—
- 0 < Breastfeeding < 6m	1.64 (-2.23, 5.51)	0.41
- Breastfeeding >= 6m	1.97 (-1.47, 5.42)	0.26
Mean Perceived Stress Scale postpartum	-0.41 (-0.61, -0.20)	< .001
Child's sex (Male vs. Female)	-3.27 (-5.79, -0.75)	0.01
Children's race		
- White	Reference	—
- Black	8.85 (3.41, 14.29)	0.002
- Others/Unknown	1.83 (-1.68, 5.33)	0.31
**ABAS-3 Conceptual**		
Cohort Group		
- Healthy Women	Reference	—
**- Women with Epilepsy**	**0.30 (-3.12, 3.72)**	**0.86**
Breastfeeding Duration		
- No Breastfeeding	Reference	—
- 0 < Breastfeeding < 6m	-0.19 (-4.31, 3.93)	0.93
- Breastfeeding >= 6m	2.55 (-1.22, 6.33)	0.18
Mother's Age at Enrollment	-0.35 (-0.66, -0.04)	0.03

© 2026 Li Y et al. *JAMA Network Open*.

Maternal Education		
- No College Degree	Reference	—
- College Degree (Not Advanced)	1.99 (-1.61, 5.59)	0.28
- College Degree (Advanced)	5.70 (1.57, 9.84)	0.01
Birthweight (kg)	3.66 (1.23, 6.09)	0.003
History of Depression (Yes vs. No)	-2.01 (-3.98, -0.04)	0.05
Mean Perceived Stress Scale post-birth	-0.24 (-0.47, -0.02)	0.03
Child's sex (Male vs. Female)	-3.00 (-5.70, -0.30)	0.03
Children's race		
- White	Reference	—
- Black	14.53 (8.61, 20.45)	< .001
- Others/Unknown	2.53 (-1.22, 6.27)	0.19

Cohort group and breastfeeding duration were forced into all steps of the modeling process. Abbreviations: ABAS-3, Adaptive Behavior Assessment System–Third Edition; CI, confidence interval.

© 2026 Li Y et al. *JAMA Network Open*.

eTable 8. Multivariable Regression Models Comparing Nonverbal Ability at Age 6 Years Between Children of Women With Epilepsy and Healthy Women

**Variable**	**Coefficient (95% CI)**	**p-value**
Cohort Group		
- Healthy Women	Reference	—
**- Women with Epilepsy**	**1.08 (-2.28, 4.44)**	**0.53**
Breastfeeding Duration		
- No Breastfeeding	Reference	—
- 0 < Breastfeeding < 6m	-2.49 (-6.69, 1.70)	0.24
- Breastfeeding >= 6m	-1.42 (-5.43, 2.58)	0.49
Maternal IQ	0.38 (0.26, 0.50)	< .001
Mean Beck Anxiety Inventory during pregnancy	-0.35 (-0.62, -0.08)	0.01

Cohort group and breastfeeding duration were forced into all steps of the modeling process.
Abbreviations: CI, confidence interval; IQ, intelligence quotient.

© 2026 Li Y et al. *JAMA Network Open*.

eTable 9. Multivariable Regression Models Comparing General Conceptual Ability at Age 6 Years Between Children of Women With Epilepsy and Healthy Women

Variable	Coefficient (95% CI)	p-value
Cohort Group		
- Healthy Women	Reference	—
**- Women with Epilepsy**	**1.31 (-2.30, 4.92)**	**0.48**
Breastfeeding Duration		
- No Breastfeeding	Reference	—
- 0 < Breastfeeding < 6m	-1.58 (-5.98, 2.83)	0.48
- Breastfeeding >= 6m	-1.19 (-5.41, 3.04)	0.58
Maternal IQ	0.34 (0.20, 0.48)	< .001
Maternal Education		
- No College Degree	Reference	—
- College Degree (Not Advanced)	4.30 (0.22, 8.38)	0.04
- College Degree (Advanced)	8.23 (3.35, 13.11)	0.001
Mean Beck Anxiety Inventory during pregnancy	-0.32 (-0.61, -0.03)	0.03
Child's sex (Male vs. Female)	-3.67 (-6.56, -0.78)	0.01

Breastfeeding duration and cohort group were forced into all steps of the modeling process. Abbreviations: CI, confidence interval; IQ, intelligence quotient.

eTable 10. Multivariable Regression Models Comparing Autism Risk Outcomes at Age 6 Years Between Children of Women With Epilepsy and Healthy Women

Variable	Coefficient (95% CI)	p-value
Cohort Group		
- Healthy Women	Reference	—
**- Women with Epilepsy**	**-0.32 (-2.08, 1.44)**	**0.72**
Breastfeeding Duration		
- No Breastfeeding	Reference	—
- 0 < Breastfeeding < 6m	0.20 (-1.40, 1.80)	0.81
- Breastfeeding >= 6m	-1.06 (-2.86, 0.74)	0.25
Child's sex (Male vs. Female)	2.22 (0.13, 4.31)	0.04

Breastfeeding duration and cohort group were forced into all steps of the modeling process. Abbreviations: CI, confidence interval; IQ, intelligence quotient.

© 2026 Li Y et al. *JAMA Network Open*.

eTable 11. Association Between Breastfeeding Duration and Adaptive Behavior Subdomain Outcomes at Age 6 Years in Children Born to Women With Epilepsy

Variable	Coefficient (95% CI)	p-value
**---ABAS-3 Practical Domain Outcome---**		
**Breastfeeding Duration**		
**- No Breastfeeding**	*Reference*	—
**- 0 < Breastfeeding < 6m**	2.49 (-1.19, 6.17)	0.18
**- Breastfeeding ≥ 6m**	**5.27 (1.84, 8.70)**	**< .01**
Mean Perceived Stress Scale post-birth	-0.42 (-0.64, -0.20)	< .01
Maternal age	-0.32 (-0.60, -0.04)	0.03
Birthweight (kg)	3.27 (0.93, 5.60)	< .01
History of Depression (Yes vs. No)	-4.05 (-7.87, -0.22)	0.04
**---ABAS-3 Concept Domain Outcome---**		
**Breastfeeding Duration**		
**- No Breastfeeding**	*Reference*	—
**- 0 < Breastfeeding < 6m**	1.69 (-1.83, 5.21)	0.35
**- Breastfeeding ≥ 6m**	**3.50 (0.28, 6.71)**	**0.03**
Mean Perceived Stress Scale post-birth[†]	-0.42 (-0.62, -0.22)	< .01
Maternal Education		
- No College Degree	*Reference*	—
- College Degree (Not Advanced)	1.39 (-1.83, 4.62)	0.40
- College Degree (Advanced)	4.41 (0.73, 8.08)	0.02
**---ABAS-3 Social Domain Outcome---**		
**Breastfeeding Duration**		
**- No Breastfeeding**	*Reference*	—
**- 0 < Breastfeeding < 6m**	1.68 (-1.44, 4.80)	0.29
**- Breastfeeding ≥ 6m**	2.14 (-0.74, 5.02)	0.14
Mean Perceived Stress Scale post-birth	-0.55 (-0.74, -0.37)	< .01
Maternal age	-0.44 (-0.69, -0.19)	< .01
Birthweight (kg)	3.07 (1.11, 5.03)	< .01
Children’s race		
- White	*Reference*	—
- Black	11.65 (5.88, 17.42)	< .01
- Others/Unknown	-0.09 (-3.51, 3.34)	0.96
Maternal Education		
- No College Degree	*Reference*	—
- College Degree (Not Advanced)	0.54 (-2.36, 3.44)	0.71
- College Degree (Advanced)	6.08 (2.69, 9.47)	< .01
Children's Sex (Male vs. Female)	-4.15 (-6.47, -1.83)	< .01

Breastfeeding duration was selected *a priori* for inclusion in the models.
Abbreviations: ABAS-3, Adaptive Behavior Assessment System, Third Edition; CI, confidence interval.

© 2026 Li Y et al. *JAMA Network Open*.

eTable 12. Association Between Breastfeeding Duration and Nonverbal Ability Outcomes at Age 6 Years in Children Born to Women With Epilepsy

Variable	Coefficient (95% CI)	p-value
**--- Nonverbal Ability ---**		
**Breastfeeding Duration**		
**- No Breastfeeding (Reference)**	*Reference*	—
**- 0 < Breastfeeding < 6m**	0.30 (-3.01, 3.61)	0.86
**- Breastfeeding ≥ 6m**	0.59 (-2.52, 3.70)	0.71
Maternal IQ	0.29 (0.17, 0.41)	< .01
Maternal Education		
- No College Degree	*Reference*	—
- College Degree (Not Advanced)	3.59 (0.51, 6.67)	0.02
- College Degree (Advanced)	6.25 (2.45, 10.05)	< .01

Multivariable linear regression models were used to predict nonverbal ability outcomes, measured by the Differential Ability Scales, Second Edition (DAS-II) Special Nonverbal Cluster score.
Breastfeeding duration was selected *a priori* for inclusion in the models.
Abbreviations: CI, confidence interval.

© 2026 Li Y et al. *JAMA Network Open*.

eTable 13. Association Between Breastfeeding Duration and General Cognitive Ability at Age 6 Years in Children Born to Women With Epilepsy at Age 6 Years

Variable	Coefficient (95% CI)	p-value
**--- General Cognitive Ability ---**		
**Breastfeeding Duration**		
**- No Breastfeeding (Reference)**	*Reference*	—
**- 0 < Breastfeeding < 6m**	0.42 (-3.21, 4.04)	0.82
**- Breastfeeding ≥ 6m**	0.42 (-3.00, 3.85)	0.81
Maternal IQ	0.36 (0.23, 0.49)	< .01
Maternal Education		
- No College Degree	*Reference*	—
- College Degree (Not Advanced)	5.52 (2.12, 8.91)	< .01
- College Degree (Advanced)	9.64 (5.45, 13.83)	< .01

Multivariable linear regression models were used to predict general cognitive function, measured by the General Conceptual Ability (GCA) score from the Differential Ability Scales, Second Edition (DAS-II). Breastfeeding duration was selected *a priori* for inclusion in the models.
Abbreviations: CI, confidence interval.

© 2026 Li Y et al. *JAMA Network Open*.

eTable 14. Association Between Breastfeeding Duration and Risk of Autism Traits at Age 6 Years in Children Born to Women With Epilepsy

Variable	Odds Ratio (95% CI)	P-Value
**---SRS-2 Autism At-Risk (Yes/No) [*]---**		
**Breastfeeding Duration**		
**- No Breastfeeding (Reference)**	*Reference*	—
**- 0 < Breastfeeding < 6m**	1.51 (0.38-6.00)	0.56
**- Breastfeeding ≥ 6m**	0.19 (0.02-1.71)	0.14
Children's Sex (Male vs. Female)	10.12 (1.26-81.60)	0.03

Multivariable logistic regression was used to estimate associations. Breastfeeding duration was selected *a priori* for inclusion in the models. Data are presented as Odds Ratios (OR) with 95% confidence intervals (CIs).

[*]Autism risk was defined as a binary outcome based on the Social Responsiveness Scale, Second Edition (SRS-2), where a T-score of ≥70 indicates "At-Risk" status.

Abbreviations: SRS-2, Social Responsiveness Scale, Second Edition.

© 2026 Li Y et al. *JAMA Network Open*.

eTable 15. Analysis of the Interaction Between Maternal Epilepsy Status and Breastfeeding Duration on Neurodevelopmental Outcomes at Age 6 Years in Children Born to Women With Epilepsy

Outcome	Cohort	N	< 6 Months	N	≥ 6 months	Interaction p-value*
**Primary outcomes:**						
--- Language ---						
Verbal Index Score#	HW	41	102.45 ± 1.25	60	111.32 ± 0.72	0.10
	WWE	191	104.76 ± 0.51	131	108.49 ± 0.50	
--- Behavioral (Composites) ---						
ABAS-3 GAC†	HW	41	100.97 ± 0.76	60	102.36 ± 0.43	0.56
	WWE	191	101.75 ± 0.32	131	101.93 ± 0.36	
**Secondary outcome:**						
--- Nonverbal Ability ---						
DAS-2 Nonverbal Cluster Score‡	HW	41	103.60 ± 1.21	60	106.17 ± 0.73	0.74
	WWE	191	105.01 ± 0.46	131	104.97 ± 0.57	
--- Behavioral Subdomain ---						
ABAS-3 Conceptual Domain§	HW	41	103.36 ± 0.49	60	103.56 ± 0.33	0.48
	WWE	191	99.88 ± 0.25	131	103.02 ± 0.25	
ABAS-3 Practical Domain‖	HW	41	100.20 ± 0.87	60	101.45 ± 0.58	0.41
	WWE	191	101.04 ± 0.36	131	101.14 ± 0.39	
ABAS-3 Social Domain¶	HW	41	104.70 ± 0.69	60	104.53 ± 0.41	0.76
	WWE	191	103.70 ± 0.32	131	105.17 ± 0.31	
--- General Cognitive Ability ---						
DAS-GCA^	HW	41	104.93 ± 1.20	60	106.30 ± 1.02	0.91
	WWE	191	105.21 ± 0.67	131	106.50 ± 0.67	
--- Autism Risk ---						
SRS-2 Autism At-Risk (Yes/No) &	HW	27	1 (3.7%)	57	2 (3.5%)	0.25
	WWE	146	8 (5.5%)	122	1 (0.8%)	

Data are presented as adjusted Mean ± Standard Error (SE) for continuous outcomes and N (%) for the categorical outcome, stratified by maternal epilepsy status (Healthy Women [HW] vs. Women with Epilepsy [WWE]) and breastfeeding duration (< 6 months vs. ≥ 6 months). For each outcome, a separate multivariable regression model (linear for continuous, logistic for categorical) was constructed that included a statistical interaction term between cohort and breastfeeding status. Covariates for each model were selected from a predefined list using an automated bidirectional stepwise procedure (inclusion criteria, P less than .05; exclusion criteria, P greater than .10).

*The interaction P value (Maternal Epilepsy status x Breastfeeding Status) tests whether the association between breastfeeding duration and child outcome differs between HW and WWE cohorts; P greater than 0.05 indicates that the effect of breastfeeding is consistent across both cohorts.

© 2026 Li Y et al. *JAMA Network Open*.

Final covariates included in adjusted models were as follows:
[#] Adjusted scores for the Verbal Index Score were controlled for maternal IQ, maternal college degree, maternal age, child sex, pregnancy planning status, and child ethnicity.
[†] Adjusted scores for the ABAS-3 GAC were controlled for maternal postnatal Perceived Stress Scale score, childbirth weight, and maternal age.
[‡] Adjusted scores for the DAS-2 Nonverbal Cluster Score were controlled for maternal IQ, maternal college degree, first-trimester folate supplementation category, and maternal history of depression.
[§] Adjusted scores for the ABAS-3 Conceptual Domain were controlled for maternal postnatal Perceived Stress Scale score and maternal college degree.
[‖] Adjusted scores for the ABAS-3 Practical Domain were controlled for maternal postnatal Perceived Stress Scale score, childbirth weight, maternal age, and child race.
[¶] Adjusted scores for the ABAS-3 Social Domain were controlled for maternal postnatal Perceived Stress Scale score, child sex, and maternal employment status.
[^] Adjusted scores for the DAS-GCA were controlled for maternal IQ, maternal college degree, pregnancy planning status, child sex, childbirth weight, and first-trimester folate supplementation.
[&] Adjusted scores for the SRS-2 Autism At-Risk were controlled for child sex and maternal postnatal Pittsburgh Sleep Quality Index score.

Abbreviations: ABAS-3, Adaptive Behavior Assessment System, Third Edition; DAS, Differential Ability Scales, Second Edition; GAC, General Adaptive Composite; GCA, General Conceptual Ability; HW, healthy women; SE, standard error; SRS-2, Social Responsiveness Scale, Second Edition; WWE, women with epilepsy.

© 2026 Li Y et al. *JAMA Network Open*.

eTable 16. Association of Breastfeeding Duration With Child Language and Behavioral Outcomes at Age 6 Years in Children of Healthy Women

Variable	Coefficient (95% CI)	P Value
**Language Outcome (Verbal Index Score)**		
**Breastfeeding Duration**		
**No breastfeeding**	*Reference*	—
**>0 to <6 mo**	0.65 (−7.03 to 8.34)	0.87
**≥6 mo**	6.42 (−0.02 to 12.86)	0.05
Maternal IQ	0.32 (0.15 to 0.48)	<.001
Maternal age (yrs)	0.63 (0.22 to 1.04)	0.003
Child sex, male vs female	−6.02 (−10.17 to −1.88)	0.004
Average folate supplement during the first trimester		
0 mg	*Reference*	—
>0-0.4 mg	11.18 (1.87 to 20.48)	0.02
>0.4-1.0 mg	13.37 (5.39 to 21.35)	<.001
>1.0-4.0 mg	10.63 (1.36 to 19.90)	0.03
≥4 mg	−0.00 (−0.00 to 0.00)	0.92
**Adaptive Behavioral Outcome (ABAS-3 General Adaptive Composite Score)**		
**Breastfeeding duration**		
**No breastfeeding**	*Reference*	—
**>0 to <6 mo**	−3.47 (−12.06 to 5.11)	0.42
**≥6 mo**	−0.15 (−7.26 to 6.97)	0.97
Child race		
White	*Reference*	—
Black or African American	12.43 (4.28 to 20.58)	<.001
Other/unknown	5.39 (−0.35 to 11.13)	0.07

© 2026 Li Y et al. *JAMA Network Open*.

eTable 17. Association of Breastfeeding Duration With Child Nonverbal Ability, General Cognitive Ability, and Behavioral Subdomains at Age 6 Years in Children of Healthy Women

Outcome	No BF	BF >0 to <6 m	P Value	BF ≥6 m	P Value
--- Nonverbal Ability ---					
DAS-2 Nonverbal Cluster Score[‡]	—	−7.03 (−17.17 to 3.12)	0.17	−5.35 (−14.20 to 3.49)	0.23
--- General Cognitive Ability ---					
DAS-GCA[^]	—	−0.26 (−10.10 to 9.58)	0.96	3.92 (−4.63 to 12.48)	0.36
--- Behavioral Subdomain ---					
ABAS-3 Practical Domain[‖]	—	−1.02 (−10.19 to 8.16)	0.83	2.70 (−4.99 to 10.39)	0.49
ABAS-3 Conceptual Domain[§]	—	−5.54 (−14.23 to 3.14)	0.21	−0.83 (−8.02 to 6.37)	0.82
ABAS-3 Social Domain[¶]	—	−7.06 (−15.96 to 1.84)	0.12	−2.48 (−9.85 to 4.90)	0.51

Values represent adjusted coefficients (95% CI) from separate multivariable linear regression models for each outcome. Breastfeeding duration categories: no breastfeeding (reference), >0 to <6 months, and ≥6 months. Coefficients indicate the mean difference in outcome score compared with the no breastfeeding reference group. (—) indicates reference category.
[‡]DAS-II Nonverbal Index: Adjusted for maternal education level.
[^]DAS-II General Conceptual Ability: Adjusted for child sex (male vs female [reference]), household income, average folate supplementation during first trimester, maternal IQ, and mean Beck Anxiety Inventory score during postpartum period.
[‖]ABAS-3 Practical Skills: Adjusted for maternal IQ.
[§]ABAS-3 Conceptual Skills: Adjusted for child race.
[¶]ABAS-3 Social Skills: Adjusted for child race.

Abbreviations: ABAS-3, Adaptive Behavior Assessment System, Third Edition; BAI, Beck Anxiety Inventory; DAS-II, Differential Ability Scales, Second Edition. BF: breastfeeding.

© 2026 Li Y et al. *JAMA Network Open*.

eTable 18. Rate of Autism Spectrum at Risk at Age 6 Years by Breastfeeding Duration in Children of Healthy Women

Breastfeeding Duration	Total No	Children With ASD, No. (%)
No breastfeeding	11	1 (9.1)
>0 to <6 mo	15	0
≥6 mo	57	2 (3.5)
Total	83	3 (3.6)

*Abbreviation: ASD, autism spectrum disorder.
ASD classification was based on Social Responsiveness Scale, Second Edition (SRS-2) T-scores at age 6 years [cutoff at T-score ≥70]. Fisher exact test was not conducted due to small cell counts (expected frequency <5 in multiple cells).

© 2026 Li Y et al. *JAMA Network Open*.

eTable 19. Distribution of Antiseizure Medication (ASM) Use Regimens in Women With Epilepsy

ASM Regimen	Language Outcome Cohort (N=262)	Behavioral Outcome Cohort (N=261)
**Monotherapy**		
Lamotrigine	91 (34.7%)	92 (35.2%)
Levetiracetam	72 (27.5%)	72 (27.6%)
Other Monotherapy	35 (13.4%)	35 (13.4%)
- Oxcarbazepine	11 (4.2%)	12 (4.6%)
- Zonisamide	9 (3.4%)	8 (3.1%)
- Carbamazepine	7 (2.7%)	6 (2.3%)
- Topiramate	4 (1.5%)	5 (1.9%)
- Lacosamide	2 (0.8%)	2 (0.8%)
- Valproic Acid	1 (0.4%)	1 (0.4%)
- Phenobarbital	1 (0.4%)	1 (0.4%)
**Polytherapy**	51 (19.5%)	51 (19.5%)

© 2026 Li Y et al. *JAMA Network Open*.

eTable 20. Neurodevelopmental Outcomes at Age 6 Years in Children of WWE Stratified by Antiseizure Medication (Monotherapy vs Polytherapy) and Breastfeeding Duration

Group	Unadjusted Median (IQR)	n	P Value	Adjusted Median (IQR)	n	P Value
**Verbal Language Outcome (Verbal Index Score)**						
**Monotherapy**						
No BF	107.14 (101.00-114.86)	57	[Reference]	106.47 (102.94-116.17)	57	[Reference]
0<BF <6 months	110.14 (104.50-115.78)	55	0.34	109.44 (102.68-113.79)	55	0.96
BF ≥6 months	109.77 (103.25-118.47)	104	0.27	108.37 (102.54-114.20)	104	0.70
**Polytherapy**						
No BF	98.29 (84.00-101.67)	25	[Reference]	106.48 (92.90-109.54)	25	[Reference]
0<BF <6 months	104.64 (100.89-108.79)	24	<.01	105.77 (102.34-110.42)	24	0.60
BF ≥6 months	106.42 (96.25-114.25)	18	<.01	103.56 (97.68-112.07)	18	0.56
**Adaptive Behavioral Outcome (ABAS-GAC Score)**						
**Monotherapy**						
No BF	99.00 (92.00-108.00)	53	[Reference]	98.83 (89.69-105.68)	53	[Reference]
0<BF <6 months	104.00 (95.00-112.00)	57	0.04	100.90 (97.20-109.14)	57	0.07
BF ≥6 months	104.00 (95.00-112.00)	105	**<.01**	103.38 (96.07-112.09)	105	**<.01**
**Polytherapy**						
No BF	98.00 (87.50-106.50)	27	[Reference]	101.45 (90.95-110.32)	27	[Reference]
0<BF <6 months	98.00 (92.25-103.00)	22	0.79	97.35 (94.54-107.28)	22	0.83
BF ≥6 months	106.00 (94.00-112.00)	17	0.28	102.74 (97.34-111.91)	17	0.39

Data are presented as median (interquartile range [IQR]). The cohort is stratified by antiseizure medication (ASM) regimen: Monotherapy vs Polytherapy. *P* values indicate post hoc pairwise comparisons between the specified breastfeeding duration group (0-6 months or ≥6 months) and the no breastfeeding reference group, calculated using the Mann-Whitney *U* test. Verbal language outcome (VIS score) was adjusted for maternal IQ, maternal education, child ethnicity, and prenatal anxiety. Adaptive behavioral outcome (ABAS-GAC scores) was adjusted for maternal age, maternal history of depression, postpartum stress, and birth weight.

Abbreviations: BF, breastfeeding; VIS, Verbal Index Score; ABAS-3, Adaptive Behavior Assessment System, Third Edition; GAC, General Adaptive Composite.

eTable 21. Association Between Breastfeeding Duration and Autism Risk at Age 6 Years in Children of Women With Epilepsy, Stratified by Antiseizure Medication Regimen or Types

© 2026 Li Y et al. *JAMA Network Open*.

Subgroup	Breastfeeding Duration	Events/Total, No. (%)	Adjusted OR (95% CI)	P Value
**Specific ASM Regimens**				
Lamotrigine	No breastfeeding	1/29 (3.4)	1 [Reference]	---
	0-6 months	1/25 (4.0)	NC	NC
	≥6 months	0/49 (0)	NC	NC
Levetiracetam	No breastfeeding	1/31 (3.2)	1 [Reference]	---
	0-6 months	2/16 (12.5)	2.13 (0.16-27.61)	0.56
	≥6 months	1/42 (2.4)	0.35 (0.02-6.24)	0.48
Other monotherapy	No breastfeeding	0/18 (0)	1 [Reference]	---
	0-6 months	2/16 (12.5)	NC	NC
	≥6 months	0/19 (0)	NC	NC
Polytherapy	No breastfeeding	1/34 (2.9)	1 [Reference]	---
	0-6 months	0/22 (0)	NC	NC
	≥6 months	0/20 (0)	NC	NC
**Aggregated Analysis**				
Monotherapy	No breastfeeding	2/78 (2.6)	1 [Reference]	---
	0-6 months	5/57 (8.8)	2.61 (0.47-14.66)	0.28
	≥6 months	1/110 (0.9)	0.27 (0.02-3.06)	0.29
Polytherapy	No breastfeeding	1/34 (2.9)	1 [Reference]	---
	0-6 months	0/22 (0)	NC	NC
	≥6 months	0/20 (0)	NC	NC

Data are presented as number of events/total number of participants (percentage). Odds ratios were adjusted for children's sex.
The reference category for all comparisons was no breastfeeding within each antiseizure medication subgroup.
NC indicates not calculated owing to zero events in 1 or more exposure categories, precluding reliable statistical estimation.
Abbreviation: ASM: anti-seizure medications; OR, odds ratio.

© 2026 Li Y et al. *JAMA Network Open*.

eTable 22. Sensitivity Analysis of the Association Between Breastfeeding Duration and Verbal Index Score at Age 6 Years Using AIC-Optimized Stepwise Regression

Variables	Estimate (95% CI)	p-value
Breastfeeding Duration		
- No Breastfeeding	Reference	—
- 0 < Breastfeeding < 6m	0.27 (-3.54, 4.09)	0.89
- Breastfeeding >= 6m	-0.30 (-3.97, 3.38)	0.88
Maternal IQ	0.28 (0.14, 0.43)	< .001
ASM Monotherapy/Polytherapy Subgroup		
- Lamotrigine Monotherapy	Reference	—
- Levetiracetam Monotherapy	-3.73 (-7.18, -0.28)	0.03
- Other Monotherapy	2.23 (-1.97, 6.42)	0.30
- Polytherapy	-3.37 (-7.31, 0.57)	0.09
Maternal Education		
- No College Degree	Reference	—
- College Degree (Not Advanced)	2.27 (-1.57, 6.11)	0.25
- College Degree (Advanced)	5.21 (0.59, 9.83)	0.03
History of Depression (Yes vs. No)		
- No	Reference	—
- Yes	-5.27 (-9.25, -1.29)	0.01
Children's race		
- White	Reference	—
- Black	8.23 (2.11, 14.34)	0.01
- Others/Unknown	-0.31 (-4.67, 4.05)	0.89
PSQI during pregnancy	-0.64 (-1.15, -0.13)	0.01
Child Ethnicity		
- Hispanic or Latino	Reference	—
- Non-Hispanic or Non-Latino	5.49 (1.14, 9.83)	0.01
Child's Sex – Male		
- Female	Reference	—
- Male	-2.40 (-5.22, 0.42)	0.10

Abbreviations: AIC, Akaike Information Criterion; ASM, antiseizure medication; CI, confidence interval; PSQI, Pittsburgh Sleep Quality Index; VIS, Verbal Index Score.
Model Specifications: The optimal model was selected utilizing a bidirectional (forward and backward) stepwise regression algorithm minimizing the Akaike Information Criterion (AIC). Candidate covariates were constrained to a Variance Inflation Factor (VIF) < 4 to prevent multicollinearity. The null model forced the retention of breastfeeding duration and ASM exposure to ensure evaluation of the primary exposure. The AIC-optimized model demonstrated an improved fit compared to the null model (AIC reduction of 52.52).

eTable 23. Sensitivity Analysis of the Association Between Breastfeeding Duration and Adaptive Behavior at Age 6 Years Using AIC-Optimized Stepwise Regression

Variables	Estimate (95% CI)	p-value
Breastfeeding Duration		

© 2026 Li Y et al. *JAMA Network Open*.

- No Breastfeeding	Reference	—
- 0 < Breastfeeding < 6m	1.33 (-2.61, 5.27)	0.51
- Breastfeeding >= 6m	4.45 (0.86, 8.04)	0.02
Mean Perceived Stress Scale post-birth	-0.46 (-0.70, -0.23)	< .001
Mother's Age at Enrollment	-0.71 (-1.04, -0.38)	< .001
Children's race		
- White	Reference	—
- Black	14.33 (7.66, 21.00)	< .001
- Others/Unknown	-0.22 (-4.59, 4.14)	0.92
Birthweight (kg)	6.50 (3.22, 9.78)	< .001
Maternal Education		
- No College Degree	Reference	—
- College Degree (Not Advanced)	0.33 (-3.45, 4.12)	0.86
- College Degree (Advanced)	4.70 (0.35, 9.05)	0.03
History of Depression (Yes vs. No)		
- No	Reference	—
- Yes	-4.77 (-8.87, -0.67)	0.02
Child's Sex – Male		
- Female	Reference	—
- Male	-3.19 (-6.20, -0.19)	0.04
WGA at Birth	-1.04 (-2.07, -0.01)	0.048

Abbreviations: ABAS-3 GAC, Adaptive Behavior Assessment System–Third Edition General Adaptive Composite; AIC, Akaike Information Criterion; CI, confidence interval; PSS, Perceived Stress Scale; WGA, weeks of gestational age.

Model Specifications: The optimal model was selected utilizing a bidirectional (forward and backward) stepwise regression algorithm minimizing the Akaike Information Criterion (AIC). Candidate covariates were constrained to a Variance Inflation Factor (VIF) < 4 to prevent multicollinearity. The null model forced the retention of breastfeeding duration to ensure evaluation of the primary exposure. The AIC-optimized model demonstrated an improved fit compared to the null model (AIC reduction of 38.58).

© 2026 Li Y et al. *JAMA Network Open*.

eTable 24. Sensitivity Analysis of Primary Outcomes Using Propensity Score Approach

Outcome and Comparison	Unadjusted Estimate (95% CI)	P Value	Propensity Score–Adjusted Estimate (95% CI)	P Value
Analysis 1: Cohort Comparison (WWE vs HW)				
Language (Verbal Index Score)	−1.44 (−4.48 to 1.60)	0.35	0.12 (−2.54 to 2.78)	0.93
Behavior (ABAS-3 GAC)	−0.58 (−3.50 to 2.35)	0.7	1.18 (−1.74 to 4.10)	0.43
Analysis 2: Breastfeeding Duration in WWE				
Language (Verbal Index Score)				
BF>0 to <6 mo vs no BF	4.91 (0.85 to 8.97)	0.02	0.36 (−3.44 to 4.17)	0.85
BF≥6 mo vs no BF	6.47 (2.77 to 10.16)	<.001	0.19 (−3.37 to 3.75)	0.92
Behavior (ABAS-3 GAC)				
BF>0 to <6 mo vs no BF	1.59 (−2.44 to 5.62)	0.44	1.10 (−2.67 to 4.87)	0.57
BF≥6 mo vs no BF	3.21 (−0.47 to 6.89)	0.09	3.60 (0.27 to 6.93)	0.03

Abbreviations: ABAS-3 GAC, Adaptive Behavior Assessment System, Third Edition General Adaptive Composite; CI, confidence interval; HW, healthy women; WWE, women with epilepsy.

© 2026 Li Y et al. *JAMA Network Open*.

eAppendix. Trial Protocol and Statistical Analysis Plan

Maternal Outcomes and Neurodevelopmental Effects of Antiepileptic Drugs *(MONEAD)*

Principal Investigators:

Kimford J. Meador, M.D.
Stanford University Palo
Alto, California
&

Page B. Pennell, M.D.
Harvard Brigham & Women's Hospital
Boston, Massachusetts

Data and Statistical Center PI:
Abigail Matthews, Ph.D. The
Emmes Corporation
Rockville, Maryland

Supported by:

The National Institute of Neurological Disorders and Stroke (NINDS) & Eunice Kennedy Shriver National Institute of Child Health and Human Development (NICHD)

Grant #1U01-NS038455

Version 9

February 01 2021

© 2026 Li Y et al. *JAMA Network Open*.

TABLE OF CONTENTS

Page

LIST OF ABBREVIATIONS .... **III**
SYNOPSIS **1**
1. STUDY OBJECTIVES **2**
1.1. PRIMARY OBJECTIVES 2
1.1.1. Neurodevelopment 2
Assess the effects of fetal antiepileptic drug (AED) exposure on cognitive and behavioral outcomes in children of WWE. 2
1.1.2 Pharmacokinetics 2
1.2. SECONDARY OBJECTIVES 2
1.2.1. Neurodevelopment 2
1.3. EXPLORATORY OBJECTIVES 2
1.3.1. Effects on Cerebral Lateralization (i.e. Language/Visual Abilities and Handedness) and Creativity 2
1.3.2. Determine the effects of primary language other than English on WASI-2 performance. 2
2. Background 2
2.1 Rationale 2
2.1.1 STUDY DESIGN 3
2.1.2 Expected Impact of Results on Medical Care and Clinical Research 3
4. SELECTION AND ENROLLMENT OF SUBJECTS **5**
4.2 EXCLUSION CRITERIA 5
4.3 STUDY ENROLLMENT PROCEDURES 5
5. MEDIATIONS **6**
5.1 MATERNAL DEPRESSION AND SUICIDALITY 6
5.2 CHILD DEVELOPMENTAL/COGNITIVE DELAY 7
6. EVALUATIONS **9**
6.1 ON-STUDY EVALUATIONS 9
6.2 DESCRIPTIONS OF STUDY EVALUATIONS 9
6.2.1 Informed Consent 10
6.2.2 Clinical Assessments 10
6.2.3 Cognitive/Emotional/Behavioral Evaluations 10
7. MANAGEMENT OF ADVERSE OUTCOMES **13**
7.1 ADVERSE EVENT REPORTING 13
7.1.1 Adverse Event Reporting in the Study Database 13
7.1.2 Serious Adverse Event Definition 13
7.1.3 Reporting Serious Adverse Events 14
7.1.4 Congenital Anomalies and Developmental Delays 14

© 2026 Li Y et al. *JAMA Network Open*.

7.2 REPORTING TO SITE IRBS 14

TABLE OF CONTENTS *(Continued)*

8 STATISTICAL CONSIDERATIONS 15
8.1 GENERAL DESIGN ISSUES 15
8.2 OUTCOMES 15
8.2.1 Primary outcomes 15
8.2.2 Secondary Outcomes 15
8.2.3 Sample Size and Power Considerations 15
8.3 DATA MONITORING 19
8.4 STATISTICAL ANALYSES 19
9 DATA COLLECTION, SITE MONITORING, AND ADVERSE EXPERIENCE REPORTING 20
9.1 RECORDS TO BE KEPT 20
9.2 ROLE OF DATA MANAGEMENT 21
9.3 QUALITY ASSURANCE 22
9.4 ADVERSE EXPERIENCE REPORTING 22
9.5 REPORTING TO SITE IRBS 22
10 HUMAN SUBJECTS 22
10.1 INSTITUTIONAL REVIEW BOARD (IRB) REVIEW AND INFORMED CONSENT 22
10.2 SUBJECT CONFIDENTIALITY 23
10.3 STUDY MODIFICATION/DISCONTINUATION 23
11 PUBLICATION OF RESEARCH FINDINGS 23
ROLE AND RESPONSIBILITIES 23
12 REFERENCES 25
APPENDICES 29
APPENDIX A: PSYCHIATRIC SYMPTOM ONSET AND/OR WORSENING/DECOMPENSATION OVER COURSE OF STUDY PARTICIPATION 30
APPENDIX B: CLINICAL AND LABORATORY EVALUATIONS SCHEDULE 32
Error! Bookmark not defined.
APPENDIX C: CHILD NEUROPSYCHOLOGICAL REFERRAL REQUIREMENTS 33

© 2026 Li Y et al. *JAMA Network Open*.

LIST OF ABBREVIATIONS

Abbreviation	Term
AAG	Alpha Acid Glycoprotein
AAN	American Academy of Neurology
ABAS-III	Adaptive Behavioral Assessment System-III
ABL	Anticonvulsant Blood Level
ACOG	American College of Obstetrics & Gynecology
AED	Anti-epileptic drug
ANCOVA	Analysis of Co-Variance
ANOVA	Analysis of Variance
APGAR	Activity, Pulse, Grimace, Appearance, Respiration
BAI	Beck Anxiety Inventory
BASC 2	Behavioral Assessment Scales for Children 2 (Parent and Teacher)
BDI 2	Beck Depression Inventory 2
BVMI6	Beery Buktenica Developmental Test of Visual-Motor Integration 6
BLOCK	Block 2005 Food Frequency Questionnaire
Bp	Blood Pressure
BRIEFP	Behavior Rating Inventory of Executive Functioning Preschool
BRIEF 2	Behavior Rating Inventory of Executive Functioning 2
BSID-III	Bayley Scales of Infant and Toddler Development-III
BWH	Brigham & Women's Hospital
CBZ	Carbamazepine/Tegretol
CDC	Centers for Disease Control
CDMS	Clinical Data Management System
CMS	Children's Memory Scale
CFR	Code of Federal Regulations
CPS	Complex-Partial Seizures
CRF	Clinical Record Forms
DAS-II	Differential Abilities Scale-II
DENVER PDQ II	Denver Prescreening Developmental Questionnaire II
DSC	Data and Statistical Center
DSM-IV	Diagnostic & Statistical Manual of Mental Disorders
EDTA	Ethylenediaminetetraacetic acid
EEG	Electroencephalograph
EOWVPT4	Expressive One Word Vocabulary Picture Test - 4
EPDS	Edinburgh Postnatal Depression Scale
FAD	Modified McMaster Family Assessment Device
FDA	Food and Drug Administration
FSR	Financial Status Report
GARS 3	Gilliam Autism Rating Scale 3
GCA	General Conceptual Ability
GCP	Good Clinical Practices
GPB	Lafayette Grooved Pegboard
GTCS	Generalized Tonic-Clonic Seizures
GYN	Gynecology
Hct	Hematocrit
Hgb	Hemoglobin

© 2026 Li Y et al. *JAMA Network Open*.

LIST OF ABBREVIATIONS *(Continued)*

Abbreviation	Term
HPLC	High Performance Liquid Chromatography
Hx	History
ICH	International Conference on Harmonisation
IQ	Intelligence Quotient
IRB	Institutional Review Board
ITT	Intent To Treat
LC/MS	Liquid Chromatography/Mass Spectrometry
LEV	Levetiracetam/Keppra
LGA	Large Gestational Age
LTG	Lamotrigine/Lamictal
MCHAT	Modified Checklist for Autism in Toddlers
MCMC	Markov Chain Monte Carlo
MDE	Major Depressive Disorder
MOP	Manual of Procedures
NDDIE	Neurological Disorders Depression Inventory in Epilepsy
NE	Neurological Exam
NEPSY2	NEuroPSYchological Assessment 2nd edition
NINDS	National Institutes of Neurological Disorders & Stroke
NPWWE	Non-Pregnant Women With Epilepsy
OB	Obstetrics
OHRP	Office of Human Research Protections
OXC	Oxcarbazepine/Trileptal
PCP	Primary Care Physician
PE	Physical Exam
PHT	Phenytoin/Dilantin
PI	Principal Investigator
PLS-5	Preschool Language Scale -5
PK	Pharmacokinetics
PKU	Phenylketonuria
PPD	Post-Partum Depression
PPVT 4	Peabody Picture Vocabulary Test 4
PSI 4	Parenting Stress Index 4
PSS-14	Perceived Stress Scale 14
PSQI	Pittsburgh Sleep Quality Index
PW	Pregnant Women
PWWE	Pregnant Women with Epilepsy
PWWoE	Pregnant Women without Epilepsy
QA	Quality Assurance
QC	Quality Control
RCT	Randomized Clinical Trial
SAE	Serious adverse event
SCID	Structured Interview for Clinical Diagnosis Patient Edition (Jan 2010)
SD	Standard Deviation
SGA	Small for Gestational Age
+SI	Having Suicidal Ideation

© 2026 Li Y et al. *JAMA Network Open*.

LIST OF ABBREVIATIONS *(Continued)*

Abbreviation	Term
SID	Subject Identification Number
SRS2	Social Responsiveness Scale 2
SSI	Supplemental Security Income
T4	Thyroxine
TANF	Temporary Assistance for Needy Families
Tri	Trimester
TSH	Thyroid Stimulating Hormone
TTCT	Torrance Test of Creative Thinking
U/S	Ultrasound
VMI-6	Beery-Buktenica Test of Visual Motor Integration -6
VPA	Valproate/Depakote
WASI-II	Wechsler Abbreviated Scale of Intelligence- II
WISC-C	Wechsler's Intelligence Scale for Children – Coding Subtest
WRAT-4	Wide Range Achievement Test - 4
WWE	Women with epilepsy
WWoE	Women without epilepsy

© 2026 Li Y et al. *JAMA Network Open*.

SYNOPSIS

Study Title

Maternal Outcomes and Neurodevelopmental Effects of Antiepileptic Drugs

Objectives

An overall goal of this study's continuation is to establish the relationship between antiepileptic drug (AED) exposure and outcomes in the mother and child as well as describe and explain the variability in AED exposure and response.

The purpose of this observational study over the next grant cycle is to: 1) Determine the long-term effects of *in utero* AED exposure on verbal intellectual abilities and other neurobehavioral outcomes in the children of Women with epilepsy (WWE); 2) Determine if breastfeeding when taking AEDs impairs the child's ultimate verbal and other cognitive outcomes.

Design and Outcomes

This multicenter investigation will employ a prospective, observational, parallel-group, cohort design.

Outcomes for mothers in this study included: Depression. Outcomes for children will include: Neurodevelopmental outcomes measured by the Language Scale of the Bayley Scales of Infant Development-III (BSID-III) at age 2 and Verbal Index of the Differential Abilities Scale –II (DAS-2) at age 6 as well as other cognitive outcomes. Breastfeeding outcomes measured by the Language Scale of the BSID-III at age 2 years and Verbal Index at age 6 years.

Interventions and Duration

This study will quantitate and delineate contribution of AED exposures and other contributing factors, account for variability, and identify significant outcome risks. This observational study will not employ any interventions.

Children of the pregnant women will ultimately be followed until they are 6 years old.

Sample Size and Population

Subjects include mother/child pairs enrolled during pregnancy (including 105 healthy women and 351 women with epilepsy) and Children are classified into 4 groups with healthy mothers or mothers diagnosed with epilepsy on AED monotherapy, polytherapy, or no AED treatment. Group proportions for key demographic factors such as age, race, education, and socioeconomic status were monitored and balanced.

© 2026 Li Y et al. *JAMA Network Open*.

1. STUDY OBJECTIVES

1.1. Primary Objectives

1.1.1. Neurodevelopment

Assess the effects of fetal antiepileptic drug (AED) exposure on cognitive and behavioral outcomes in children of WWE.

1.1.2 *Pharmacokinetics*

Use population pharmacokinetic (pop PK) modeling to quantitate and explain variability in AED exposure and its relation to outcomes..

1.2. Secondary Objectives

1.2.1. Neurodevelopment

To determine if children exposed *in utero* to certain AEDs will exhibit a concentration dependent impairment in different neurobehavioral outcomes (see details in the MOP for a list of neurobehavioral outcomes).

1.3. Exploratory Objectives

1.3.1. Effects on Cerebral Lateralization (i.e. Language/Visual Abilities and Handedness) and Creativity

1.3.2. Determine the effects of primary language other than English on WASI-2 performance.

2. *Background*

2.1 Rationale

Women with epilepsy who are considering pregnancy, and the physicians caring for them, face a great deal of uncertainty about the risks and benefits of continued anti-epileptic drug (AED) use during pregnancy. Effective seizure control for the potential mother is extremely important but often difficult to maintain as a result of unpredictable pharmacokinetic changes during pregnancy. In addition, the need for total seizure control is balanced against the possibility of serious adverse outcomes for the child exposed to AED medications during gestation and/or breastfeeding. Pregnancy registries have identified that two commonly used AEDs, valproate and topiramate, are associated with increased risks to the fetus, including congenital malformations and low birth weight.[1-3] In addition, the Neurodevelopmental Effects of Antiepileptic Drugs (NEAD) Study documented a significant risk of lower IQ in 3-year old children exposed to valproate.[4] A series of practice parameters on management of pregnant women with epilepsy, issued in 2009 by the American Academy of Neurology (AAN) and the American Epilepsy Society (AES),[5] identified multiple areas of clinical management for which evidence was inconclusive or lacking. These areas include change in seizure frequency,

© 2026 Li Y et al. *JAMA Network Open*.

alterations in blood levels for each of the AEDs and if related to seizure frequency, rates of obstetrical complications, adverse perinatal outcomes, and breastfeeding.

2.1.1 Study Design

A prospective design provides assessment and statistical control of critical variables, but a prospective randomized clinical trial (RCT) is not ethical or practical for pregnancy outcomes in WWE. Randomization of WWE to AED early in the pregnancy is not ethical due to risk of seizure exacerbation in the mothers and exposure of the fetus to polytherapy during AED transition, which could increase the risk of malformations. Additionally, randomization of WWE to AED prior to pregnancy is not practical. Half of pregnancies are unplanned and WWE may have reduced fertility. The number of centers required to enroll an adequate sample would have to be markedly increased and many WWE who would never become pregnant would need to be enrolled and followed on blinded medications. More importantly, WWE who are presently well controlled would be very unlikely to enroll since there is evidence as noted in the recent AAN guidelines that WWE who are seizure free for 9-12 months prior to pregnancy are likely to remain seizure free during pregnancy. Thus, there will be an oversampling of WWE with poorly controlled epilepsy that would not be reflective of the general WWE population. Once a woman was randomized, a large percentage would be likely to fail to achieve seizure freedom, which would result in the need to change AED regimen either prior to or during pregnancy. Such changes could result in a high dropout rate or a complicated mixture of AED exposure across type of AED and across time of AED change. However, an appropriately designed observational study can provide important information to guide the care of WWE during pregnancy and improve outcomes for them and their children.

2.1.2 Expected Impact of Results on Medical Care and Clinical Research

The NEAD Study has provided important and unique information to help guide the care of WWE during pregnancy,[4] but there are numerous areas where our medical evidence to guide care of these women is absent or inadequate.[5-7] Further, prescription practices have changed at epilepsy centers since the original NEAD enrollment.[8] Ongoing pregnancy registries are designed to address the risk of congenital malformations, but are not designed to address many other unanswered questions noted by the AAN guidelines. This continuation grant will address the following: **1)** Although some AEDs can clearly impair neurodevelopment,[8,17,31,62-3] data are inadequate or absent for most AEDs.[64] The results will provide clear indications of AED risks to the immature brain (absolute to healthy pregnant women (**HPW**), relative to other AEDs, and relative to fetal AED-exposure) for the presently most commonly used AEDs in monotherapy and polytherapy, and provide sentinel data on less commonly used AEDs to direct future studies. **2)** AED exposure in the fetus via placental passage and in the child via breastfeeding are uncertain for most AEDs.[3] The proposed studies will relate neuropsychological outcomes to fetal and infant exposure, accounting for marked and variable clearance changes during pregnancy, which is critical given teratogenetic effects are related to level of exposure.[17,113] **3)** NEAD provided evidence that fetal AED

© 2026 Li Y et al. *JAMA Network Open*.

exposure can alter normal cerebral lateralization,[12,17] but additional evidence is needed. **4)** Breastfeeding has known benefits,[245] but many clinicians advise WWE not to breastfeed. The AAN concluded "clinical consequences for the newborn of ingesting AEDs via breast milk remain sorely underexplored."[3] NEAD and a Norwegian study found no effect of breastfeeding on child IQ at age 3.[11, 111] Further, we found higher age 6 IQs in children of WWE who breastfed on AEDs.[22] However, studies are limited to a few monotherapy AED regimens, and lack of mother/child AED blood levels (**ABLs**). MONEAD will provide more evidence along with additional AEDs in this regard

The potential economic impact of one finding from the NEAD Study is highlighted. The USA National Center for Health Statistics reported 4.3 million births in 2007.[49] Since 3 to 5 births per thousand are to women with epilepsy (WWE), there are 12,951 to 21,585 children born to WWE each year [(3 - 5/1000) X (4.317 million births)]. The average incremental cost for a special education student (i.e., above and beyond the cost of a regular student) is $7,500 per year in 2009 dollars.[50] Special education is required in 10-35% of AED-exposed children due to neurodevelopmental deficits.[51] Thus, special education costs for children of WWE each year could reach $680 million = (7,554 children of WWE need special education) X ($7500 special education costs / year) X (12 years of preschool, elementary and secondary school special education). When examining longer-term financial effects, each single IQ point loss is associated with a loss of income in 2009 dollars of $17,870 (range=$15,650-$21,200) based upon the pediatric lead exposure literature.[52] Based on CDC data, VPA was the 2nd most prescribed AED to WWE who were of childbearing potential from 1999 - 2006 (the last year available). VPA comprised 20% of all AED prescriptions in 2006 to WWE. Since less than half of prescriptions for VPA are for seizures or epilepsy (i.e., most for pain and psychiatric indications), the number of children exposed would be at least doubled. Based on these facts and the data presented above, we can calculate that approximately 8,634 children are exposed *in utero* to VPA each year. Since 30% of children exposed to VPA need special education,[51] the annual costs for special education in the USA each year due to VPA exposure would be $233 million dollars. Further, the loss in lifetime earnings for children exposed each year to VPA would be $1.08 billion based on a 7 IQ point drop and number of VPA exposures in 2006. Note that this is a conservative estimate given that the IQ drop for VPA ranged from 7-9 points in our age 3 data*,* 7-15 points in retrospective study, 6-12 points and 13-14 in prospective studies.[4, 18, 53, 54, 56]

Taken together, the proposed MONEAD studies will markedly increase medical evidence to direct care in WWE during pregnancy, have potential to alter care, reduce adverse outcomes, and direct future research.

3. STUDY DESIGN

This multicenter investigation will employ a prospective, observational, parallel-group, cohort design. A prospective design provides assessment and statistical control of critical variables, but a prospective randomized clinical trial (RCT) is not ethical or practical for this population (see 2.2.1). Observational studies are important to address issues that cannot be approached by RCTs.[57] Results from the ongoing MONEAD Study and multiple pregnancy registries are

© 2026 Li Y et al. *JAMA Network Open*.

evidence that such a design can provide valuable information. Assessment of outcome variables will be blinded to study group assignment related to the specific analysis (e.g., neurobehavioral assessments will be by investigators blinded to AED). Population PK employs sparse sampling analysis strategies and is particularly useful in special populations such as pregnant women where intensive sampling is not practical. This approach provides a means to estimate and identify sources of variability in PK parameters and provides quantitative and semi-quantitative guidelines for individualization/optimization of patient dosing.

4. SELECTION AND ENROLLMENT OF SUBJECTS

4.1 Inclusion Criteria

1. Current or previous participation in the MONEAD study. Women who are lost to follow-up but have consented to re-contact will be approached about returning to the project
2. Access to a telephone for phone contact
3 Child born into study.

4.2 Exclusion Criteria

1. Women who are not currently or previously part of the MONEAD study.
2. Children born to women in the study after Feb 2014
3. Concurrent participation in an experimental drug trial.

4.3 _Study Enrollment Procedures

Subjects for this study will be the same subjects currently participating in the MONEAD study. Women who are lost to followup but have consented to re-contact will be approached to rejoin the study. Subjects will be presented with the continuation informed consent by the center's principal investigator or co-investigator, allowed to read over it, and then any questions they have concerning the project will be addressed. Each subject will receive a copy of their signed informed consent, and an additional copy will be filed in their medical records. When children develop sufficient verbal skills, verbal assent will be obtained per the site's IRB standards. It is not expected that most children will obtain sufficient writing and reading skills during the course of this investigation to obtain a written informed consent. Informed consent templates provided by the lead center will be used to guide development of the site-specific consent forms. Only an IRB-approved consent form will be used to consent subjects for participation in the study. The changes in the informed consent template by the site must be approved with the MONEAD Project Manager before submission to respective IRB. The written informed consent documents will be prepared in the language(s) of the potential subject population. Before a subject's participation in the protocol, it is the investigator's responsibility to ensure that written informed consent is obtained from the subject after adequate explanation of the aims, methods, anticipated benefits, and potential hazards of the protocol. The acquisition of informed consent should be documented in the subject's records, as required by 45 CFR 46.117, and the informed consent form should be signed and personally dated by the subject and by the person

© 2026 Li Y et al. *JAMA Network Open*.

who conducted the informed consent discussion. An original signed informed consent form should be retained by the site and a signed copy of the consent form should be provided to the subject.

4.4 Teacher Inclusion and Enrollment Procedures:

Teacher must have at least 30 day's familiarity with the child.

The child's parent should contact the child's teacher obtain permission for the site to contact the teacher. If the teacher agrees, the BASCT form, study staff contact information, and consent (if applicable) will be sent to the teacher via the parents or mailed to the teacher's school address by the study staff. The teacher will sign the consent (if applicable), complete the BASCT and give it back to the mother or mail it back to the clinic. If phone consenting is not allowed, the teachers will be scheduled for a clinic visit to consent and complete the questionnaire

The teacher is not given an ID and is not considered a subject in the study. Their purpose is to provide insight into the child's behavior outside of the family unit. The only information collected is the child's ID and the date of completion. NO OTHER information should be included on the form.

5. MEDIATIONS

Although the MONEAD study is not designed or intended to influence the mother's, child's, or any participant's care or treatment, there is a moral and ethical responsibility to inform subjects when study assessments provide results that may indicate a need for interventions outside of the study setting.

5.1 Maternal Depression and Suicidality

Maternal depression is initially evaluated using the Beck Depression Inventory II (BDI-II) The majority (65%) of the mothers are anticipated to score within the normal ranges on these assessments. However, approximately 35% may score within the psychiatric symptom range.

The majority of cases will fall into the following categories:

A. Psychiatric Symptom Onset/Worsening/Decompensation over Course of Study Participation

And/or

B. Endorsement of Suicidal Ideation

The BDI II manual notes that a total score ::9 is consistent with being euthymic, 10-13 is minimal depressive symptoms, 14-19 mild, 20-28 moderate, and 229 is considered severe depressive symptoms, and item #9 queries thoughts of suicide, with a "0" response being the positive, meaning no suicidal ideation.

© 2026 Li Y et al. *JAMA Network Open*.

Sites will use the total scores of these assessments to determine psychiatric symptom onset and worsening. Suicidal ideation will be determined by Item #9 from the BDI-II. Any non-zero (0) answer to item #9 in the BDI-II will initiate use of an algorithm (see Appendix A) to determine if additional assessment or possible outside intervention is required.

An additional assessment will include the Structured Interview for Clinical Diagnosis Patient Edition January 2010 (SCID) Depression Mood Module. Each site will be trained by the Psychiatric Core in the SCID Depression Mood Module administration and scoring and the psychometric properties and interpretation of the instruments.

Sites will use the algorithms in Appendix A to determine the best course of action.

5.2 Child Developmental/Cognitive Delay

When a child's cognitive evaluation and/or physical evaluation indicates delayed or impaired development a referral recommendation should be made to the parents.

5.2.1 Early Intervention Services – Birth to 3 years of age:

The Individuals with Disabilities Education Act (IDEA) Part C states:
Any child under the age of three years who has a disability or significant developmental delays is entitled to receive Early Intervention Services. The child may qualify if he/she is diagnosed with a physical or mental condition that has a high probability of resulting in developmental delay. The same child may also qualify if they are exhibiting developmental delays, as measured by appropriate diagnostic instruments and procedures in one or more of the following areas:

- Cognitive development
- Physical development, including vision and hearing
- Communication development
- Social or emotional development
- Adaptive development

Based upon the findings of the 2 year MONEAD evaluation, the general criteria for recommending further assessment and follow-up through the child's local Early Intervention Program are scores 2 2 standard deviations below the mean of 100 (a score < 70) in one domain or two or more scores 2 1.5 standard deviations below the mean of 100 (scores < 77) in two or more domains as measured by the following BSID-III Composites: Cognitive, Language, Motor, Social-Emotional, Adaptive Behavior; or an M-CHAT: Critical score > 2 or a Total score > 3. (Please refer to section 6.2.3 for full names/basic descriptions of the assessment instruments.)

If any MONEAD child meets such assessment criteria, recommendation for appropriate therapy (occupational/physical/speech-language) and interventions should be included in the child's report and provided to the parent. In addition, with parental consent, a referral should be made for consideration of Early Intervention Services. Of course, parents have the right to pursue similar therapies or interventions privately at their own expense.

Documentation of the therapy type, start and stop dates along with assessment results will be included in the study records.

© 2026 Li Y et al. *JAMA Network Open*.

Sites will use the algorithms in Appendix C to determine specific alerting criteria and corresponding required course(s) of action.

Head Start: Children from birth to age five from families with income below the poverty line are also eligible for Head Start (preschool age children) and Early Head Start services (birth to age 3 and pregnant women). Eligibility criteria vary between states. Sites should refer to their state's Head Start site for their particular criteria. Head Start provides a learning environment to promote children's growth in language, literacy, cognition, physical development, and social and emotional development. Children from families receiving public assistance (TANF or SSI) are also eligible for these services regardless of income. Documentation of start and stop dates along with progress and assessment results will be included in the study records.

5.2.2 Preschool Special Education Services - 3 through 9 years of age:

In accordance with The Individuals with Disabilities Education Act (IDEA):
Any child between the ages of 3 through 9 years who has a disability or significant developmental delays is entitled to receive Preschool Special Education Services.
Children may qualify if they are diagnosed with a physical or mental condition that has a high probability of resulting in developmental delay. These children may also qualify if they are exhibiting developmental delays, as measured by appropriate diagnostic instruments and procedures in one or more of the following areas:

- Cognitive development
- Physical development, including vision and hearing
- Communication development
- Social or emotional development
- Adaptive development

Based upon the findings of the 3, 4.5 or 6 year MONEAD evaluations, the general criteria for recommending further assessment and follow-up through the child's local public school system Preschool Special Education Program are scores $\geq$ 2 standard deviations below the mean of 100 (a score $\leq$ 70) in one area or two or more scores $\geq$ 1.5 standard deviations below the mean of 100 (a score $\leq$ 77) in two or more of the above listed areas. (Please refer to section 6.2.3 for the full names/basic descriptions of the assessment instruments.)

If the child's 3, 4.5 or 6 year evaluation indicates delayed or impaired development a report with recommendations for appropriate therapy (occupational/physical/speech-language) and interventions should be provided to the parents. In addition, with parental consent, a referral should be made for consideration of Preschool Special Education Services. Of course, parents have the right to pursue similar therapies or interventions privately at their own expense.

Documentation of the therapy type, start and stop dates along with assessment results should be included in the study records.

Sites will use the algorithms in Appendix C to determine specific alerting criteria and corresponding required course(s) of action.

© 2026 Li Y et al. *JAMA Network Open*.

6. EVALUATIONS

6.1 On-Study Evaluations

Subject's study evaluations will occur in face-to-face meetings in the study office setting or via telephone conferences. If unable to attend the face-to-face meetings, study evaluations amenable to conversation can be administered over the phone, via skype or other type of e-communications. Copyrighted documents may be mailed to the subject to complete and return as long as they are completed within the appropriate window.

Visits 9, 10, 11 & 12 should be performed in the study office setting.

Visits 8a-c, 9a-c, 10a-e, and 11a-e evaluations are performed via teleconference with the subject.

Visit Windows

Visit windows vary according to the type of visit. All assessments, unless otherwise noted, must be completed within the following windows:

- Quarterly telephone contacts *must* occur +/- 28 days of the target date..
- Visits 9, 10, 11, & 12 should occur within 90 days after the child's birthday for both the mother and child. These assessments should not occur prior to the child's birthday.
- For children with 6YO birthdays in June & July, the Visit 12 BASC2 Teacher questionnaire can be completed during the school year proceeding their 6YO birthday no earlier than May. For example, if school lets out in May 2019, then children with birthdays between June & July 2019 can have the BASCT completed before school lets out in May 2019. ONLY CHILDREN WITH 6YO BIRTHDAYS IN JUNE & JULY AND ONLY THE BASCT ARE ELIGIBLE FOR THIS EXTENDED WINDOW.

  Children born in other months of the year (August – April) will follow the usual 90 days after the child's birthday window only AFTER THE TEACHER AND CHILD HAVE BEEN EXPOSED TO EACH OTHER FOR ONE MONTH.

6.2 Descriptions of Study Evaluations

This section provides a general description of the assessments included in the Schedule of Events tables in Appendix B. Detailed procedures with instruction for completion are included in the MOP.

© 2026 Li Y et al. *JAMA Network Open*.

6.2.1 *Informed Consent*

a. Prior to the conduct of any study procedures on any participant, IRB-authorized study site personnel must obtain legally effective informed consent from the subject or, if the subject is a minor (as defined by the site's IRB), the subject's Legally Authorized Representative.

b. The person who will initiate the informed consent process will verify that the date of consent is within the IRB approval period. The informed consent document will be provided to the prospective subject/patient in advance of the appointment where the document is to be signed whenever possible.

c. The investigator will discuss the study and review the informed consent document with the prospective subject, allowing for adequate time for discussion and questions. Once the subject/patient has shown understanding of the study and has agreed to participate in the study, both the subject/patient and the investigator will sign and date the document.

d. Once the subject/patient and investigator have signed the consent document, a copy will be provided to the subject/patient. The original consent will be kept in the study records. Study personnel shall document in the subject/patient's research record the facts of (a) provision of the fully signed informed consent to the subject/patient, and (b) that the subject/patient signed the informed consent before any research procedures were performed.

6.2.2 *Clinical Assessments*

The child will have a physical and neurological exam, including head circumference at the 2YO, 3YO, 4.5YO and 6YO visits. Child physical and neurological exams will be performed by the study clinician investigator and will include a review of systems, general appearance, head circumference, height and weight. Updates in the child's health will be collected at each visit.

Additional genetic samples to increase the amount of sample we have already collected to ensure proper quantities for possible future research projects investigating any enzymes or genetic susceptibility in the subjects that interact/work with AED metabolism and if those are related to any outcomes in humans is optional for the mothers and children. The subjects may participate in the project without participating in the genetic sampling. For those who consent, the sample will be collected via cheek swab at either Visit 11 or 12 from the mother and child.

6.2.3 *Cognitive/Emotional/Behavioral Evaluations*

a. Questionnaires

© 2026 Li Y et al. *JAMA Network Open*.

1. The Beck Depression Inventory-2 (BDI-2) is a self- reported measure of depression.

2. The Beck Anxiety Inventory (BAI) is a self-reported measure of anxiety.

3. The Perceived Stress Scale-14 item is a self-reported measure of general stress.

4. The Parenting Stress Index-4 is a self-reported, parent focused measure of stress.

5. The Behavior Assessment System for Children-2 (BASC-2) is a maternally completed questionnaire regarding her child's behavior. At V6, the teacher version of the form will be collected from a teacher familiar with the child.

6. The Behavior Rating Inventory of Executive Functioning (Preschool and version 2) (BRIEFP/BRIEF 2) are maternally completed questionnaires regarding her child's cognitive processes such as problem solving.

7. The Adaptive Behavior Assessment System-3 (ABAS-3) is a maternally completed questionnaire regarding her child's ability to respond to daily demands.

8. The Modified Checklist for Autism in Toddlers (M-CHAT) is a maternally completed questionnaire to assess her child's risk for autism spectrum disorders.

9. The Modified Edinburgh Handedness Inventory-R is a maternally report measuring the child's hand dominance.

10. The Modified McMaster Family Assessment Device (FAD) is a maternally completed questionnaire regarding family functioning.

11. Social Responsiveness Scale- 2 (SRS2) is a diagnostic assessment which identifies the presence and severity of social impairment within the autism spectrum and differentiates it from that which occurs in other disorders.

12. The Gilliam Autism Rating Scale-3 (GARS-3) is a diagnostic assessment of autism and its severity. It is collected during V10.

13. COVID Impact Questionnaire: an assessment of attitudes, perceptions and beliefs to be included as a covariate for analysis involving the mother's mood and stress questionnaire results. It is collected at either Visit 11 or 12.

© 2026 Li Y et al. *JAMA Network Open*.

b. Administered Adult Assessments

1. The Structured Clinical Interview for DSM-IV (SCID) Depression Mood Module is administered by a Project Certified Assessor and is a diagnostic assessment device to determine depressive disorders. This will only be administered if the mother's BDI-2 (see Appendix A) score is 2 10 or if there is a positive response to the suicidal ideation question #9

c. Professionally Administered Child Assessments

1. Bayley Scales of Infant and Toddler Development-3 (BSID-III) is the initial assessment of child mental and motor development. Both components are collected during V9 with the child. The motor component is repeated during V10 with the child. The Language Scale component is the primary outcome measure for 2 year olds.

2. The Differential Abilities Scale-2 (DAS-2) is an assessment of child cognitive abilities. It is collected at V10 and V12. The Verbal Index Score is the primary outcome measure for 3 year olds.

3. The Preschool Language Scale-5 (PLS-5) is an assessment of child language skills. It is collected at V1.

4. The Peabody Picture Vocabulary Test-4 (PPVT-4) is a brief administered verbally based assessment of intelligence. It is collected at V10 & V12..

5. The Beery-Buktenica Developmental Test of Visual-Motor Integration-6 is an assessment of the child's eye-hand coordination. It is collected at V10 & V12 .

6. The Torrance Test of Creative Thinking- Figural (TTCT-F) is an assessment of creative thinking. It is the single administered child assessment during V11.

7. NEuroPSYchological Assessment 2nd edition (NEPSYII) is an assessment of Attention, Executive Functioning, Language, Memory and Learning, Social Perception, Sensorimotor, and Visuospatial Processing. It is collected at V12

8. Expressive One Word Picture Vocabulary Test-4 (EOWPVT4) is an assessment of expressive vocabulary. It is collected at V12

9. Wechsler's Intelligence Scale for Children 4 (WISC4) Coding subtest is an assessment of visual-motor speed and complexity and motor coordination. It is collected at V12.

© 2026 Li Y et al. *JAMA Network Open*.

10. Children's Memory Scale (CMS) is an assessment of attention, declarative memory, and learning for children from 5 to 16 years of age. It is collected at V12.

11. Lafayette Grooved Pegboard (GPB) is an assessment of manipulative dexterity measuring performance speed in a fine motor task. It is collected at V12

12. Wide Range Achievement Test 5th edition (WRAT5) is an assessment of fundamental reading, spelling, and match skills. It is collected at V12

7. MANAGEMENT OF ADVERSE OUTCOMES

7.1 Adverse Event Reporting

For the purposes of this observational study, only serious adverse events (SAEs) will be collected. If an event is both an SAE and an outcome, it should be collected as an outcome and noted that it also represents an SAE.

7.1.1 *Adverse Event Reporting in the Study Database*

The following guidelines will be used to determine whether or not a serious adverse event is recorded in the study database.

All SAEs that occur in children will be collected for the duration of their participation in the trial. NICU admissions do not need to be submitted as SAEs.

7.1.2 *Serious Adverse Event Definition*

The term "Serious Adverse Event" (SAE) is defined in the 21 CFR 312.32 as follows: Death, a life-threatening adverse event, inpatient hospitalization or prolongation of existing hospitalization, a persistent or significant incapacity or substantial disruption of the ability to conduct normal life functions, or a congenital anomaly/birth defect. For this study, SAE collection will be confined to death, hospitalization or prolongation of hospitalization, or events requiring medical or surgical intervention due to their life-threatening nature. Important medical events that do not result in death, are not life-threatening, or do not require hospitalization may be considered serious when, based upon appropriate medical judgment, they may jeopardize the subject and may require medical or surgical intervention to prevent one of the outcomes listed in this definition. An example of such an event is allergic bronchospasm requiring intensive treatment in an emergency room or at home. An SAE will be considered "life-threatening" if, in the view of either the investigator or sponsor, its occurrence places the patient or subject at immediate risk of death. It does not include an adverse event or suspected adverse reaction that, had it occurred in a more severe form, might have caused death.

© 2026 Li Y et al. *JAMA Network Open*.

7.1.3 *Reporting Serious Adverse Events*

Adverse events that meet Serious Adverse Event (SAE) Reporting Requirements must be reported in AdvantageEDC^{SM} within 24 hours of site awareness of occurrence:

- death
- requires unplanned inpatient hospitalization or prolongation of existing hospitalization, other than those occurring for standard obstetric care
- events requiring medical or surgical intervention due to their life-threatening nature. Given the expected substantial number of cesarean-sections, these will not be counted and treated as an SAE.

In addition, any event, regardless of severity, which in the judgment of a site investigator represents a serious adverse event, may be reported on an expedited basis.

A site investigator will communicate an initial SAE report within 24 hours of site awareness of occurrence through the communication methods provided by the Data and Statistical Center (DSC): The Emmes Corporation (Rockville, MD).

Any SAE entered into the study database will generate automatic email notification to the MONEAD Medical Monitor. The investigator must submit additional information as it becomes available, using the Serious Adverse Event form in AdvantageEDC.

7.1.4 *Congenital Anomalies and Developmental Delays*

As congenital anomalies and developmental delays are a primary interest in this study, additional information about them will be collected. These adverse events should NOT be reported as an SAE for the mother, but will instead be reported as an adverse event for the child. Collection of these events will be done via a form in AdvantageEDC separate from the SAE reporting form.

- Congenital anomaly: The event is one that exists or dates from birth. The severity of the event will be graded as assessed by the investigator.
- Developmental Delay: a delay in one or more of the following areas of childhood development as measured by appropriate diagnostic instruments and standard procedures. Areas of assessment include: cognitive development; physical development, including vision and hearing; communication development; social and/or emotional development; adaptive skills including eating skills, dressing, and toileting skills, and other areas of personal responsibility.

7.2 Reporting to Site IRBs

Each site Investigator on Record is responsible for reporting adverse events to the site IRB in accordance with their IRB's requirements for expedited reporting and continuing review reporting.

© 2026 Li Y et al. *JAMA Network Open*.

8 STATISTICAL CONSIDERATIONS

8.1 General Design Issues

MONEAD is a multicenter investigation employing a prospective, observational, parallel-group, cohort design.

8.2 Outcomes

8.2.1 Primary outcomes

Depression: Rates of depression during pregnancy and post-partum as determined by screening instrument (Beck Depression Inventory-II; BDI-II), the EPDS (Edinburgh Postnatal Depression Scale) and confirmed by the Structured Clinical Interview for DSM-IV (SCID).

Neurodevelopment: Verbal intellectual ability at age 6 years is the ultimate primary outcome. It is determined by Verbal Index which is average of Word Definitions and Verbal Similarities subtests from the Differential Ability Scales-2nd ed. (DAS-II),[50] - School Age Level, Expressive One-Word Picture Vocabulary Test-4,[51] the Phonological Processing, Comprehension of Instructions and Sentence Repetition subscales from the NEPSY-II [57] and the Peabody Picture Vocabulary Test-4th ed. (PPVT-IV).[52] Children will not reach age 6 in this initial grant period, so primary outcome at age 2 will be the Language Scale from the Bayley Scales of Infant & Toddler Development (3rd ed.).[53]

Neonatal: Small for Gestational Age (SGA <10%tile).

8.2.2 Secondary Outcomes

Depression: Depression during pregnancy and post-partum will be analyzed separately. The possible contribution of confounding factors will be assessed.

Neurodevelopment: Other neurobehavioral outcomes (see the MOP for more details about neurobehavioral outcomes assessed).

Neonatal: Gestational age at delivery, birth weight Z-score (measure of growth restriction that is scaled for gestational age), head circumference, premature delivery (incidence <37 weeks, and <34 weeks), APGARs (1 & 5 minutes), Neonatal Intensive Care Unit admission and admissions >12hrs, hypoglycemia (<45), need for resuscitation, and neonatal death.

8.2.3 Sample Size and Power Considerations

The target enrollment will be approximately 451 women and children currently active in the study.

© 2026 Li Y et al. *JAMA Network Open*.

Target enrollment numbers above do not account for subject dropout. For all power calculations presented in this section, specific dropout rates were assumed for each outcome measure according to the timing of data collection for the outcome measure. For outcome measures available at the birth visit (seizure frequency relative to baseline, OB complications, small for gestational age), it was assumed that 10% of enrolled subjects would drop out. For outcome measures recorded at 9 months post-partum (seizure frequency and post-partum depression), it was assumed that 15% of enrolled subjects would drop out. For outcome measures recorded later than 9 months post-partum (neurodevelopment outcomes, IQ measures correlated with breastfeeding status), it was assumed that 20% of enrolled subjects would drop out.

Depression:

	**Depression: Rate in PWWE**			
**Rate in PWWoE**	**20%**	**25%**	**30%**	**35%**
**5%**	96.2%	99.8%	100%	100%
**6%**	92.0%	99.4%	100%	100%
**7%**	85.1%	98.3%	99.9%	100%
**8%**	77.0%	96.4%	99.7%	100%
**10%**	57.7%	88.0%	98.5%	99.9%
**12%**	37.1%	74.4%	94.9%	99.5%

The primary comparison is the rate of depression in the PWWE and PWWoE groups. Depression rates will be computed both during pregnancy and during the post-partum period (comparable time period for non-pregnant WWE). It is assumed that 15% of subjects will drop out by the end of the post-partum period (9 months after birth). Adjusting for the dropout rate gives N = 297 evaluable subjects in the PWWE group and N = 85 evaluable subjects in the NPWWE group. The power for this comparison is displayed in the table below for various assumed depression rates in the PWWE and PWWoE groups. Power was computed via simulation and Fishers Exact Test (a=0.05, two-sided). The power was above 90% for most of the assumed depression rates.

Neurodevelopment (Verbal Index Score): The primary hypothesis is that AEDs affect neurodevelopment of children exposed *in utero* compared to healthy non-exposed controls. Sample sizes are calculated to detect a between-group difference of ½ SD at the 5% significance level, comparing Verbal Index scores across groups using an analysis of covariance (ANCOVA) model controlling for maternal verbal IQ. The primary comparison is between subjects in the PWWE and PWWoE groups, with multiple additional tests comparing subgroups. For the purpose of power calculations, verbal index score was modeled as a standard IQ that follows a normal distribution with mean 100 and standard deviation 15. The correlation between maternal IQ and child verbal index score was assumed to be 0.40. The assumed dropout rate for this endpoint is 20%. There are N = 280 (350 enrolled minus 20% dropout) evaluable subjects in the PWWE group, and N = 80 (100 enrolled minus 20% dropout) evaluable subjects in the PWWoE group. The number of evaluable subjects in each PWWE monotherapy subgroup is N = 56 (70 enrolled minus 20% dropout). For the subgroup of PWWE

© 2026 Li Y et al. *JAMA Network Open*.

subjects not taking an AED, the assumed evaluable sample size is N = 28 (35 enrolled minus 20% dropout).

Power for each comparison was computed via simulation, with each mother (IQ) and child (verbal index score) data pair randomly sampled from a bivariate normal distribution. For cases where the mother used AED's during pregnancy, the data was simulated from a $N\left(\begin{bmatrix}100\\92.5\end{bmatrix}, \begin{bmatrix}15^2 & 0.4 \times 15^2\\0.4 \times 15^2 & 15^2\end{bmatrix}\right)$ distribution. For cases where the mother did not use AED's during pregnancy, the data was simulated from a $N\left(\begin{bmatrix}100\\100\end{bmatrix}, \begin{bmatrix}15^2 & 0.4 \times 15^2\\0.4 \times 15^2 & 15^2\end{bmatrix}\right)$ distribution.

Likelihood ratio tests of the linear model were performed to obtain p-values for the primary analysis. These tests compared a full model with both maternal IQ and study group as predictors to a reduced model with only maternal IQ as a predictor. For the primary analysis comparing subjects in the PWWE group to subjects in the PWWoE group, the power was computed as 96.0%.

Power was also estimated for nine secondary analyses, which were adjusted for multiple comparisons using the Holm procedure. These analyses used simulated data generated under the same assumptions as for the primary analyses, with likelihood ratio tests again used to compute p-values. The nine tests compared: 1) PWWE AED exposed to PWWoE, 2) PWWE AED monotherapy to PWWoE, 3) PWWE AED polytherapy to PWWoE 4) PWWE AED exposed to PWWE No AED, 5) PWWE CBZ monotherapy to PWWoE 6) PWWE LEV monotherapy to PWWoE 7) PWWE LTG monotherapy to PWWoE 8) PWWE other monotherapy to PWWoE, and 9) PWWE No AED to PWWoE.

Power for these secondary analyses are shown in the below table.

© 2026 Li Y et al. *JAMA Network Open*.

Comparison	Power
**Primary**	
Test of H0: All groups the same	96.0%
**Secondary** *(Holm adj. for multiple comparisons)*	
AED exposed compared to PWWoE	93.6%
AED mono. compared to PWWoE	92.9%
AED poly. compared to PWWoE	52.1%
AED exposed compared to PWWE No AED	61.0%
CBZ mono. compared to PWWoE	74.0%
LEV mono. compared to PWWoE	73.5%
LTG mono. compared to PWWoE	72.4%
Other mono. compared to PWWoE	72.5%
PWWE no AED compared to PWWoE	1.8%

Verbal – Non-Verbal Difference Scores: The difference between verbal and non-verbal scores will be computed for each subject. The primary comparison is between difference measures in all AED-exposed children (N=252, 315 enrolled minus 20% dropout) and healthy children (N=80, 100 enrolled minus 20% dropout). Here we compute the minimum difference (in terms of SD) that would be required to achieve 80% power for the comparison. We assume that difference scores in both groups have a normal distribution, with p-values generated with t-tests (a=0.05, two-sided). A background difference of 0.37 SD would be required to achieve 80% power for the primary comparison.

Power was also computed for the comparison of difference scores in a single AED monotherapy subgroup (N=56, 70 enrolled minus 20% dropout) and the PWWoE group. In this case, a background difference of 0.50 SD would be required to achieve 80% power for the comparison.

Handedness: The primary comparison is the rate of non-dextrals in the PWWE AED-exposed group (N=252, 315 enrolled minus 20% dropout) and the PWWoE group (N=80, 100 enrolled minus 20% dropout). It is assumed that 24.0% of children in the PWWE AED-exposed group are non-dextral, and that 10% of the PWWoE children are non-dextral. The power for this comparison was computed via simulation and Fishers Exact Test; the power was estimated to be 80%.

Power was also computed for the comparison of a single PWWE monotherapy subgroup (N=56, 70 enrolled minus 20% dropout) to the PWWoE group. It was assumed that the non-dextral rate in the single monotherapy group was 29.2%. The power for this comparison was estimated to be 80%.

Neonatal: The primary comparison is the rate of SGA in PWWE subjects vs. PWWoE. It is assumed that 10% of subjects will drop out by the birth visit, giving N = 315 evaluable subjects

© 2026 Li Y et al. *JAMA Network Open*.

in the PWWE group and N = 90 evaluable subjects in the PWWoE group. It was assumed that 10% of subjects in the PWWoE group would have babies that were SGA, and power calculations were performed to determine the SGA rate in PWWE subjects that would be needed to provide at least 80% power for the comparison. Power calculations were performed using Fishers Exact Test (a=0.05, two-sided). A background SGA rate of at least 23.3% in the PWWE group would provide >80% power for the comparison.

Calculations were also performed for the comparison of a single PWWE monotherapy group (N=63, 70 enrolled with 10% dropout) to the union of all other PWWE AED exposed groups (N=220, 245 enrolled with 10% dropout). With the SGA rate in the PWWoE group assumed to be 10%, an SGA rate of at least 25.6% would provide >80% power for the comparison.

8.3 Data Monitoring

Retention and other milestones will be specified by NINDS. An Observational Data Monitoring Committee (ODMB) may be appointed by the MONEAD PIs and approved by the NINDS.

8.4 Statistical Analyses

Neonatal Outcomes: Overall comparisons of neonatal outcomes such as SGA in PWWE compared to PWWoE subjects will be performed via Fishers Exact Test, or via logistic regression models predicting SGA status while controlling for other covariates of interest.

Depression: Rates of depression in the PWWE and PWWoE groups will be compared both during pregnancy and during the post-partum period. Additional comparisons will be made to NPWWE subjects using similar time periods. Comparisons between groups will be made via Fishers Exact Test, or via logistic regression models that control for covariates of interest.

Cognitive Outcomes: Analyses of cognitive outcomes such as verbal intellectual ability at 6 years will be analyzed via multiple regression models, with Child Verbal Intellectual Ability as the outcome controlling for other explanatory variables. Covariates suspected of being highly predictive of child IQ (e.g. maternal verbal ability, AED category, fetal AED exposure, breastfeeding status) will be included in the model *a priori* and subsequent covariates will be added based on pre-specified inclusion criteria. Additional covariates include those found significant in other analyses (e.g. SGA status, convulsant seizures during pregnancy, major depressive episodes). Results will be reported as an effect size along with 95% confidence interval. Secondary analyses of longitudinal cognitive outcomes will be performed via linear mixed models that control for some covariates *a priori* in a similar manner. Cerebral Lateralization will be assessed as verbal minus non-verbal difference scores and proportion of dextral children in PWWE vs. HPW children via a multiple regression model with the paired difference in scores as the outcome and adjusting for similar covariates.

Behavioral Outcomes: Analyses of ABAS-3 and BASC-2 responses will be conducted similarly to those for cognitive outcomes, fitting multiple regression models with similar pre-specified covariates.

Breastfeeding: Analyses of breastfeeding will be conducted via inclusion of binary breastfeeding status into multiple regression models outlined in the cognitive outcomes section. Subanalyses will assess if there are differential effects on cognition due to frequency of

© 2026 Li Y et al. *JAMA Network Open*.

breastfeeding, type of feeding (ratio of breast milk to formula, ratio of breastfed to bottle fed), accounting for missing data inherent in collection via electronic diary entry.

Sensitivity Analyses – Effects of Confounders: To analyze the potential impact of any imbalance between study groups with respect to baseline covariates, sensitivity analyses will be conducted to analyses of primary endpoints. Propensity scores will be computed for each subject, and subgroups will be computed by matching scores for subjects in each study group. Analyses of these subgroups will indicate whether results are sensitive to baseline covariate imbalance. Sensitivity analyses will also be conducted for analyses including twin births, comparing modeling results with twins included to those where only a single (first enrolled) twin is included.

Sensitivity Analyses – Effects of Missing Data: It is expected that substantial missing data will occur in MONEAD due to the longitudinal nature of the study, and any analyses need to appropriately account for this missing-ness. For analyses of outcomes with substantial missing data, multiple imputation methods assuming the data are missing at random will be performed. Specifically, a Markov Chain Monte Carlo (MCMC) method will be employed; this method creates multiple imputations by drawing simulations from a Bayesian predictive distribution for normal data and allows for non-monotone missing data patterns that are likely to be present. Additional analyses will assess the sensitivity of results to the MCMC imputation utilized. These include complete case analyses, where models are fit to only observed data (assuming the data are Missing Completely at Random); last observation carried forward, where (for example) IQ data missing at subsequent visits is imputed to be the IQ from the most recent visit; pattern mixture model and selection model that accounts for data that are Missing not at Random.

9 DATA COLLECTION, SITE MONITORING, AND ADVERSE EXPERIENCE REPORTING

9.1 Records to Be Kept

Each participating site will maintain appropriate medical and research records for this trial, in compliance with ICH-GCP, regulatory and institutional requirements for the protection of confidentiality of subjects. As part of participating in the NINDS-sponsored study, each site will permit authorized representatives of the Data and Statistical Center, NINDS/NIH, MONEAD Central Administrative Center, and local and national regulatory agencies to examine (and when required by applicable law, to copy) clinical records for the purposes of quality assurance reviews, audits and evaluation of the study safety and progress. Source data are all information, original records of clinical findings, observations, or other activities in a clinical trial necessary for the reconstruction and evaluation of the trial. Examples of these original documents and data records include, but are not limited to, medical records, laboratory reports, pharmacy records, neuropsychological test record forms, questionnaires, and other research records maintained for the clinical trial.

The site PI will maintain a list of appropriately qualified persons to whom trial duties have been delegated. The site PI is responsible for ensuring that staff maintains a comprehensive and centralized filing system of all study-related (essential) documentation, suitable for inspection at any time by representatives from The Emmes Corporation, the Central Administrative Center,

© 2026 Li Y et al. *JAMA Network Open*.

and/or applicable regulatory authorities. Elements include but are not limited to:

- Subject files containing completed informed consent forms and supporting copies of source documentation.
- Study files containing the protocol with all amendments and copies of all correspondence with the IRB.

In addition, all original source documentation must be maintained and readily available. Records and documents pertaining to the conduct of this study, including CRFs, source documents, consent forms, laboratory test results, and medication inventory records, must be retained by the investigator for at least 3 years after the date of the final Financial Status Report (FSR).

The investigator is responsible for ensuring the accuracy, completeness, legibility, and timeliness of the data reported.

Data collection forms will be derived from the eCRFs and provided by the DSC to the sites to record and maintain data for each subject enrolled in the study. All source documents should be completed in a neat, legible manner to ensure accurate interpretation of data. Permanent ink is required to ensure clarity of reproduced copies. When making a change or correction, the original entry should be crossed out with a single line, and the change should be initialed and dated. Do not erase, overwrite, or use correction fluid or tape on the original.

Data reported in the eCRF should be consistent with the data collection form/source documents or the discrepancies should be documented.

The sponsor and/or its designee will provide guidance to investigators on making corrections to the data collection forms and eCRFs.

9.2 Role of Data Management

Clinical research data will be collected and recorded by the study sites in a timely fashion in a secure electronic web-based clinical data management system (CDMS) provided by The Emmes Corporation.

All data collection forms and laboratory reports must be reviewed by the clinical team and data entry staff, who will ensure that they are accurate and complete. Adverse events must be graded, assessed for severity and causality, and reviewed by the site principal investigator or designee. Data collection is the responsibility of the clinical trial staff at the site under the supervision of the site principal investigator. During the study, the investigator must maintain complete and accurate documentation for the study.

The Emmes Corporation will serve as the Data and Statistical Center (DSC) for this study, and will be responsible for data management, quality review, analysis, and reporting of the study data.

Clinical data (including SAEs, concomitant medications) will be entered into a 21 CFR Part 11-compliant internet data entry system provided by The Emmes Corporation. The data system

© 2026 Li Y et al. *JAMA Network Open*.

includes password protection and internal quality checks, such as automatic range checks, to identify data that appear inconsistent, incomplete, or inaccurate. Clinical data will be entered directly from the data collection forms/source documents.

9.3 Quality Assurance

The study data integrity and compliance with the protocol will be assured by the monitoring of the study documentation and study conduct at the sites by The Emmes Corporation. Neuropsychological test accuracy and Q/A review will be performed continuously by the Project Manager. Routine data monitoring and protocol compliance will be performed by the site investigators and study coordinator on an ongoing basis. The study clinical monitoring plan and the data quality monitoring plan will be developed and followed by The Emmes Corporation.

The Data and Statistical Center will implement quality control procedures beginning with the data entry system and generate data quality control checks that will be run on the database. Any missing data or data anomalies will be communicated to the site(s) for prompt clarification and resolution.

9.4 Adverse Experience Reporting

All serious adverse events (as defined by Section 7) must be entered into the AdvantageEDC system within 24 hours of discovery. Any SAE entered into the study database will generate automatic email notification to the Medical Monitor.

The investigator must submit additional information as it becomes available and/or upon request of the Medical Monitor.

9.5 Reporting to Site IRBs

Each site PI is responsible for reporting adverse events to the site IRB in accordance with their IRB's requirements for expedited reporting and continuing review reporting.

10 HUMAN SUBJECTS

10.1 Institutional Review Board (IRB) Review and Informed Consent

This protocol and the informed consent document and any subsequent modifications will be reviewed and approved by the IRB or ethics committee responsible for oversight of the study. A signed consent form will be obtained from the subject. For subjects who cannot consent for themselves, such as those below the legal age, a parent, legal guardian, or person with power of attorney, must sign the consent form; additionally, the subject's assent must also be obtained if he or she is able to understand the nature, significance, and risks associated with the study. The consent form will describe the purpose of the study, the procedures to be followed, and the risks and benefits of participation. A copy of the consent form will be given to the subject, parent, or legal guardian, and this fact will be documented in the subject's record.

© 2026 Li Y et al. *JAMA Network Open*.

10.2 Subject Confidentiality

All evaluation forms and other records that leave the site will be identified only by the Study Identification Number (SID) to maintain subject confidentiality. Remaining Obstetric and Neonatal records that are submitted to the OB Core will contain dates (child's D.O.B., date of obstetric and birth related events) and locations (delivering hospital, study hospital, city, state). Video and/or audio recordings used for staff training and review that are submitted to the Psychiatry Core and Neuropsychology Core will contain a biomedical identifier (voice). All records and recordings will be kept in locked file cabinets. Records will be kept for 5 years after the study's conclusion. Video and/or audio recordings will be destroyed immediately after review and documentation. All computer entry and networking programs will be done using SIDs only. Clinical information will not be released without written permission of the subject, except as necessary for monitoring by IRB, the FDA, the NINDS, the OHRP, the sponsor, or the sponsor's designee

The National Institutes of Health has given a Certificate of Confidentiality for this study. This will be used to block a legal request to give out information about a patient's participation in a study.

10.3 Study Modification/Discontinuation

The study may be modified or discontinued at any time by the IRB, the NINDS, the sponsor, the OHRP, the FDA, or other government agencies as part of their duties to ensure that research subjects are protected.

11 PUBLICATION OF RESEARCH FINDINGS

All presentations, abstracts, or manuscripts directly resulting from or referencing the project will be presented to the Publications Committee to obtain permission to publish and editorial review prior to presentation to the NINDS for review prior to submission. Authors must obtain permission from both the MONEAD Publications Committee AND NINDS before submitting the presentation/publication to the journal or professional organization.

The Core will be directed by David Loring, Ph.D. and consist of the PIs (Kimford Meador, Page Pennell, Abigail Matthews) and Project Managers (Gene Moore, Dominic Ippolito). The members of the Executive Committee will also serve on the Publications Committee when the publication is in regards to their specialty. The Director will determine which Executive Committee members to include in the reviews.

Role and Responsibilities

- Develop and coordinate publication activities of the MONEAD Study.
- Advise the MONEAD Executive Committee on publications projects.
- Serve as liaison to the authors and the MONEAD Executive Committee.
- Stimulate and encourage projects, which may lead to publication of MONEAD data.
- Assist the authors in finding appropriate avenues for publications.
- Assess any potential conflicts of interest and advise the MONEAD Executive Committee.

© 2026 Li Y et al. *JAMA Network Open*.

- Assure that there is not substantial overlap or conflict in proposed publications.
- Adjudicate authorship inclusion and order.
    - Where journal policy permits, all investigators who contribute subjects to the trial will be acknowledged.
- Authorship inclusion and order will be based upon intellectual input, study design, data collection, data analyses and interpretation, and writing and review of the publication.
- Approve all publication projects that include MONEAD data.
- Assure that all publications include appropriate credit of the funding source.
- Assure that manuscripts are completed and submitted in a timely fashion.
- Review, edit appropriately, and approve all abstracts, research articles, reviews, presentations, and web-based publications of MONEAD data prior to submission.
- There will be no publication of single-center results before multicenter results are published.

Proposed publications and presentations can be presented by any member of the MONEAD group. The Publication Committee will determine if the publication and presentation prospects are valid. The Publication Committee will offer editorial review, statistical advice, and assist in the collection of disclosure and copyright documents as required by the journal editors.

A master list of these publications and presentations will be maintained by Gene Moore and made available for progress reports and investigator biosketch maintenance.

© 2026 Li Y et al. *JAMA Network Open*.

12 REFERENCES

1. Wyszynski DF, Nambisan M, Surve T et. al. Antiepileptic Drug Pregnancy Registry. Increased rate of major malformations in offspring exposed to valproate during pregnancy. Neurology 2005;64:961–965.
2. Hunt RW, Tzioumi D, Collins E et. al. Adverse neurodevelopmental outcome of infants exposed to opiate in-utero. Early Hum Dev. 2008;84(1):29-35. Epub 2007 Aug 28.
3. Hernandez-Diaz S, Mittendorf R, Holmes LB. Comparative Safety of Topiramate During Pregnancy. Birth Defects Research (Part A). 2010;88:408
4. Meador KJ, Baker GA, Browning N et al. Cognitive function at 3 years of age after fetal exposure to antiepileptic drugs. N Engl J Med. 2009;360:1597-1605.
5. Harden CL, Hopp J, Ting TY et al. Practice Parameter update: Management issues for women with epilepsy--focus on pregnancy (an evidence-based review): Obstetrical complications and change in seizure frequency. Report of the Quality Standards Subcommittee and Therapeutics and Technology Assessment Subcommittee of the American Academy of Neurology and American Epilepsy Society. Neurology. 2009;73:126-132.
6. Harden CL, Meador KJ, Pennell PB et al. Practice Parameter update: Management issues for women with epilepsy--focus on pregnancy (an evidence-based review): Teratogenesis and perinatal outcomes. Report of the Quality Standards Subcommittee and Therapeutics and Technology Subcommittee of the American Academy of Neurology and American Epilepsy Society. Neurology. 2009;73:133-141.
7. Harden CL, Pennell PB, Koppel BS et al. Practice Parameter update: Management issues for women with epilepsy--focus on pregnancy (an evidence-based review): Vitamin K, folic acid, blood levels, and breastfeeding. Report of the Quality Standards Subcommittee and Therapeutics and Technology Assessment Subcommittee of the American Academy of Neurology and American Epilepsy Society. Neurology. 2009;73:142-149.
8. Meador KJ, Penovich P, Baker GA et al. Antiepileptic drug use in women of childbearing age. Epilepsy Behav. 2009;15:339-343.
9. Sabers A. Influences on seizure activity in pregnant women with epilepsy. Epilepsy Behav. 2009;15:230-234.
10. Seizure control and treatment in pregnancy: observations from the EURAP epilepsy pregnancy registry. Neurology. 2006;66:354-360.
11. Sabers A, Petrenaite V. Seizure frequency in pregnant women treated with lamotrigine monotherapy. Epilepsia. 2009;50:2163-2166.
12. Pennell PB, Hovinga CA. Antiepileptic drug therapy in pregnancy I: gestation-induced effects on AED pharmacokinetics. Int Rev Neurobiol. 2008;83:227-240.
13. Pennell PB, Peng L, Newport DJ et al. Lamotrigine in pregnancy: clearance, therapeutic drug monitoring, and seizure frequency. Neurology. 2008;70:2130-2136.
14. Petrenaite V, Sabers A, Hansen-Schwartz J. Seizure deterioration in women treated with oxcarbazepine during pregnancy. Epilepsy Res. 2009;84:245-249.

© 2026 Li Y et al. *JAMA Network Open*.

12. REFERENCES *(Continued)*

15. Petrenaite V, Sabers A, Hansen-Schwartz J. Individual changes in lamotrigine plasma concentrations during pregnancy. Epilepsy Res. 2005;65:185-188.
16. Vajda F, Solinas C, Graham J et al. The case for lamotrigine monitoring in pregnancy. J Clin Neurosci. 2006;13:103-104.
17. Mawer G, Briggs M, Baker GA et al. Pregnancy with epilepsy: obstetric and neonatal outcome of a controlled study. Seizure. 2010;19:112-119.
18. Adab N, Kini U, Vinten J et al. The longer term outcome of children born to mothers with epilepsy. J Neurol Neurosurg Psychiatry. 2004;75:1575-1583.
19. Kelly VM, Nelson LM, Chakravarty EF. Obstetric outcomes in women with multiple sclerosis and epilepsy. Neurology. 2009;73:1831-1836.
20. Veiby G, Daltveit AK, Engelsen BA, Gilhus NE. Pregnancy, delivery, and outcome for the child in maternal epilepsy. Epilepsia. 2009;50:2130-2139.
21. Alder J, Fink N, Bitzer J et al. Depression and anxiety during pregnancy: a risk factor for obstetric, fetal and neonatal outcome? A critical review of the literature. J Matern Fetal Neonatal Med. 2007;20:189-209.
22. Levey L, Ragan K, Hower-Hartley A et al. Psychiatric disorders in pregnancy. Neurol Clin. 2004;22:863-893.
23. Newport DJ, Levey LC, Pennell PB et al. Suicidal ideation in pregnancy: assessment and clinical implications. Arch Womens Ment Health. 2007;10:181-187.
24. Ross LE, McLean LM. Anxiety disorders during pregnancy and the postpartum period: A systematic review. J Clin Psychiatry 2006;67:1285-1298.
25. Huizink AC, Mulder EJ, Robles de Medina PG et al. Is pregnancy anxiety a distinctive syndrome? Early Hum Dev. 2004;79:81-91.
26. Huizink AC, Robles de Medina PG, Mulder EJ et al. Stress during pregnancy is associated with developmental outcome in infancy. J Child Psychol Psychiatry. 2003;44:810-818.
27. van Batenburg-Eddes T, de Groot L, Huizink AC et al. Maternal symptoms of anxiety during pregnancy affect infant neuromotor development: the generation R study. Dev Neuropsychol. 2009;34:476-493.
28. Van den Bergh BR, Marcoen A. High antenatal maternal anxiety is related to ADHD symptoms, externalizing problems, and anxiety in 8- and 9-year-olds. Child Dev. 2004;75:1085-1097.
29. Van den Bergh BR, Mennes M, Oosterlaan J et al. High antenatal maternal anxiety is related to impulsivity during performance on cognitive tasks in 14- and 15-year-olds. Neurosci Biobehav Rev. 2005;29:259-269.
30. Deave T, Heron J, Evans J, Emond A. The impact of maternal depression in pregnancy on early child development. BJOG. 2008;115:1043-1051.
31. Hay DF, Pawlby S, Waters CS, Sharp D. Antepartum and postpartum exposure to maternal depression: different effects on different adolescent outcomes. J Child Psychol Psychiatry. 2008;49:1079-1088.

© 2026 Li Y et al. *JAMA Network Open*.

12. REFERENCES *(Continued)*

32. O'Connor TG, Heron J, Golding J et al. Maternal antenatal anxiety and children's behavioural/emotional problems at 4 years. Report from the Avon Longitudinal Study of Parents and Children. Br J Psychiatry. 2002;180:502-508.

33. O'Connor TG, Heron J, Golding J, Glover V. Maternal antenatal anxiety and behavioural/emotional problems in children: a test of a programming hypothesis. J Child Psychol Psychiatry. 2003;44:1025-1036.

34. Carter AS, Garrity-Rokous FE, Chazan-Cohen R et al. Maternal depression and comorbidity: predicting early parenting, attachment security, and toddler social-emotional problems and competencies. J Am Acad Child Adolesc Psychiatry. 2001;40:18-26.

35. Horwitz SM, Briggs-Gowan MJ, Storfer-Isser A, Carter AS. Prevalence, correlates, and persistence of maternal depression. J Womens Health (Larchmt). 2007;16:678-691.

36. Luoma I, Kaukonen P, Mantymaa M et al. A longitudinal study of maternal depressive symptoms, negative expectations and perceptions of child problems. Child Psychiatry Hum Dev. 2004;35:37-53.

37. Luoma I, Tamminen T, Kaukonen P et al. Longitudinal study of maternal depressive symptoms and child well-being. J Am Acad Child Adolesc Psychiatry. 2001;40:1367-1374.

38. Grace SL, Evindar A, Stewart DE. The effect of postpartum depression on child cognitive development and behavior: a review and critical analysis of the literature. Arch Womens Ment Health. 2003;6:263-274.

39. Ettinger A, Reed M, Cramer J, Epilepsy Impact Project Group. Depression and comorbidity in community-based patients with epilepsy or asthma. Neurology. 2004;63:1008-1014.

40. Turner K, Piazzini A, Franza A et al. Postpartum depression in women with epilepsy versus women without epilepsy. Epilepsy Behav. 2006;9:293-297.

41. Galanti M, Newport DJ, Pennell PB et al. Postpartum depression in women with epilepsy: influence of antiepileptic drugs in a prospective study. Epilepsy Behav. 2009;16:426-430.

42. Turner K, Piazzini A, Franza A et al. Epilepsy and postpartum depression. Epilepsia. 2009;50 Suppl 1:24-27.

43. Meador KJ, Baker G, Cohen MJ et al. Cognitive/behavioral teratogenetic effects of antiepileptic drugs. Epilepsy Behav. 2007;11:292-302.

44. Lundgren EM, Tuvemo T. Effects of being born small for gestational age on long-term intellectual performance. Best Pract Res Clin Endocrinol Metab 2008;22(3):477-88.

45. De la Calzada DG, García LO, Remírez JM, Lázaro A, Cajal MD. Study of early detection of cardiovascular risk factors in children born small (SGA) and review of literature. Pediatr Endocrinol Rev 2009;6 (Suppl 3):343-9.

46. Chen YH, Chiou HY, Lin HC, Lin HL. Affect of seizures during gestation on pregnancy outcomes in women with epilepsy. Arch Neurol. 2009;66:979-984.

47. Meador KJ, Pennell PB, Harden CL et al. Pregnancy registries in epilepsy: a consensus statement on health outcomes. Neurology. 2008;71:1109-1117.

© 2026 Li Y et al. *JAMA Network Open*.

12. REFERENCES *(Continued)*

48. Ip S, Chung M, Raman G et al. A summary of the Agency for Healthcare Research and Quality's evidence report on breastfeeding in developed countries. Breastfeed Med. 2009;4 Suppl 1:S17-30.

49. Eckholm E. ’07 US births break baby boom record. 2009. http://www.nytimes.com/2009/03/19/health/19birth.html?_r=1&partner=rss&emc=rss.

50. Chambers J, Parrish T, Harr J. Special education expenditure project: what are we spending on special education services in the United States, 1999-2000? . Washington, DC: American Institutes for Research, 2002

51. Adab N, Jacoby A, Smith D, Chadwick D. Additional educational needs in children born to mothers with epilepsy. J Neurol Neurosurg Psychiatry. 2001;70:15-21.

52. Grosse SD, Matte TD, Schwartz J, Jackson RJ. Economic gains resulting from the reduction in children's exposure to lead in the United States. Environ Health Perspect. 2002;110:563-569.

53. Gaily E, Kantola-Sorsa E, Hiilesmaa V et al. Normal intelligence in children with prenatal exposure to carbamazepine. Neurology. 2004;62:28-32.

54. Bromley RL, Mawer G, Love J, Kelly J, Purdy L, McEwan L, Briggs M, Smith JC, Sin X, Baker GA. Early cognitive development in children born to women with epilepsy: A prospective report. Epilepsia 2010;51:2058-65.

55. Meador KJ, Baker GA, Browning N, Clayton-Smith J, Combs-Cantrell DT, Cohen M, Kalayjian LA, Kanner A, Liporace JD, Pennell PB, Privitera M, and Loring DW for the NEAD Study Group. Effects of breastfeeding in children of women on antiepileptic drugs. Neurology 2010 (in press).

56. Thomas SV, Ajaykumar B, Sindhu K, Nair MK, George B, Sarma PS. Motor and mental development of infants exposed to antiepileptic drugs *in utero*. Epilepsy Behav. 2008;13(1):229-36.

57. Dreyer NA, Garner S. Registries for robust evidence. JAMA. 2009;302:790-791.

© 2026 Li Y et al. *JAMA Network Open*.

APPENDICES

© 2026 Li Y et al. *JAMA Network Open*.

APPENDIX A:
PSYCHIATRIC SYMPTOM ONSET AND/OR WORSENING/DECOMPENSATION OVER COURSE OF STUDY PARTICIPATION

Symptom Rating Scale	Clinical Response by the Investigative Team	Research activities
BDI-II 210	If yes, the participant will be asked the first 2 questions of the major depression mood module about depression and loss of pleasure by any member of the study staff.<br><br>If the participant answers "yes" to either question, she should be referred to the site Certified SCID assessor for full SCID current major depression mood module completion.<br><br>If participant does not have an established clinician, provide referrals.	1. Document intervention and provide details for the Medical Monitor.<br><br>2. Confirm that subject is willing to continue in the study.

© 2026 Li Y et al. *JAMA Network Open*.

APPENDIX A *(continued)*

Endorsement of Suicidal Ideation

Symptom Rating Scale	Clinical Response by the Investigative Team	Research activities
BDI-II Item # 9 2 1<br>and/or	1. Assess acuity of the +SI. e.g. active plan, history of prior attempts, family history of suicide.<br>High acuity is defined as BDI-II > 2. If acuity is high, the patient will be immediately referred to the geographic emergency services for acute evaluation. This will require arranging safe transport to that facility.<br>Low acuity is defined as BDI-II =1.. If <u>acuity is low</u>, and participant is able to contract for no self-harm and has responsible support system available – then an "urgent" evaluation and/or intervention is not warranted and steps 2-5 below are not required.<br>2. Is patient in active treatment for depression/anxiety?<br><u>If yes</u>, can they be seen within 72 hours, and if not then refer to emergency services.<br><u>If no</u>, staff will arrange for an urgent appointment with one of the mental health providers. If such is not possible, then subject will be referred to the regional emergency services<br>3. Provide contact information for resources in the event of further symptom worsening and/or the development of +SI. This will include the suicide hotline.<br>4. Follow up contact in 72 hours to see if participant has made contact with care provider.<br><u>If yes</u>, complete documentation of any intervention.<br><u>If no</u>, encourage contact, and query by phone if suicidal ideation has resolved.<br>5. If SI continues and no contact with care provider has been made, then refer to local emergency services.	1. Document intervention and provide details for the Medical Monitor.<br>2. Confirm that subject is willing to continue in the study.

© 2026 Li Y et al. *JAMA Network Open*.

APPENDIX B: CLINICAL AND LABORATORY EVALUATIONS SCHEDULE

**Visit**	**Study Visits 8A-8C**	**Study Visit 9**	**Study Visit 9A-C**	**Study Visit 10**	**Study Visit 10A-E**	**Study Visit 11**	**Study Visit 11A-E**	**Study Visit 12**
**Child age**	**15-21M**	**2 Y**	**27-33m**	**3Y**	**39-51m**	**4.5 Y**	**57-69m**	**6 Y**
**Maternal Clinical Evaluations**								
Informed Consent *								
Behavioral History	X	X						
SES		X		X		X		X
Medical History	X	X	X	X	X	X	X	X
Buccal Swab Collection						X^{V}		X^{V}
**Maternal Administered Assessments and Questionnaires**								
SCID mood module **		X**		X**		X**		X**
Edinburgh Handedness Inventory		X		X		X		X
Mood Scales(BDI/BAI/PSS)		X		X		X		X
McMaster Family Assessment Device		X		X		X		X
Parent Stress Index (PSI)		X		X		X		X
Social Responsiveness Scale						X		X
Behavior Assessment System for Children (BASCP-2)		X		X		X		X
Behavior Assessment System for Children Teacher (BASCT-2)								X
Behavior Rating Inventory of Executive Function (BRIEF) (P/V2)				X		X		X***
Adaptive Behavior Assessment System (ABAS3)				X		X		X
COVID Impact Questionnaire						X^{IV}		X^{IV}
**Child Clinical Evaluations**								
Child Physical & Neurological Exam		X		X		X		X
Head Circumference		X		X		X		X
Child Health Update	X	X	X	X	X	X	X	X
Buccal Swab Collection						X^{V}		X^{V}
**Child Administered Assessments and Questionnaires**								
BSID 3		X		X				
Differential Abilities Scale (DAS 2)				X				X
Peabody Picture Vocabulary Test (PPVT 4)				X				X
Beery Visual Motor Integraton (VMI 6)				X				X
Preschool Language Scale (PLS 5)				X				
Torrance Creativity						X		
NEPSY								X
Expressive One-Word Vocabulary Test (EOWVT)								X
WISC Coding								X
Children's Memory Scale (CMS)								X
Child Modified Edinburgh Handedness Inventory		X		X				X
Grooved Pegboard								X
WRAT 5								X
MCHAT Autism		X						
GARS-2 Autism				X				

* subjects will be re-consented at the earliest visit following institutional approval ** See appendix A for the times to administer the SCID Mood module ***BRIEF2 V12 only
IV Collected once at either V11 or V12 V Buccal Swab collected once at either V11 or V12

© 2026 Li Y et al. *JAMA Network Open*.

APPENDIX C:

CHILD NEUROPSYCHOLOGICAL REFERRAL REQUIREMENTS

Two Year Old Assessment

Early Intervention Services Referral Requirements

**BSID-III**	**Clinical Response by the Investigative Team**
::70 (standard score) on *any one* of the following composites on the BSID-III<br>--OR--<br>::77 (standard score) on *two or more* of the following composites:<br>• Cognitive Composite<br>• Language Composite<br>• Motor Composite<br>• Social Emotional Composite<br>• General Adaptive Composite	If the criteria for referral are met:<br><br>1. Refer the child subject to the Early Intervention Program in your site's area for further assessment and follow-up.<br><br>2. Remain in contact with the parent to determine if the child ultimately qualified for Early Intervention Services.<br><br>a. If the child qualified, determine what type(s) of service(s) were provided (and for how long).<br><br>*Information pertaining to the referral must be entered on the Developmental Delay Referral form.*<br><br>*Information pertaining to the developmental delay diagnosis(es) and services received must be entered on the Developmental Delays form.*

Autism Spectrum Disorder Screening Referral Requirements:

**M-CHAT**	**Clinical Response by the Investigative Team**
MCHAT Total score 23<br>--OR--<br>MCHAT Critical score 22	If the criteria for referral are met:<br><br>1. Refer the child subject for a more formal diagnostic evaluation by a psychologist or psychiatrist trained to evaluate Autism Spectrum Disorder in young children at the parent's expense.<br><br>2. Remain in contact with the parent to obtain follow-up on the results of the evaluation.<br><br>*Information pertaining to the referral and the child's evaluation results must be entered on the Autism Referral form, which will be entered directly onto the MCHAT eCRF in the data system.*<br><br>*Information pertaining to the developmental delay diagnosis(es) and services received must be entered on the Developmental Delays form.*

© 2026 Li Y et al. *JAMA Network Open*.

Three Year Old Assessment

Preschool Special Education Services Referral Requirements

**Referral Criteria**	**Clinical Response by the Investigative Team**
One of the following criteria is met:<br>• DAS-2 GCA score ::70<br>• PLS Auditory Comprehension score ::70<br>• PLS Expressive Communication score ::70<br>• PPVT score ::70<br>• BSID-III Motor Composite score ::70<br>• ABAS-3 GAC score ::70<br>• BASC-2 Externalizing Problems Composite 270<br>• BASC-2 Internalizing Problems Composite 270<br>• BASC-2 Behavioral Symptoms Index 270<br><br>Two or more of the following criteria are met:<br>• DAS-2 GCA score ::77<br>• PLS Auditory Comprehension score ::77<br>• PLS Expressive Communication score ::77<br>• PPVT score ::77<br>• BSID-III Motor Composite score ::77<br>• ABAS-3 GAC score ::77<br>• BASC-2 Externalizing Problems Composite 265<br>• BASC-2 Internalizing Problems Composite 265<br>• BASC-2 Behavioral Symptoms Index 265	If the criteria for referral are met:<br><br>1. Refer the child subject to the Preschool Special Education Program in your site's area for further assessment and follow-up.<br><br>2. Remain in contact with the parent to determine if the child ultimately qualified for Preschool Special Education Services.<br><br>a. If the child qualified, determine what type(s) of service(s) were provided (and for how long).<br><br>*Information pertaining to the referral must be entered on the Developmental Delay Referral form.*<br><br>*Information pertaining to the developmental delay diagnosis(es) and services received must be entered on the Developmental Delays form.*

Autism Spectrum Disorder Screening Referral Requirements:

**GARS**	**Clinical Response by the Investigative Team**
GARS Autism Index 285	If the criteria for referral are met:<br><br>1. Refer the child subject for a more formal diagnostic evaluation by a psychologist or psychiatrist trained to evaluate Autism Spectrum Disorder in young children at the parent's expense.<br><br>2. Remain in contact with the parent to obtain follow-up on the results of the evaluation.<br><br>*Information pertaining to the referral and the child's evaluation results must be entered on the Autism Referral form, which will be entered directly onto the GARS eCRF in the data system.*<br><br>*Information pertaining to the developmental delay diagnosis(es) and services received must be entered on the Developmental Delays form.*

© 2026 Li Y et al. *JAMA Network Open*.

Four Year Six Month Old Assessment

Preschool Special Education Services Referral Requirements

**Referral Criteria**	**Clinical Response by the Investigative Team**
One of the following criteria is met:<br>• ABAS-3 GAC score ::70<br>• BASC-2 Externalizing Problems Composite 270<br>• BASC-2 Internalizing Problems Composite 270<br>• BASC-2 Behavioral Symptoms Index 270<br><br>Two or more of the following criteria are met:<br>• ABAS-3 GAC score ::77<br>• BASC-2 Externalizing Problems Composite 265<br>• BASC-2 Internalizing Problems Composite 265<br>• BASC-2 Behavioral Symptoms Index 265	If the criteria for referral are met:<br>1. Refer the child subject for a more formal diagnostic evaluation by a psychologist or psychiatrist trained to evaluate Autism Spectrum Disorder in young children at the parent's expense.<br><br>2. Remain in contact with the parent to obtain follow-up on the results of the evaluation.<br><br>Information pertaining to the referral and the child's evaluation results must be entered on the Autism Referral form, which will be entered directly onto the GARS eCRF in the data system.<br>Information pertaining to the developmental delay diagnosis(es) and services received must be entered on the Developmental Delays form.

Autism Spectrum Disorder Screening Referral Requirements

**Social Responsiveness Scale-2**	**Clinical Response by the Investigative Team**
SRS-2 Total Score < 70	If the criteria for referral are met:<br><br>1. Refer the child subject for a more formal diagnostic evaluation by a psychologist or psychiatrist trained to evaluate Autism Spectrum Disorder in young children at the parent's expense.<br><br>1. Remain in contact with the parent to obtain follow-up on the results of the evaluation.<br><br>*Information pertaining to the referral and the child's evaluation results must be entered on the Autism Referral form, which will be entered directly onto the GARS eCRF in the data system.*<br>*Information pertaining to the developmental delay diagnosis(es) and services received must be entered on the Developmental Delays form.*

© 2026 Li Y et al. *JAMA Network Open*.

Six Year Old Assessment

Preschool Special Education Services Referral Requirements

**Referral Criteria**	**Clinical Response by the Investigative Team**
One of the following criteria is met:<br>• DAS-2 GCA score ::70<br>• EOWPVT score ≤ 70<br>• PPVT score ::70<br>• Phonological Processing (NEPSY-2) ≤70<br>• Comprehension of Instructions (NEPSY-2) ≤70<br>• Sentence Repetition (NEPSY-2) ≤70<br>• Word Definitions (DAS-2)≤70<br>• (DTVMI-6)≤70<br>• Coding/Symbol Search (WISC-5)≤70<br>• Grooved Pegboard dominant OR non-dominant hand standard score ≤70<br>• ABAS-3 GAC score ::70<br>• BASC-2 P or T Externalizing Problems Composite 270<br>• BASC-2 P or T Internalizing Problems Composite 270<br>• BASC-2 P or T Behavioral Symptoms Index 270<br><br>Two or more of the following criteria are met:<br>• DAS-2 GCA score ::77<br>• EOWPVT score ≤ 77<br>• PPVT score ::77<br>• Phonological Processing (NEPSY-2) ≤77<br>• Comprehension of Instructions (NEPSY-2) ≤77<br>• Sentence Repetition (NEPSY-2) ≤77<br>• Word Definitions (DAS-2)≤77<br>• (DTVMI-6)≤77<br>• Coding/Symbol Search (WISC-5)≤77<br>• Grooved Pegboard dominant hand standard score ≤77<br>• Grooved Pegboard non-dominant hand standard score ≤77<br>• ABAS-3 GAC score ::77<br>•<br>• BASC-2 P Externalizing Problems 265<br>• BASC-2 T Externalizing Problems 265<br>• BASC-2 P Internalizing Problems 265<br>• BASC-2 T Internalizing Problems 265<br>• BASC-2 P Behavioral Symptoms Index 265<br>• BASC-2 T Behavioral Symptoms Index 265<br>•	If the criteria for referral are met:<br><br>1. Refer the child subject to the School age Special Education Program in your site's area for further assessment and follow-up.<br><br>2. Remain in contact with the parent to determine if the child ultimately qualified for Preschool Special Education Services.<br><br>a. If the child qualified, determine what type(s) of service(s) were provided (and for how long).<br><br>*Information pertaining to the referral must be entered on the Developmental Delay Referral form*.<br><br>*Information pertaining to the developmental delay diagnosis(es) and services received must be entered on the Developmental Delays form.*

© 2026 Li Y et al. *JAMA Network Open*.

Autism Spectrum Disorder Screening Referral Requirements

**Social Responsiveness Scale-2**	**Clinical Response by the Investigative Team**
SRS-2 Total Score ≤ 70	If the criteria for referral are met:<br><br>3. Refer the child subject for a more formal diagnostic evaluation by a psychologist or psychiatrist trained to evaluate Autism Spectrum Disorder in young children at the parent's expense.<br><br>4. Remain in contact with the parent to obtain follow-up on the results of the evaluation.<br><br>*Information pertaining to the referral and the child's evaluation results must be entered on the Autism Referral form, which will be entered directly onto the GARS eCRF in the data system*.<br>*Information pertaining to the developmental delay diagnosis(es) and services received must be entered on the Developmental Delays form.*

Attention Deficit/Hyperactivity Disorder Screening Referral Requirements

**Behavior Assessment System for Children-2**	**Clinical Response by the Investigative Team**
BASC-2 P&T Attention Scale ≥ 65<br>BASC-2 P&T Attention & Hyperactivity Scales ≥ 65	If the criteria for referral are met:<br><br>5. Refer the child subject to his/her pediatrician for a more formal diagnostic evaluation of ADHD.<br><br>6. Remain in contact with the parent to obtain follow-up on the results of the evaluation.<br><br>*Information pertaining to the referral and the child's evaluation results must be entered on the Autism Referral form, which will be entered directly onto the GARS eCRF in the data system*.<br>*Information pertaining to the developmental delay diagnosis(es) and services received must be entered on the Developmental Delays form.*

© 2026 Li Y et al. *JAMA Network Open*.

Statistical Analysis Plan

Project Name: Breastfeeding Safety in Children of Women with Epilepsy Taking New-Generation Antiseizure Medication

Version: 1.0 **Date:** August 2025

1. OBJECTIVES

1.1 Primary Objectives

- Group Comparison: To compare neurodevelopmental outcomes at age 6 years between children of women with epilepsy (WWE) managed with newer-generation antiseizure medications (ASMs) and children of healthy women (HW).
- Breastfeeding Benefit in WWE cohort: To evaluate the independent association between breastfeeding duration and child neurodevelopmental outcomes within the WWE cohort, specifically testing for a duration-response relationship.

1.2 Secondary Objectives

- WWE vs. HW Group Differences:
  - Subdomain Analysis: To evaluate group differences and breastfeeding effects across specific ABAS-3 subdomains: Conceptual, Social, and Practical functioning.
  - General Intelligence: To compare the General Conceptual Ability (GCA) from the DAS-2 between WWE and HW groups.
  - Autism Disorder Risk Screening: To assess the risk of autism spectrum disorder (SRS-2).
- Breastfeeding Duration Benefit: To test if breastfeeding duration is independently associated with these secondary outcomes within the WWE cohort

2. STUDY DEFINITIONS AND DERIVED VARIABLES

2.1 Outcome Variables

All primary scores are transformed to a common scale (Mean = 100; SD = 15) to ensure comparability across different instruments.

- Primary Language Outcome: Verbal Index Score (VIS):
  - The VIS was designed to combine several well standardized verbal tests in order to integrate a range of verbal assessments. It is a composite score calculated as the average of available Word Definitions and Verbal Similarities subtests from the Differential Ability Scales-2nd Edition (DAS-2), Expressive One-Word Picture Vocabulary Test-4th Edition (EOWPVT-4), Phonological Processing, Comprehension of Instructions, and Sentence Repetition subtests from the Neuropsychological Assessment-2nd Edition (NEPSY-2), and Peabody Picture Vocabulary Test-4th Edition (PPVT-4).
  - If more than one subscale score is missing, the VIS is set to missing.
- Primary Behavioral Outcome: ABAS-3 GAC:
  - The General Adaptive Composite (GAC) summarizes performance across all adaptive skill areas excluding work.
- Secondary Outcome: ABAS-3 Behavioral Outcome Subdomains:
  - Conceptual: Evaluate communication, Functional Academics, and Self-Direction.
  - Social: Evaluate Leisure and Social skills.

© 2026 Li Y et al. *JAMA Network Open*.

  - Practical: Evaluate Community Use, School/Home Living, Health and Safety, and Self-Care.

  These standard scores are transformed to a common scale (Mean = 100; SD = 15) to ensure comparability across different instruments.
- Secondary Outcome: General Conceptual Ability/General Intelligence (DAS-2 GCA):
  - The General Conceptual Ability (GCA) score from the DAS-2 serves as the primary measure of the child's overall IQ.
  - The GCA is a composite score that provide a global measure of cognitive functioning.
- Secondary Outcome: Nonverbal Ability
  - Nonverbal ability is primarily measured using the DAS-2 Special Nonverbal Reasoning Cluster score.
  - This score assesses visual-spatial processing and fluid reasoning skills, providing a comparison to verbal index scores to evaluate potential patterns of cerebral lateralization.

  These standard scores are transformed to a common scale (Mean = 100; SD = 15) to ensure comparability across different instruments
- Secondary Outcome: Autism Risk Screening:
  - Defined as an SRS-2 Total T-Score ≥ 70.

2.2 Exposure Variables:

- Breastfeeding Duration: Categorized into three tiers: None, Short-term (greater than 0 to less than 6 months), and Extended (6 months or longer).
- ASM Exposure Regimens: Grouped as Levetiracetam monotherapy, Lamotrigine monotherapy, other monotherapy, and Polytherapy; or monotherapy vs. polytherapy.

3. COVARIATES

The selection of covariates for adjusted models is guided by established literature on factors potentially influencing child neurodevelopment

- Maternal Factors:
  - Maternal Socio-Demographic Factors:
    - Age and IQ: Analyzed as continuous variables
    - Educational Attainment (3 levels): No college degree, College degree (not advanced), and College degree (advanced).
    - Employment Status (3 levels): Unemployed, Part-time, and Full-time.
    - Household Income (6 levels): < $24,999; $25,000–49,999; $50,000–74,999; $75,000–99,999; >= $100,000/year; or Subject desires not to answer.
  - Maternal Clinical and Prenatal Factors:
    - Pregnancy Planning (3 levels): Planned, Unplanned/Welcome, and Unplanned/Unwelcome.
    - Prenatal Exposures: Smoking during pregnancy analyzed as binary variables (Any/None). Alcohol during pregnancy analyzed as binary variables (Any/None)
    - Folate Dose (5 levels): None; >0–0.4 mg; >0.4–1.0 mg; >1.0–4.0 mg; and >4.0 mg/d.
    - Mood, Stress and Sleep Measures: calculated for (a) from enrollment throughout pregnancy, and (b) Post-delivery through mother's 6-year post-delivery visit
      - Beck Anxiety Inventory (BAI) scores.

© 2026 Li Y et al. *JAMA Network Open*.

        - Beck Depression Inventory-2 (BDI-2) scores.
        - Perceived Stress Scale-14 (PSS-14) scores.
        - Pittsburgh Sleep Quality Index (PSQI) scores.
- Child and Perinatal Factors:
    - Sex: Binary (Male, Female).
    - Race: Three levels (White, Black or African American, and Other/Unknown).
    - Ethnicity: Binary (Hispanic or Latino, Non-Hispanic/Latino).
    - Perinatal Factors:
        - Gestational age at birth (weeks).
        - Birthweight (kg).
        - Small for Gestational Age (SGA).
- Epilepsy-Specific Factors:
    - Epilepsy Type (3 types): Focal, Generalized, and Unclassified/Mixed.
    - Seizure Frequency: Total number of convulsive seizures during pregnancy.
    - Fetal ASM Ratio: Quantified by the mother's maximum observed 3rd-trimester blood concentration divided by the therapeutic upper limit; for polytherapy, the sum of these ratios across all measured ASMs is utilized.

4. STATISTICAL METHODS

4.1 General Approach

- Demographic and clinical characteristics between groups (WWE vs. HW) are compared using chi-square or Fisher exact tests for categorical variables and t tests or Wilcoxon rank-sum tests for continuous variables.

4.2 Model Selection

- The initial base models evaluated the primary exposures (cohort group and breastfeeding duration) alongside their interaction term. Because the interaction between cohort group and breastfeeding duration was not statistically significant, it was removed from the base null models prior to stepwise variable selection to allow for the accurate estimation of main effects. The final models presented above were derived using a stepwise selection algorithm (entry $P < .05$, removal $P \geq .10$) to select appropriate confounding covariates from the candidate list. For Primary Analysis 1 (WWE vs. HW), both breastfeeding duration and cohort group were forced into all steps of the modeling process. For Primary Analysis 2 (restricted to the WWE cohort), breastfeeding duration was forced into all steps. Coefficients represent the adjusted point estimates from the final multivariable linear regression models, which were used to calculate the adjusted least-squares means
- Fixed Covariates: Following established cohort standards, breastfeeding duration (or mother's study group) are retained as fixed a priori variables in adjusted models to ensure clinical validity and consistency.

4.3 Sensitivity Analyses

- ASM Subgroup Stratification: Outcomes are stratified by specific antiseizure medication (ASM) regimens—including levetiracetam monotherapy, lamotrigine monotherapy, other monotherapy and polytherapy—to evaluate potential medication-specific effects.
- Twin Correction: To account for the non-independence of data within the cohort, primary analyses are repeated using one randomly selected child from each twin pair.

© 2026 Li Y et al. *JAMA Network Open*.

- Model Fit and Variable Selection Sensitivity (AIC): To ensure the robustness and parsimony of our multivariable regression models—particularly given the sample size constraints within specific exposure stratifications—model fit was evaluated using the Akaike Information Criterion (AIC). Base null models (containing only the primary exposures of cohort group and breastfeeding duration) were systematically compared to the final models generated by the stepwise selection algorithm. A reduction in the AIC value, coupled with an increase in Adjusted R^2 (or Pseudo R^2 for binary outcomes), was required to confirm that the inclusion of specific covariates improved overall model fit without overfitting the data. This methodology justifies the specific adjustment variables utilized across the different neurodevelopmental outcome measures.
- To assess the robustness of our primary findings to baseline covariate imbalances, we conducted a sensitivity analysis utilizing a propensity score-based approach. Rather than traditional 1:1 matching or quintile stratification—which can truncate sample sizes or leave residual confounding within strata in imbalanced cohorts—we employed Inverse Probability of Treatment Weighting (IPTW) coupled with a doubly robust estimator to utilize the full analytic cohort while achieving optimal covariate balance. Stabilized IPTW weights were computed using logistic regression to balance baseline maternal and child characteristics across exposure groups. A generalized linear model was then fit using IPTW-weighted maximum likelihood estimation, followed by standardization (the G-formula) to compute the average treatment effect. This approach yields an unbiased estimate if either the propensity score model or the outcome regression model is correctly specified

4.4 Power and Minimum Detectable Effect Size (MDES)

- The *a priori* power calculations detailed in the parent MONEAD protocol targeted 80% power to detect a 0.5 standard deviation (SD) difference between the cohorts. To ensure adequate statistical power for the specific primary and secondary outcomes of the present study—as well as the specific stratifications by breastfeeding duration—a Minimum Detectable Effect Size (MDES) analysis is conducted based on final analytic sample sizes. The MDES is calculated assuming a standard continuous distribution (SD = 15), an alpha level of .05, and 80% power, to confirm that the study retains the capacity to detect differences below the originally targeted 0.5 SD threshold. The MDES calculations were performed to verify adequate statistical power for the present study's specific outcomes and stratifications, the full details of which are available in the Supplementary Results.

Analyses are conducted using Python (version 3.11), SPSS (version 28), and R (version 4.6), with statistical significance defined by a two-sided alpha of .05.

© 2026 Li Y et al. *JAMA Network Open*.
