# Supplementary material for: Breastfeeding and Neurodevelopmental Outcomes in Children of Women Taking Antiseizure Medications
Source: JAMA Netw Open. 2026 Sep 30;9(9):e2637040. doi: 10.1001/jamanetworkopen.2026.37040 (PMC13628311; doi:10.1001/jamanetworkopen.2026.37040)
Supplement: Supplementary file 2 [file jamanetwopen-e2637040-s002.pdf]

Supplemental Online Content: Nonauthor Collaborators

*Indicates required information. Only first name, last name, and suffix will appear in PubMed.

**Group Name(s):* MONEAD Investigator Group**					
First Name and Middle Initial(s)*	Last Name*	Suffix (eg, Jr, III)*	Academic Degrees	Institution	Role or Contribution, eg, chair, principal investigator
Maurice	Druzin		MD	Stanford University	Co-Director of OB Core
Richard	Finnell		PhD	Baylor College of Medicine	Consultant on Teratogenicity
Gregory	Holmes		MD	University of Vermont	Consultant and Executive Committee Member
Frederick T.	McElrath		MD	Brigham & Women's Hospital	Co-Director of OB Core
Lorene	Nelson		PhD	Stanford University	Consultant and Executive Committee Member
Zachary	Stowe		MD	University of Wisconsin	Director of Psychiatric Core
Linda	Van Marter		MD	John Hopkins University	Director of Neonatal Core
Peter	Wells		PharmD	University of Toronto	Consultant and Executive Committee Member
Mark	Yerby		MD	Oregon Health Sciences University	Consultant and Executive Committee Member
Eugene	Moore		BS	Emory University	Multisite Research Project Manager
Ryan	May		PhD	The Emmes Company	Former Muliti PI
Dominic	Ippolito		MS	The Emmes Company	Project Manager
Lisa	Davis		BA	The Emmes Company	Administrative Coordinator
Nilay	Shah		MD	The Emmes Company	Medical Monitor
Brenda	Leung		BS	The Emmes Company	Programmer Analyst
Mark	Friedman		MS	The Emmes Company	Clinical Systems Analyst
Traci	Sheer		BA	The Emmes Company	Data Manager/Protocol Monitor
Yue	Wang		MS	The Emmes Company	SAS Programmer
Suzanne	Strickland		MD	Augusta University	Site PI
Erin	Latif		MD	Augusta University	OB co-Investigator
Yong	Park		MD	Augusta University	OB co-Investigator
Delmaris	Acosta-Cotte		MA	Augusta University	Site Neuropsychologist
Patty	Ray		PhD	Augusta University	Research Assistant
Kirsten	Cleary		MD	Columbia University	OB co-Investigator
Joyce	Echo		PhD	Columbia University	Site Neuropsychologist
Annette	Zygmunt		PhD	Columbia University	Site Neuropsychologist
Camilla	Casadei		BA	Columbia University	Research Assistant
Mary	Dolan		MD	Emory University	OB co-Investigator

Supplemental Online Content: Nonauthor Collaborators

*Indicates required information. Only first name, last name, and suffix will appear in PubMed.

First Name and Middle Initial(s)*	Last Name*	Suffix (eg, Jr, III)*	Academic Degrees	Institution	Role or Contribution, eg, chair, principal investigator
Kim	Ono		PhD	Emory University Children's Healthca	Site Neuropsychologist
Donald	Bearden		PhD	Emory University Children's Healthca	Site Neuropsychologist
Christine	Ghilian		PhD	Emory University Children's Healthca	Site Neuropsychologist
Diane	Teagarden		MSN	Emory University	Co-Investigator
Melanee	Newman		RN	Emory University	Research Assistant
Paul	McCabe		MD	Geisinger Clinic	Retired Site PI
Michael	Paglia		MD	Geisinger Clinic	OB co-Investigator
Rosemarie	Delucca		RN	Geisinger Clinic	Research Assistant
Kristina	Blessing		MSW	Geisinger Clinic	Research Assistant
Katrina	Boyer		PhD	Boston Children's Hospital	Site Neuropsychologist
Ellen	Hanson		PhD	Boston Children's Hospital	Site Neuropsychologist
Amy	Young		PsyD	Boston Children's Hospital	Site Neuropsychologist
Paige	Hickey		BS	Boston Children's Hospital	Site Psychometrist
Jolie	Strauss		MA	Boston Children's Hospital	Site Psychometrist
Hayley	Madeiros		BS	Boston Children's Hospital	Site Psychometrist
Li	Chen		BA	Brigham & Women's Hospital	Research Assistant
Stephanie	Allien		PAC	Brigham & Women's Hospital	Research Physician's Assistant
Yvonne	Sheldon		RN	Brigham & Women's Hospital	Neonatal Research Nurse
Taylor	Weinau		BS	Brigham & Women's Hospital	OB Research Assistant
Elizabeth	Shashkova		BS	Brigham & Women's Hospital	Research Assistant
Melanie	Choe		MS	Brigham & Women's Hospital	Research Assistant
Gregory L.	Barkley		MD	Henry Ford Hospital	Site PI
Marianna	Spanaki-Varelas		MD	Henry Ford Hospital	OB Co-Investigator
Andrea	Thomas		MS	Henry Ford Hospital	Site Neuropsychologist
Jules	Constantinou		MD	Henry Ford Hospital	Co-Investigator
Nazin	Mahmood		MD	Henry Ford Hospital	Co-Investigator
Vibhangini	Wasade		MD	Henry Ford Hospital	Co-Investigator
Shailaja	Gaddam		MD	Henry Ford Hospital	Co-Investigator
Andrew	Zillgitt		DO	Henry Ford Hospital	Co-Investigator
Taimur	Anwar		MD	Henry Ford Hospital	OB Co-Investigator

Supplemental Online Content: Nonauthor Collaborators

*Indicates required information. Only first name, last name, and suffix will appear in PubMed.

First Name and Middle Initial(s)*	Last Name*	Suffix (eg, Jr, III)*	Academic Degrees	Institution	Role or Contribution, eg, chair, principal investigator
Carla	Sandles		CCRP	Henry Ford Hospital	Research Assistant
Theresa	Holmes		BA	Henry Ford Hospital	Research Assistant
Emily	Johnson		MD	Johns Hopkins University	Site PI
Gregory	Krauss		MD	Johns Hopkins University priorSite PI	Prior Site PI
Shari	Lawson		MD	Johns Hopkins University	OB Co-Investigator
Alison	Pritchard		PhD	John Hopkins University	Site Neuropsychologist
Matthew	Ryan		MS	John Hopkins University	Site Neuropsychologist
Pam	Coe		MS	John Hopkins University	Research Assistant
Katie	Reger		PhD	Minnesota Epilepsy Group	Site Neuropsychologist
Jenny	Pohlman		MBS	Minnesota Epilepsy Group	Research Assistant
Alisha	Olson		RN	Minnesota Epilepsy Group	Research Assistant
Anna	Steele			Minnesota Epilepsy Group	Site Neuropsychologist
Hadley	Lange			Minnesota Epilepsy Group	Site Neuropsychologist
Patricia	Chang		MD	Minnesota Epilepsy Group	OB co-Investigator
Sarah	Ellis		BS	Minnesota Epilepsy Group	Research Assistant
William	Schweizer		MD	New York University	OB Co-Investigator
Chris	Morrison		PhD	New York University	Site Neuropsychologist
William	MacAllister		PhD	New York University	Site Neuropsychologist
Tobi	Clements		BA	New York University	Research Assistant
Hima Bindu Tam	Tam		MD	Northwell Health	OB Co-Investigator
Yael Cukier	Cukier		PhD	Northwell Health	Site Neuropsychologist
Erica Meltzer	Meltzer		MD	Northwell Health	Site Neuropsychologist
Jacqueline Helcer	Helcer		PhD	Northwell Health	Site Neuropsychologist
Connie Lau	Lau		MS	Northwell Health	Research Assistant
William	Grobman		MD	Northwestern University	OB Co-Investigator
Joseph	Coda		PsyD	Northwestern University	Site Neuropsychologist
Emily	Miller		MD	Northwestern University	OB Co-Investigator
Irena	Bellinski		RN	Northwestern University	Research Assistant
Elizabeth	Cunningham		MPH	Northwestern University	Research Assistant
Casey	Krueger		PhD	Stanford Healthcare	Site Neuropsychologist
Jordan	Seliger		MA	Stanford University	Research Assistant

Supplemental Online Content: Nonauthor Collaborators

*Indicates required information. Only first name, last name, and suffix will appear in PubMed.

First Name and Middle Initial(s)*	Last Name*	Suffix (eg, Jr, III)*	Academic Degrees	Institution	Role or Contribution, eg, chair, principal investigator
Jennifer	DeWolfe		DO	University of Alabama at Birmingham	Site PI
John	Owen		MD	University of Alabama at Birmingham	OB Co-Investigator
Matthew	Thompson		PsyD	University of Alabama at Birmingham	Site Neuropsychologist
Cheryl	Hall		LPN	University of Alabama at Birmingham	Research Assistant
David	Labiner		MD	Arizona Health Sciences Center	Site PI
James	Maciulla		MD	University of Arizona	OB Co-Investigator
Jennifer	Moon		PsyD	University of Arizona	Site Neuropsychologist
Kayla	Darris		BA	University of Arizona	Research Assistant
Michael	Privitera		MD	University of Cincinnati	Co-Investigator
Kellie	Flood-Schaffer		MD	University of Cincinnati	OB Co-Investigator
George	Jewell		PhD	University of Cincinnati	Site Neuropsychologist
Lucy	Mendoza		CCRP	University of Cincinnati	Research Assistant
Naymee	Velez-Ruiz		MD	University of Miami	Site PI
Enrique	Serrano		MD	University of Miami	Co-Investigator
Yasin	Salih		MD	University of Miami	OB Co-Investigator
Gustavo	Rey		PhD	University of Miami	Site Neuropsychologist
Christin	Bermudez		PhD	University of Miami	Site Psychometrist
Michelle	Miranda		PhD	University of Miami	Site Psychometrist
Pedro	Figueredo		MD	University of Miami	Research Assistant
Anto	Bagic		MD	University of Pittsburgh	Site PI
Alexandra	Urban		MD	University of Pittsburgh	Co-Investigator
Satya	Gedela		MD	University of Pittsburgh	Co-Investigator
Christina	Patterson		MD	University of Pittsburgh	Co-Investigator
Arundhathi	Jeyabalan		MD	University of Pittsburgh	OB Co-Investigator
Krestin	Radonovich		PhD	University of Pittsburgh	Site Neuropsychologist
Melissa	Sutcliffe		PhD	University of Pittsburgh	Site Neuropsychologist
Susan	Beers		PhD	University of Pittsburgh	Site Neuropsychologist
Carrie	Wiles		MS	University of Pittsburgh	Site Neuropsychologist
Sandra	Alhaj		BS	University of Pittsburgh	Research Assistant
Alice	Stek		MD	University of Southern California	OB Co-Investigator
Sonia	Perez		PhD	University of Southern California	Site Neuropsychologist

Supplemental Online Content: Nonauthor Collaborators

*Indicates required information. Only first name, last name, and suffix will appear in PubMed.

First Name and Middle Initial(s)*	Last Name*	Suffix (eg, Jr, III)*	Academic Degrees	Institution	Role or Contribution, eg, chair, principal investigator
Rachel	Sierra		RC	University of Southern California	Research Assistant
John W.	Miller		MD	University of Washington	Previous Site PI
Jennie	Mao		MD	University of Washington	OB Co-Investigator
Michelle	Kim		PhD	University of Washington	Site Neuropsychologist
Andrea	Cheng-Hakimian		MD	University of Washington	Co-Investigator
Jennifer	Steele		BA	University of Washington	Research Assistant
Lamar	Parker		MD	Wake Forest University Health Scien	OB Co-Investigator
Melissa	Morris		MA	Wake Forest University Health Scien	Site Neuropsychologist
Jessica	Dimos		BS	Wake Forest University Health Scien	Research Assistant
Danielle	Culbreth		MA		Project Traveling Neuropsychologist
