# Supplementary material for: Breastfeeding and Neurodevelopmental Outcomes in Children of Women Taking Antiseizure Medications
Source: JAMA Netw Open. 2026 Sep 30;9(9):e2637040. doi: 10.1001/jamanetworkopen.2026.37040 (PMC13628311; doi:10.1001/jamanetworkopen.2026.37040)
Supplement: Supplementary file 3 [file jamanetwopen-e2637040-s003.pdf]

Data Sharing Statement

Li. Breastfeeding and Neurodevelopmental Outcomes in Children of Women Taking Antiseizure Medications. *JAMA Netw Open*. Published September 30, 2026. doi:10.1001/jamanetworkopen.2026.37040

Data
Data available: Yes
Data types: Deidentified participant data
How to access data: lyi@stanford.edu
When available: With publication

Supporting Documents
Document types: Other (please specify)
Additional Information: deidentified data
How to access documents: deidentified data
When available: With publication

Additional Information
Who can access the data: researchers whose proposed use of the data has been approved
Types of analyses: for any purpose
Mechanisms of data availability: with a signed data access agreement
Any additional restrictions: completion of ongoing analyses
